# A randomized, double-blind, active-control trial to evaluate the efficacy and safety of a three day course of tafenoquine monotherapy for the treatment of *Plasmodium vivax* malaria

**DOI:** 10.1371/journal.pone.0187376

**Published:** 2017-11-09

**Authors:** Mark M. Fukuda, Srivicha Krudsood, Khadeeja Mohamed, Justin A. Green, Sukhuma Warrasak, Harald Noedl, Ataya Euswas, Mali Ittiverakul, Nillawan Buathong, Sabaithip Sriwichai, R. Scott Miller, Colin Ohrt

**Affiliations:** 1 Armed Forces Research Institute of Medical Science, Bangkok, Thailand; 2 Faculty of Tropical Medicine, Mahidol University, Bangkok, Thailand; 3 GlaxoSmithKline Research and Development, Uxbridge, Middlesex, United Kingdom; 4 Ramathibodi Hospital, Mahidol University, Bangkok, Thailand; 5 Walter Reed Army Institute of Research, Silver Spring, Maryland, United States of America; George Washington University School of Medicine and Health Sciences, UNITED STATES

## Abstract

**Background:**

Tafenoquine is an investigational 8-aminoquinoline for the prevention of *Plasmodium vivax* relapse. Tafenoquine has a long half-life and the potential for more convenient dosing, compared with the currently recommended 14-day primaquine regimen.

**Methods:**

This randomized, active-control, double-blind trial was conducted in Bangkok, Thailand. Seventy patients with microscopically confirmed *P*. *vivax* were randomized (2:1) to tafenoquine 400 mg once daily for 3 days or 2500 mg total dose chloroquine phosphate (1500 mg chloroquine base) given over 3 days plus primaquine 15 mg daily for 14 days. Patients were followed to day 120.

**Results:**

Day 28 adequate clinical response rate in the per-protocol population was 93% (40/43) (90%CI 83–98%) with tafenoquine, and 100% (22/22) (90%CI 87–100%) with chloroquine/primaquine. Day 120 relapse prevention was 100% (35/35) with tafenoquine (90%CI 92–100%), and 95% (19/20) (90%CI 78–100%) with chloroquine/primaquine. Mean (SD) parasite, gametocyte and fever clearance times with tafenoquine were 82.5 h (32.3), 49.1 h (33.0), and 41.1 h (31.4) versus 40.0 h (15.7), 22.7 h (16.4), and 24.7 h (17.7) with chloroquine/primaquine, respectively. Peak methemoglobin was 1.4–25.6% (median 7.4%, mean 9.1%) in the tafenoquine arm, and 0.5–5.9% (median 1.5%, mean 1.9%) in the chloroquine/primaquine arm. There were no clinical symptoms of methemoglobinemia in any patient.

**Discussion:**

Although there was no difference in efficacy in this study, the slow rate of parasite, gametocyte and fever clearance indicates that tafenoquine should not be used as monotherapy for radical cure of *P*. *vivax* malaria. Also, monotherapy increases the potential risk of resistance developing to this long-acting agent. Clinical trials of single-dose tafenoquine 300 mg combined with standard 3-day chloroquine or artemisinin-based combination therapy are ongoing.

**Trial registration:**

Clinicaltrials.gov NCT01290601

## Introduction

*Plasmodium vivax* causes around half of all malaria cases occurring outside Africa with an estimated 2.5 billion persons at risk of infection [1]. Most cases of *P*. *vivax* malaria occur in South-East Asia (72.5%), the Eastern Mediterranean (10.9%) and East Africa (10.1%), but also in South America (3.6%) and the Western Pacific (1.4%) [1]. Mortality data for *P*. *vivax* are sparse, with estimates ranging between 4% and 39% of all malaria-related deaths outside sub-Saharan Africa [1]. However, deaths from P. vivax could be substantially higher [2], and *P*. *vivax* cannot be considered benign [3].

The control of *P*. *vivax* is challenging because of a number of factors related to its lifecycle and biology [4]. A relapsing human malaria, *P*. *vivax* has a dormant liver stage, the hypnozoite. Following the initial infection of the liver by *P*. *vivax* sporozoites, the parasite transforms either into actively dividing schizonts which cause the blood stage infection and clinical symptoms, or hypnozoites, which may reactivate weeks, months, or even years later, potentially causing multiple clinical relapses from a single infective bite. *P*. *vivax* is a particular challenge in South-East Asia and the Pacific where chloroquine-resistant strains are prevalent and the relapse pattern resembles that of the laboratory ‘Chesson’ strain, characterized by multiple, rapid relapses in most patients [5].

Unlike *P*. vivax blood stages, hypnozoites are undetectable. They represent a reservoir for future malaria transmission because *P*. *vivax* relapses may occur in new areas without ongoing transmission and allow the parasite to survive seasonal or environmental conditions unfavorable to transmission. In addition, *P*. *vivax* gametocytemia occurs earlier relative to *P*. *falciparum*, even before any clinical symptoms emerge, further increasing the risk of onward transmission [4]. Thus, in addition to clearing blood stages, eliminating *P*. *vivax* hypnozoites (i.e. ‘radical cure’) is important both for decreasing relapse morbidity for the individual patient and reducing transmission potential to others.

Currently, the 8-aminoquinoline primaquine is the only approved hypnozoitocide drug, given in combination with a blood schizonticide, such as chloroquine or an artemisinin-based combination therapy [ACT]). The World Health Organization recommended dosing regimen is standard blood schizonticidal therapy for 3 days plus primaquine 0.25–0.5 mg/kg bodyweight daily for 14 days [6]. However, adherence to this regimen is problematic. As the patient usually feels better after the 3-day blood schizonticide, they often fail to complete the full 14-day primaquine course, compromising field effectiveness [7].

Tafenoquine is an 8-aminoquinoline discovered and developed by the Walter Reed Army Institute of Research, now being co-developed by GlaxoSmithKline and Medicines for Malaria Venture (MMV) for the prevention of *P*. *vivax* relapse. The current investigational regimen is tafenoquine 300 mg single dose plus standard 3-day chloroquine [8]. The key advantage of tafenoquine over primaquine is its long half-life of around 14 days, offering the potential for more convenient dosing regimens and improved therapy compliance compared with primaquine [9].

There have been many previous studies investigating tafenoquine at various doses and regimens for *P*. *vivax* treatment and prophylaxis [10–16]. However, although tafenoquine blood schizonticidal activity was shown in non-human primate models [17, 18], and gametocytocidal activity was suspected, as observed for primaquine, neither the schizonticidal nor gametocytocidal activity of tafenoquine had been tested in humans against *P*. *falciparum* or *P*. *vivax*. This Phase II study evaluated the efficacy of high-dose tafenoquine monotherapy (1200 mg over three days) for the radical cure of *P*. *vivax* malaria. The study was conducted under an NIH Challenge Grant ‘Tafenoquine, a Novel Drug for Malaria Prevention and Control’.

Both 8-aminoquinolines, primaquine and tafenoquine, cause hemolysis in glucose-6-phosphate dehydrogenase (G6PD)-deficient individuals [19, 20]. Both agents also cause usually mild increases methemoglobin levels [14, 21]. In a phase III prophylaxis trial in Australian soldiers, possible eye and renal safety findings were noted with tafenoquine [22]. The study reported in this paper was one of two trials that evaluated whether such findings had clinical significance [23]. Ophthalmic findings from the current study are to be reported separately.

## Materials and methods

### Ethical statement

The protocol was approved by the Ethical Committee, Faculty of Tropical Medicine, Mahidol University, Bangkok, Thailand on 23^rd^ April 2003, the Ministry of Public Health, Nonthaburi, Thailand, and the Human Subjects Research Review Board, U.S. Army Medical Research and Material Command, Office of Regulatory Compliance and Quality, Fort Detrick, MD, USA. The study was done in accordance with Good Clinical Practice and written informed consent was obtained from all patients before participation. This study was monitored by the U.S. Army Medical Research and Material Command Quality Assurance Office, (Fort Detrick, Maryland, USA) and the Division of Microbiology and Infectious Diseases, National Institutes of Health (Bethesda, Maryland, USA). The protocol can be obtained from the corresponding author. The trial was registered at Clinicaltrials.gov (identifier NCT01290601) in February 2011. This study was conducted before clinical trial registration became a requirement for publication and was, therefore, registered retrospectively. The authors confirm that all ongoing and related trials for this drug/intervention are registered. All relevant data are within the paper.

### Study design and treatment

**Fig 1:**
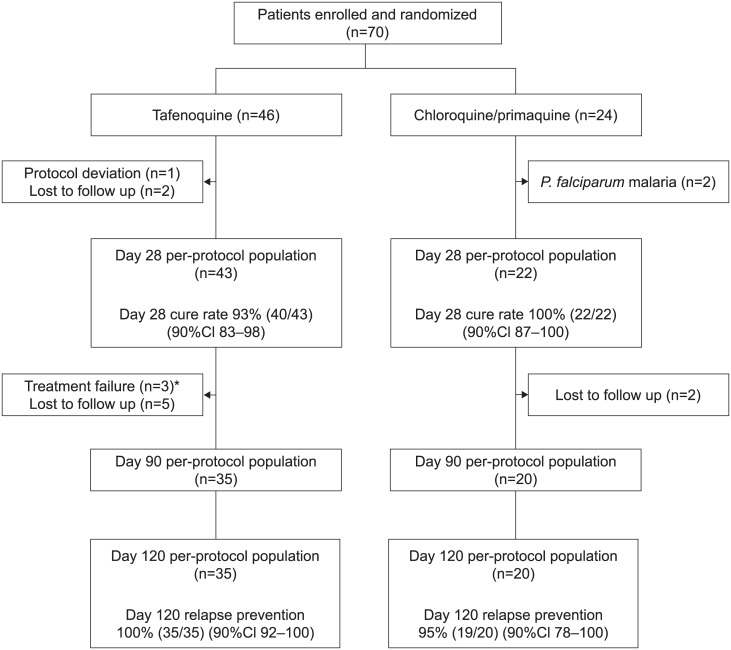
Study design, patient disposition and main efficacy outcomes. *The three patients with early treatment failure (at day 7) in the tafenoquine group cleared parasitemia spontaneously on day 8 without additional treatment, and were relapse free for the duration of their follow-up (until day 28 for one patient, day 60 for the second and day 120 for the third).

This was a randomized, active-control, double-blind, double-dummy study conducted from 15^th^ September 2003 to 10^th^ January 2005 at the Bangkok Hospital for Tropical Disease (Fig 1).

There were to be two sequential cohorts: cohort 1 was 400 mg tafenoquine for three days and comparator; cohort 2 was planned to be tafenoquine 600 mg as a single dose and comparator, but was not executed because of slow parasite clearance in cohort 1.

**Fig 2:**
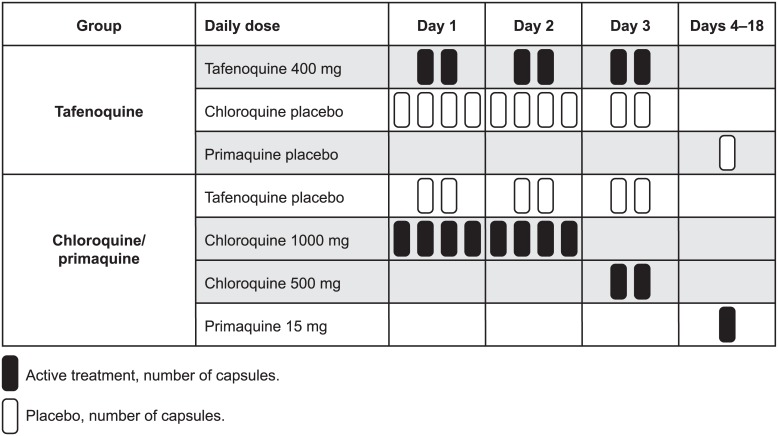
Dosing schedule. The planned enrollment for this study was 70 patients in each of two cohorts in order to yield at least 60 evaluable patients in each cohort. Using a 2:1 randomization ratio and assuming a true success rate on treatment of at least 98%, the sample size of 40 evaluable tafenoquine patients provided 90% power to show that the lower limit of the one-sided 95% confidence interval for the day 28 cure rate was above 85%.

Treatment allocation was based on a computer generated block randomization list (block size 6). Eligible patients were randomized to active treatment with either tafenoquine 400 mg for three days (GlaxoSmithKline, two 200 mg capsules per day); or chloroquine phosphate 1000 mg for two days (AstraZeneca UK Ltd, four 250 mg capsules per day; 600 mg chloroquine base) and chloroquine phosphate 500 mg for one day (two 250 mg capsules; 300 mg chloroquine base) followed by 15 mg primaquine base for 14 days (Muir Pty Ltd, one capsule per day, given with food). Matched placebos were given as shown in Fig 2.

### Participants

Eligible participants were male or female patients 20–60 years old with microscopically confirmed *P*. *vivax* infection, defined as the presence of *P*. *vivax* asexual stage parasites on a blood smear (parasite density >500 and <200,000/μL). Patients were excluded if they were pre-menarchal, pregnant or lactating, unwilling or unable to comply with recognized contraceptive methods, had mixed microscopically determined *Plasmodium* infection, severe vomiting or any medical condition that could interfere with drug absorption, clinically significant illness or an abnormal laboratory value, including evidence of renal dysfunction or G6PD deficiency. Because the study also assessed the potential ophthalmic effects of tafenoquine, subjects were also excluded if they had previous eye surgery, corneal or retinal abnormalities including risk for acute angle closure glaucoma, or used concomitant medications likely to affect renal or ophthalmic function. Patients allergic to 8-aminoquinolines, and those who had used other anti-malarial drugs or another investigational product in the previous 30 days were also excluded.

### Study procedures

Study participants were hospitalized from day 0 to day 28 with follow up on days 60, 90, 120 or at withdrawal, and were to remain in a malaria free region until day 90. A medical history was taken and vital signs recorded at screening. Vital signs were taken daily until day 7 then days 14 and 28 and at each follow-up visit. Body temperature was measured every 12 hours (±2 hours) after the baseline measurement through day 7. All study medications were given by direct observation by study staff.

Malaria blood smears and routine laboratory tests were performed at the Bangkok Hospital for Tropical Disease (HTD) and Armed Forces Research Institute of Medical Sciences (AFRIMS), Bangkok, Thailand. Thick and thin blood smears were obtained on day 0 and then every 12 hours (± 2 hours) up to and including day 7 until the blood smear became negative for two consecutive smears, then daily until day 7, then on days 14 and 28 and follow up visits at days 60, 90 and 120. Patients were asked to return to the clinic if they developed signs or symptoms of malaria during the follow-up phase. Additional smears were done each day a patient exhibited signs or symptoms of suspected malaria. Microscopically confirmed treatment failures were withdrawn from the study and given appropriate anti-malarial rescue medication as per the Bangkok Hospital for Tropical Diseases treatment practice. Patients who developed *P*. *falciparum* malaria before day 28 were withdrawn from the study and were not considered evaluable for efficacy endpoints.

Microscopic examination of blood smears was conducted by two independent microscopists, blinded to treatment allocation and each other’s results, who examined 200 oil-immersion fields (magnification x1000) on Field’s-stained blood smears. Any discrepancies (positive/negative; species diagnosis; or >2-fold difference in parasite density) were resolved by a third blinded, senior study microscopist. The third reading was considered final. Parasite densities were calculated based on a count of parasites per 1000 red blood cells in a thin film or per 200 white blood cells in a thick film.

Adverse events were evaluated daily until day 29, then at each follow-up visit and were coded using MedRA dictionary version 8.1. Clinical chemistry was performed at days 0, 3, 7, 14, 21, 28, and 90. Blood samples for hematology were collected at days 0, 3, 7, and 14. Blood samples for methemoglobin were collected at days 0, 3, 7, and 21. Renal assessments included serum creatinine and urinalysis and were collected at days 0, 7, and 28. Two independent data monitoring committees (IDMC) were chartered; one for efficacy and routine safety, and one for interpretation of the detailed ophthalmic data (reported separately).

Tafenoquine plasma concentrations were determined by Quintiles Limited (Edinburgh, Scotland) under the direction of Worldwide Bioanalysis Department, DMPK, GlaxoSmithKline Pharmaceuticals, UK. Plasma samples were collected from recipients according to a schedule designed for population pharmacokinetics.

### Outcomes

The primary efficacy endpoint was day 28 adequate clinical response, defined as parasitological clearance throughout the follow-up period until day 28 without previous early treatment failure (defined as parasitemia on day 7) or late treatment failure (parasitemia recurring after day 7 up to and including day 28).

Secondary endpoints were the proportion of patients without *P*. *vivax* re-emergence of parasitemia at days 60, 90, and 120; parasite clearance time and gametocyte clearance time, defined as the interval between starting treatment and the first of two consecutive negative blood smears for *P*. *vivax* parasites/gametocytes; fever clearance time, defined as the time taken from treatment start for the body temperature to decrease to 37.2°C and remain at or below this level for a minimum of 24 h. Note that gametocyte clearance time and fever clearance time calculations assumed that patients with fever or gametocytes at any time actually had fever or gametocytes present at baseline.

Tafenoquine safety was evaluated based on the frequency and severity of adverse events and abnormal values of clinical chemistry, hematology, methemoglobin and urinalysis parameters. Tafenoquine population pharmacokinetics was also reported.

### Statistical analysis

No formal comparison was planned between treatment groups. The primary objective of the study was to assess the efficacy of the tafenoquine doses alone. The chloroquine/primaquine treatment arm was included as a reference. For the purposes of estimation, of a one-sided 90% confidence interval (90%CI) was calculated using the Clopper-Pearson ‘exact’ methodology for the day 28 cure rate in each treatment arm. Secondary efficacy endpoints and all safety data were summarized descriptively.

The day 28 per-protocol population was the primary population for efficacy analyses, including all randomized patients who completed all scheduled assessments to day 28, who received all drug treatments and who were compliant with the protocol up to day 28. All patients who were treatment failures, or who had withdrawn from the study, or experienced recurrence (recrudescence or relapse) by day 28 were not required to have attended subsequent visits for inclusion in this population. Definitions for the per-protocol population at days 7, 60, 90 and 120 were similar, though criteria were specific for that time point. The safety population and the intent-to-treat population included all randomized patients who received at least one dose of study medication.

## Results

### Patient disposition and baseline characteristics

Seventy subjects met the eligibility requirements; 46 were randomized to receive tafenoquine 400 mg and 24 to receive chloroquine/primaquine (Fig 1). The reasons for study withdrawal were similar between groups, with the exception of two patients receiving chloroquine/primaquine who developed *P*. *falciparum* malaria (days 8 and 24). No study discontinuation was attributable to adverse events related to study medication. Baseline characteristics were similar between groups (Table 1).

**Table 1:** Baseline characteristics of the study participants in intention-to-treat/safety population.

Characteristic	Tafenoquine (N = 46)	Chloroquine/ primaquine (N = 24)
Median age, years (range)	24 (20–43)	30 (20–55)
Male, n (%)	37 (80)	20 (83)
Asian race, n (%)	46 (100)	24 (100)
Median weight, kg (range)	52 (43–69)	54 (41–63)
Previous malaria, n (%)	30 (65)	16 (67)
Time since last attack (years), mean (SD)	0.6 (0.5)	1.4 (1.8)
Symptoms of malaria, n (%)	46 (100)	24 (100)
Body temperature (°C), mean (SD)	37 (0.85)	36.8 (0.65)
*P*. *vivax* parasite count, median parasites/μL (range)	4000 (200–44,000)	2,730 (600–30,000)
Gametocyte count* median (range)	80 (20–640)	60 (40–280)

*N = 26 for tafenoquine and N = 15 for chloroquine/primaquine.

Patients with 0 gametocytes at baseline were excluded.

### Efficacy outcomes

In the tafenoquine group, the day 28 adequate clinical response rate in the per-protocol population was 93% (40/43) (90%CI 83–98) (Fig 1). The three patients were classified per-protocol as early treatment failures on day 7 with persistent low *P*. *vivax* parasitemia at this time point (range 40–60 asexual parasites/μL). These patients were asymptomatic, cleared their parasitemia by day 8 without additional anti-malarial treatment, and remained relapse free for the duration of their follow-up (until day 28 for one patient, day 60 for the second and day 120 for the third).

**Fig 3:**
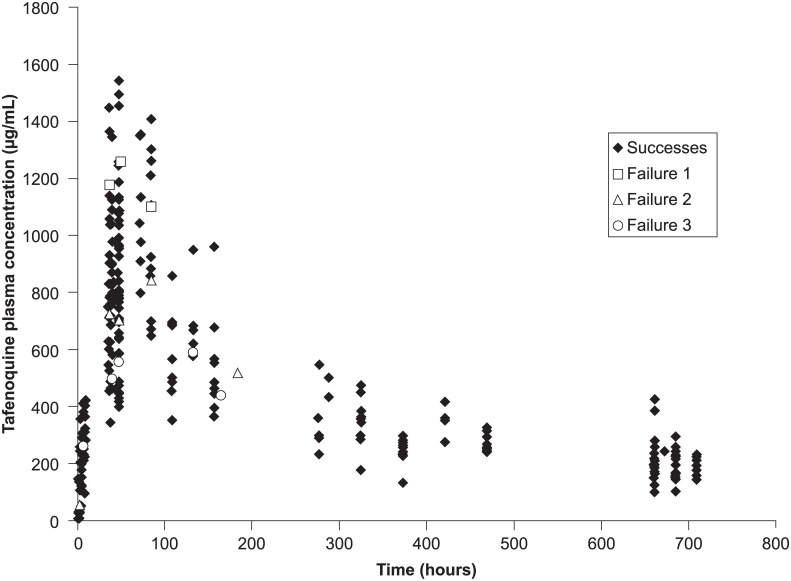
Individual patient tafenoquine plasma concentrations. *The three patients with early treatment failure (at day 7) in the tafenoquine group and slow parasite clearance had similar tafenoquine plasma concentrations to the population that had parasite clearance at day 7.

Individual patient tafenoquine concentration–time data from 46 patients are shown in Fig 3. Tafenoquine plasma concentrations were similar in the three patients with early treatment failure compared with those who achieved parasite clearance by day 7 (Fig 3). The baseline characteristics of these three patients were also similar to other patients. One was female and two were male patients, weighing 48, 53 and 57 kg, and with baseline parasitemias of 16,000, 12,000 and 22,000 parasites/μL, respectively; two had no previous episodes of malaria. Parasite, gametocyte, and fever clearance times were consistent with the slow onset of action of tafenoquine compared with those of chloroquine/primaquine (Table 2).

**Table 2:** Per-protocol population analysis of parasite, gametocyte and fever clearance time.

Treatment	Parasite clearance time, h			Gametocyte clearance time, h			Fever clearance time, h		
	N	Mean (SD)	Median (range)	N	Mean (SD)	Median (range)	N	Mean (SD)	Median (range)
Tafenoquine	41	82.5 (32.3)	84.0 (12–156)	34	49.1 (33.0)	48.0 (0–156)	31	41.1 (31.4)	36.0 (0–108)
Primaquine/ chloroquine	24	40.0 (15.7)	36.0 (24–84)	19	22.7 (16.4)	24.0 (0–60)	18	24.7 (17.7)	24.0 (0–60)

In the per-protocol population, 35 patients receiving tafenoquine and 20 patients receiving chloroquine/primaquine were evaluable through to the day 120 assessment for the prevention of *P*. *vivax* relapse. Tafenoquine prevented *P*. *vivax* relapse in all evaluable patients, while the chloroquine/primaquine arm had one patient with relapse on day 63 (Fig 1). In the intention-to-treat population, efficacy rates for relapse prevention were 88% (35/40; 90%CI 76–95) for tafenoquine and 86% (19/22; 90%CI 68–96) for chloroquine/primaquine.

### Safety outcomes

The most commonly reported adverse events are reported in Table 3. Five patients experienced a serious adverse event. All were in the tafenoquine arm and included one stabbing injury not related to study medication, and four patients with increased methemoglobinemia (protocol defined as a serious adverse event if ≥20%, n = 4) considered drug related. These four patients were all women weighing <52 kg and all remained asymptomatic. The peak methemoglobin levels occurred on day 7, ranging from 1.4% to 25.6% (median 7.4%, mean 9.1%) across all patients in the tafenoquine arm compared to 0.5% to 5.9% (median 1.5%, mean 1.9%) in the chloroquine/primaquine arm.

**Table 3:** Most common adverse events overall (regardless of causality) occurring in at least two patients in either treatment group (intention-to-treat/safety population).

Adverse event, n (%)	Tafenoquine (N = 46)	Chloroquine/ primaquine (N = 24)
ANY EVENT	46 (100.0)	22 (91.7)
Blood and lymphatic disorders		
Eosinophilia	8 (17.4)	7 (29.2)
Thrombocytopenia	6 (13.0)	0
Anemia	2 (4.3)	0
Eye disorders		
Keratopathy*	14 (31.8)	0
Retinopathy/retinal disorder*	10 (22.7)	1 (4.2)†
Conjunctivitis	0	2 (8.3)
Gastrointestinal disorders		
Abdominal pain	6 (13.0)	5 (20.8)
Nausea	6 (13.0)	3 (12.5)
Dyspepsia	3 (6.5)	1 (4.2)
Diarrhea	3 (6.5)	0
Vomiting	2 (4.3)	1 (4.2)
General disorders		
Pyrexia	5 (10.9)	3 (12.5)
Asthenia	4 (8.7)	2 (8.3)
Hepatobiliary disorders		
Hepatomegaly	3 (6.5)	0
Infections and infestations		
Upper respiratory tract infection	14 (30.4)	5 (20.8)
Subcutaneous abscess	2 (4.3)	1 (4.2)
Limb abscess	2 (4.3)	0
Intestinal parasitic infection	0	2 (8.3)
*Plasmodium falciparum* infection	0	2 (8.3)
Investigations		
Methemoglobinemia (≥8.5%)	22 (47.8)	0
Eosinophil count increased	5 (10.9)	3 (12.5)
Abnormal hepatic enzymes	2 (4.3)	0
Metabolism and nutrition disorders		
Hypokalemia	3 (6.5)	1 (4.2)
Musculoskeletal/connective tissue disorders		
Myalgia	3 (6.5)	1 (4.2)
Nervous system disorders		
Headache	14 (30.4)	4 (16.7)
Dizziness	12 (26.1)	3 (12.5)
Respiratory, thoracic and mediastinal disorders		
Nasal congestion	2 (4.3)	0
Skin and subcutaneous tissue disorders		
Eczema	2 (4.3)	1 (4.2)

*Note that 44/46 patients had a post-baseline eye assessment in the tafenoquine group. Eye examination was performed at baseline and days 28 and 90.

^†^One additional patient receiving chloroquine had bilateral baseline retinal hemorrhage which considered pre-existing and not noted as an adverse event.

Renal safety was assessed with serial serum creatinine and urinalysis assessments. One patient receiving tafenoquine had a change from baseline in serum creatinine of 0.98 μmol/L, which resolved spontaneously. Otherwise, routine urinalysis parameters were comparable between the two treatment arms.

## Discussion

This small phase II study demonstrated that a three-day course of tafenoquine monotherapy was efficacious for the radical cure of *P*. *vivax* malaria, clearing parasite blood stages by day 7 in 93% of patients and preventing relapse in all evaluable patients for at least four months. However, 1200 mg tafenoquine over three days had slower resolution of symptoms and clearance of blood-stage malaria parasites (7% early treatment failure per-protocol) compared with chloroquine/primaquine. Also, a parasite clearance rate of 82 h for tafenoquine would provide a long window for the selection of resistance [24]. Current thinking is that monotherapy in malaria is undesirable, given the risk of parasite resistance selection, particularly with a long half-life, slow acting drug such as tafenoquine [24, 25]. This study was conducted as one of a number of investigations examining tafenoquine dosing and efficacy in *P*. *vivax* malaria treatment and prophylaxis. These studies eventually led to the formulation of the current clinical development plan for tafenoquine plus chloroquine or an ACT in *P*. *vivax* malaria radical cure. Recently a phase IIb study resulted in the selection of a dosing regimen comprising a single 300 mg tafenoquine dose plus standard 3-day chloroquine therapy—at this dose, 6-month anti-recurrence efficacy was 89.2% (95%CI 77–95%; n = 57) compared with 77.3% (95%CI 63–87%; n = 50) with chloroquine plus 15 mg x 14 day primaquine, and 37.5% (95%CI 23–52%; n = 54) with chloroquine alone [8]. The single-dose tafenoquine 300 mg plus standard 3-day chloroquine regimen is now being tested in a phase III trial, with a further phase III trial planned for single-dose tafenoquine plus ACTs (Clinicaltrials.gov: NCT01376167 and NCT02802501).

Although there were no clinical symptoms, the 1200 mg total dose given over three days in the current study increased methemoglobin levels in the tafenoquine arm to an extent that would be concerning if used in a less closely monitored population. With tafenoquine doses up to 600 mg, no adverse events have been reported for methemoglobinemia [8, 14, 15]. Note that G6PD-deficient subjects were excluded from the current study [20].

A previous study of tafenoquine prophylaxis [22], reported renal safety signals (increased creatinine) and eye adverse events. There was no indication of any renal safety concerns in the current study, consistent with other published studies [8, 23]. In the prophylaxis study, there was mild vortex keratopathy observed in 69/74 (93.2%) subjects and retinal abnormalities noted on clinical examination in 27/69 (39.1%) [22]. The changes were reversible and resolved by 1 year and did not adversely affect visual acuity, and an Independent Expert Ophthalmology Review Board concluded that the retinal findings could have been normal variations with no evidence of drug-related visual disturbances [22]. In the current study with 1200 mg tafenoquine, detailed prospective eye assessment, both clinical and using digital photography, at baseline and post-baseline are reported separately. Briefly, though of no concern in terms of functional impairment, possible early retinal morphological changes related to tafenoquine cannot be ruled out in one case. With tafenoquine doses up to 600 mg there were no reports of keratopathy in patients receiving tafenoquine, though 7/61 (11%) had post-baseline transient changes in the results of their Humphrey visual field test, all of which resolved by day 180 [8].

In conclusion, this study adds to the published data on tafenoquine treatment of *P*. *vivax* malaria, showing effective, though slow, blood schizonticidal activity and providing high-dose safety data.

## Supporting information

S1 CONSORT Checklist CONSORT 2010 checklist Fukuda et al. (DOC)

S1 Protocol GSK-252263-058-protocol-redact. (PDF)
