# Supplementary material for: A randomized, double-blind, active-control trial to evaluate the efficacy and safety of a three day course of tafenoquine monotherapy for the treatment of Plasmodium vivax malaria
Source: PLoS One. 2017 Nov 9;12(11):e0187376. doi: 10.1371/journal.pone.0187376 (PMC5679603; doi:10.1371/journal.pone.0187376)
Supplement: S1 Protocol — (PDF) [file pone.0187376.s002.pdf]

Title:

A randomized, active-control, double-blind, double-dummy study to evaluate the efficacy and safety of Tafenoquine for the treatment of *Plasmodium vivax* in adults

Document Number: UM2002/00044/03
Study Identifier: SB-252263/058
GSK Compound Number: SB-252263
Issue Date: 18 Dec 2003
Protocol Amendment Number: 03

Author(s):

GlaxoSmithKline

[redacted] Anti-Infectives CDMA North America

[redacted] Biomedical Data Sciences

[redacted] Clinical Pharmacology and Discovery Medicine

[redacted]

[redacted] MAJ, MC Department of Immunology and Medicine

[redacted] LTC, MC Department of Immunology and Medicine

Walter Reed Army Institute of Research

[redacted] LTC, MC Department of Pharmacology Division of Experimental Therapeutics

Revision Chronology:

UM2002/00044/00	2003-MAR-12	Original
UM2002/00044/01	2003-JUL-16	Amendment 01: Changes made for clarification and for corrections to the original procotol.

(Continues)

Copyright 2003 the GlaxoSmithKline group of companies. All rights reserved. Unauthorised copying or use of this information is prohibited.

Revision Chronology (Continued):

UM2002/00044/02	2003-AUG-18	Amendment 02: Change in Section 6.3.1 Primary Efficacy to clarify methods for calculating parasite density.
UM2002/00044/03	2003-DEC-18	Amendment 03: Changes in Sections 4.1 General Study Design and 5 Study Population expanding recruitment sites, and 11.3.1 Interim Analysis and 12.8 Independent Data Monitoring Committee detailing changes in the planned interim analysis

CONFIDENTIAL UM2002/00044/03
The GlaxoSmithKline group of companies SB-252263/058

**GSK Signatory:**	**Signature:**	**Date:**
[redacted]<br>VP, Anti-infectives, Clinical Development and Medical Affairs North America	[redacted]	13 Dec 2003

SPONSOR INFORMATION PAGE

Title:	A randomized, active-control, double-blind, double-dummy study to evaluate the efficacy and safety of Tafenoquine for the treatment of *Plasmodium vivax* in adults
Study Identifier:	SB-252263/058

Sponsor for tafenoquine, IND #38,503, is the Office of the Surgeon General, Department of the Army. Tafenoquine is being developed by the U.S. Army Medical Research and Materiel Command (USAMRMC) in collaboration with its co-development partner GlaxoSmithKline.

Sponsor:
Department of Defense
Office of the Surgeon General, Department of the Army

Point Of Contact, Coordinating Office:
U.S. Army Medical Research and Material Command
Office of Regulatory Compliance and Quality
504 Scott Street
Fort Detrick, MD 21702-5012
[redacted]

Sponsor Representative :
[redacted] Ph.D.
U.S. Army Medical Materiel Development Activity
622 Neiman Street
Fort Detrick, MD 21702-5012
[redacted]

GSK Representative:
GlaxoSmithKline
1250 South Collegeville Road
Collegeville, PA 19426, USA
Telephone Number: [redacted]

Contact Information:
Dr. [redacted]
GlaxoSmithKline
Mail Code UP4215
1250 S. Collegeville Road
Collegeville, PA 19426
Phone: [redacted] Fax: [redacted]

IND Number: 38,503

INVESTIGATOR AGREEMENT PAGE

I agree:

- To assume responsibility for the proper conduct of the study at this site.
- To conduct the study in compliance with this protocol amendment and with any other study conduct procedures provided by USAMRMC and GlaxoSmithKline (GSK).
- Not to implement this protocol amendment without agreement from USAMRMC and GSK and prior submission to and written approval from (where required) the Institutional Review Board (IRB) or Independent Ethics Committee (IEC), except when necessary to eliminate an immediate hazard to the subjects, or for administrative aspects of the study (where permitted by all applicable regulatory requirements).
- Not to implement any other changes to the protocol without agreement from USAMRMC and GSK and prior review and written approval from the IRB or IEC, except where necessary to eliminate an immediate hazard to the subjects, or for administrative aspects of the study (where permitted by all applicable regulatory requirements).
- That I am thoroughly familiar with the appropriate use of the investigational product(s), as described herein, and any other information provided by the sponsor including, but not limited to, the following: the current Clinical Investigator’s Brochure (CIB) or equivalent document, CIB supplement (if applicable), and approved product label (if the product is marketed in this country and the label is not already provided as an equivalent to a CIB).
- That I am aware of, and will comply with, “good clinical practices” (GCP) and all applicable regulatory requirements.
- To ensure that all persons assisting me with the study are adequately informed about the investigational product(s) and of their study-related duties and functions as described herein.
- That I have been informed that certain regulatory authorities require USAMRMC and GSK to obtain and supply, as necessary, details about the investigator’s ownership interest in GSK or the investigational product, and more generally about his/her financial ties with the GSK. USAMRMC and GSK will use and disclose the information solely for the purpose of complying with regulatory requirements.

  Hence I:

  - Agree to supply USAMRMC and GSK with any necessary information regarding ownership interest and financial ties (including those of my spouse and dependent children);
  - Agree to promptly update this information if any relevant changes occur during the course of the study and for 1 year following completion of the study; and

- Agree that USAMRMC and GSK may disclose any information it has about such ownership interests and financial ties to regulatory authorities.

Investigator Name: ______________________________

____ ____________________
Investigator Signature Date

Table of Contents

Page

ABBREVIATIONS 11

PROTOCOL SUMMARY 12

1. INTRODUCTION 20
    1.1. Background 20
    1.2. Rationale 25

2. OBJECTIVE(S) 26
    2.1. Primary 26
    2.2. Secondary 26

3. ENDPOINT(S) 26
    3.1. Primary 26
    3.2. Secondary 27

4. STUDY DESIGN 27
    4.1. General Study Design 27
    4.2. Risks and Benefits to Research Subjects 29
        4.2.1. Risks to Research Subjects 29
        4.2.2. Benefits to Research Subjects 30

5. STUDY POPULATION 30
    5.1. Number of Subjects 31
    5.2. Eligibility Criteria 31
        5.2.1. Inclusion Criteria 31
        5.2.2. Exclusion Criteria 32
        5.2.3. Other Eligibility Criteria Considerations 33

6. STUDY ASSESSMENTS AND PROCEDURES 33
    6.1. Demographic and Baseline Assessments 33
    6.2. Safety 34
        6.2.1. Vital Signs 34
        6.2.2. Clinical Chemistry, Methemoglobin and Hematology 34
        6.2.3. Renal Assessments 34
        6.2.4. Urinalysis 35
        6.2.5. Ophthalmic Assessments 35
        6.2.6. Pregnancy 36
    6.3. Efficacy 37

6.3.1. Primary Efficacy 37
6.3.2. Secondary Efficacy Measures 38
6.3.3. Efficacy Monitoring During Enrollment 38
6.4. Pharmacokinetics 39
6.4.1. Sample Collection 39
6.4.2. Processing of Samples 40
6.5. Other Studies 40

7. INVESTIGATIONAL PRODUCT(S) 40
7.1. Description of Investigational Product 40
7.2. Dosage and Administration 42
7.3. Dose Rationale 42
7.4. Blinding 43
7.5. Treatment Assignment 44
7.6. Packaging and Labeling 44
7.7. Handling and Storage 44
7.8. Product Accountability 45
7.9. Assessment of Compliance 45
7.10. Treatment of Investigational Product Overdose 45
7.11. Occupational Safety 45

8. CONCOMITANT MEDICATIONS AND NON-DRUG THERAPIES 46
8.1. Permitted Medications 46
8.2. Prohibited Medications 46

9. SUBJECT COMPLETION AND WITHDRAWAL 46
9.1. Subject Completion 46
9.2. Subject Withdrawal 47
9.2.1. Subject Withdrawal from Study 47
9.2.2. Subject Withdrawal from Investigational Product 48

10. ADVERSE EVENTS (AE) AND SERIOUS ADVERSE EVENTS (SAE) . . 48
10.1. Definition of an AE 48
10.2. Definition of a SAE 49
10.3. Lack of Efficacy 50
10.4. Clinical Laboratory Abnormalities and Other Abnormal Assessments as AEs and SAEs 50
10.5. Time Period, Frequency, and Method of Detecting AEs and SAEs . 51
10.6. Recording of AEs and SAEs 52
10.7. Evaluating AEs and SAEs 53

10.7.1. Assessment of Intensity 53
10.7.2. Assessment of Causality 53
10.8. Follow-Up of AEs and SAEs 54
10.9. Prompt Reporting of SAEs to USAMRMC and GSK 54
10.9.1. Timeframes for Submitting SAE Reports to USAMRMC and GSK 55
10.9.2. Completion and Transmission of the SAE Reports 55
10.10. Regulatory Reporting Requirements For SAEs 56
10.11. Post-study AEs and SAEs 57
10.12. SAEs Related to Study Participation 57

11. DATA ANALYSIS AND STATISTICAL CONSIDERATIONS 57
11.1. Hypotheses 57
11.2. Treatment Comparisons of Interest 57
11.2.1. Primary Comparisons of Interest 57
11.2.2. Other Comparisons of Interest 58
11.3. Interim Analysis 58
11.3.1. Planned Interim Analysis After Cohort 1 Day 28 Assessment 58
11.3.2. Ad-hoc Interim Analyses During Enrollment 58
11.4. Sample Size Considerations 60
11.4.1. Sample Size Assumptions 60
11.4.2. Sample Size Sensitivity 61
11.4.3. Sample Size Re-estimation 62
11.5. Analysis Populations 62
11.5.1. Data Sets 63
11.6. General Considerations for Data Analysis 64
11.6.1. Withdrawal 64
11.6.2. Missing Data 64
11.6.3. Derived and Transformed Data 64
11.6.4. Assessment Windows 65
11.6.5. Other Issues 65
11.7. Efficacy Analyses 65
11.7.1. Primary Analysis 65
11.7.2. Other Analyses 66
11.7.3. Robustness Analyses 67
11.8. Safety Analyses 67
11.8.1. Extent of Exposure 67

11.8.2. Adverse Events 67
11.8.3. Clinical Laboratory Evaluations 68
11.8.4. Renal Data Analyses 68
11.8.5. Ophthalmic Data Analyses 68
11.8.6. Methemoglobin, Vomiting, Diarrhea and Nausea AEs 68
11.9. Clinical Pharmacology Data Analyses 69
11.9.1. Pharmacokinetic Analyses 69

12. STUDY ADMINISTRATION 69
12.1. Regulatory and Ethical Considerations 69
12.1.1. Regulatory Authority Approval 69
12.1.2. Ethical Conduct of the Study and Ethics Approval 69
12.1.3. Informed Consent 70
12.1.4. Investigator Reporting Requirements 70
12.2. Medical Monitor 70
12.3. Study Monitoring 71
12.4. Quality Assurance 72
12.5. Study and Site Closure 72
12.6. Records Retention 73
12.7. Provision of Study Results and Information to Investigators 73
12.8. Information Disclosure and Inventions 74
12.9. Data Management 75
12.10. Independent Data Monitoring Committee (IDMC) 75

13. REFERENCES 77

14. APPENDICES 78
14.1. Appendix 1: Time and Events Table 79
14.2. Appendix 2: Ophthalmic Assessment Details 83
14.3. Appendix 3: Organization Of Research Effort 88
14.4. Appendix 4: Research Facilities and IEC/IRBs 91
14.5. Appendix 5: Protocol Changes 92

ABBREVIATIONS

ACR	Adequate Clinical Response
AE	Adverse Event
ALT	Alanine Transaminase
AST	Aspartate Transaminase
BCVA	Best Corrected Visual Acuity
[redacted]	[redacted]
BUN	Blood Urea Nitrogen
CDMA	Clinical Development And Medical Affairs
CIB	Clinical Investigator's Brochure
CL/F	Oral Clearance
CRF	Case Report Form
DMID	Division of Microbiology and Infectious Diseases
EISR	Expedited Investigator Safety Report
ETF	Early Treatment Failure
FCT	Fever Clearance Time
FDA	Food And Drug Administration
G6PD	Glucose-6-Phosphate Dehydrogenase
GCP	Good Clinical Practice
GCSP	Global Clinical Safety And Pharmacovigilance
GCT	Gametocyte Clearance Time
GGT	Gamma Glutamyl Transferase
GSK	GlaxoSmithKline
HCVA	High Contrast Visual Acuity
IDMC	Independent Data Monitoring Committee
IEC	Independent Ethics Committee
IND	Investigational New Drug
IRB	Institutional Review Board
ITT	Intent To Treat
ka	Rate of Constant Absorption
LTF	Late Treatment Failure
MAR	Minimum Angle Of Resolution
MSDS	Material Safety Data Sheet
PCT	Parasite Clearance Time
PP	Per Protocol
RAMOS	Registration and Medication Ordering System
RAP	Reporting and Analysis Plan
SAE	Serious Adverse Event
SD	Standard Deviation
SDAC	Statistical Data and Analysis Center
USAMRMC	United States Army Medical Research and Materiel Command
V/F	Oral Volume of Distribution
WBC	White Blood Cell Count

PROTOCOL SUMMARY

Rationale

To prevent transmission of malaria, a drug, which eradicates both the asexual and sexual stages in the blood, and protects from further infection (prophylaxis) for longer than the 2-3 week incubation time in mosquitoes, would be needed. To prevent transmission of *P. vivax*, a drug which also eradicates the dormant liver stage (hypnozoites) would be needed. No currently available drug acts at all stages of the lifecycle of the plasmodium. Some, such as chloroquine are blood schizontocides and act quickly to kill parasites in the red blood cells. Others, such as primaquine have gametocytocidal activity, killing blood sexual forms thereby preventing transmission to the mosquito. Causal prophylactics such as atovaquone and primaquine, kill the intrahepatic stages of the developing schizonts and prevent the need for prophylaxis after leaving an endemic area. However, only primaquine is able to eradicate the hypnozoites of *P. vivax*.

Tafenoquine has been used very successfully as a prophylactic agent against both *P. vivax* and *P. falciparum* and has proven effective against *P. vivax* malaria hypnozoites (dormant liver stages) in small trials. With its long half-life and efficacy, tafenoquine has great promise as an agent to interrupt the transmission of malaria in large populations with limited repeat dosing.

Although developed primarily as an anti-relapse agent, tafenoquine has also been found to possess significant blood schizonticidal activity. Tafenoquine cured established blood-induced infections of the Chesson strain of *vivax* malaria in *Aotus* monkeys. Clearance was obtained at 3 mg/kg and cures were achieved at 9 mg/kg total dose administered in a 3-day oral regimen [Obaldia, 1997]. In rhesus monkeys, a dose of 3.16 mg/kg x 7 days was found to be fully curative against blood stage infections of either *P. cynomolgi* or *P. fragile*. [Puri, 2003].

However, activity for clearing blood stages of *P. falciparum* and *P. vivax* in humans has not yet been demonstrated, nor has gametocytocidal activity been demonstrated *in vivo*. Furthermore, the optimum dose of this therapy for potentially blocking transmission is not yet known. Doses of tafenoquine of 200 mg are typically effective for prophylaxis. However, it is anticipated that higher doses of tafenoquine will be required to clear parasitemia, since other aminoquinolones (chloroquine and mefloquine), also require higher doses for treatment of malaria than are needed for prophylaxis. Based on efficacy, safety, tolerability and pharmacokinetic data gathered during Phase I and II, doses of 600 mg for one day, and 400 mg for three days have been selected as the doses to evaluate tafenoquine as a single agent for the radical cure of *P. vivax*. The 400 mg dose for 3 days was better tolerated than repeat regimens at higher doses (500 mg or 600 mg for 3 days), and has demonstrated efficacy for eradication of *P. vivax* hypnozoites. The 600 mg dose for one day, is the maximum single dose of tafenoquine administered to subjects and has also demonstrated efficacy for eradication of *P. vivax* hypnozoites.

In both Phase I and Phase II studies conducted with tafenoquine to date, single and repeat dosing of levels up to 600mg have been generally well tolerated although there is evidence of increased methemoglobin concentration and gastrointestinal disturbances (diarrhea, vomiting, and nausea) with increasing dose. The development of corneal deposits has been observed following chronic (6 months) dosing with tafenoquine. However, the corneal changes were benign, fully reversible and similar to those seen with other drugs such as chloroquine. Transient increases in serum creatinine have also been observed with tafenoquine. All data to date suggest it is mild and reversible. Although the existing data cannot exclude the occurrence of renal damage, this is considered unlikely.

This Phase II study is designed to determine whether a single 600 mg dose or 400mg/day for 3 days of tafenoquine is efficacious, and well tolerated for clearing *P. vivax* malaria infection (blood schizontocidal and gametocytocidal activity) and preventing *P. vivax* relapse (hypnozoite eradication). It will also further establish the safety and tolerability of these doses of tafenoquine.

As the activity of tafenoquine for clearing blood stages of *P. vivax* has not yet been demonstrated, this study will be conducted in 2 cohorts. Cohort 2 (600mg tafenoquine for 1 day) will not be progressed if insufficient efficacy or safety findings of concern are observed in Cohort 1 (400mg tafenoquine for 3 days).

Enrollment will be suspended in the unlikely event that pre-set criteria for lack of efficacy, renal or ophthalmic findings, or adverse events of severe intensity, including vomiting, diarrhea, or nausea, are met. An Independent Data Monitoring Committee will be used to assist evaluation of relevant data and advise on resuming enrollment of subjects.

Objective(s)

Primary: To assess whether a single 600 mg dose or 400mg/day for 3 days of tafenoquine alone can clear/cure *P. vivax* blood stage infections.

Secondary: To assess whether a single 600 mg dose or 400mg/day for 3 days of tafenoquine alone can prevent *P. vivax* relapse for 2, 3 and 4 months.

Determination of Parasite, Gametocyte and Fever Clearance Time (PCT, GCT, and FCT).

To establish the safety and tolerability of these doses of tafenoquine.

To characterize the population pharmacokinetics of tafenoquine in a malaria-infected population.

Endpoint(s)

Primary: The primary efficacy endpoint will be:

- Day 28 cure rate. A subject will be considered a success (cure) if they have an Adequate Clinical Response (ACR).

  Adequate Clinical Response

  - The subject does not develop any of the conditions of Early Treatment Failure (ETF) or Late Treatment Failure (LTF).
  - -Parasitological clearance has been confirmed throughout the follow-up period (Day 28).

- A subject will be considered a failure if they meet the definition of Early Treatment Failure (ETF) or Late Treatment Failure (LTF) as defined below.

  Early Treatment Failure

  - Parasitemia on Day 7

  Late Treatment Failure

  - Parasitemia recurring after Day 7 up to and including Day 28 (same species as on Day 0).

Subjects who are treatment failures will be withdrawn from the study and given appropriate anti-malarial treatment according to [redacted] guidelines. (All randomized subjects who received at least one dose of study medication (i.e. tafenoquine or chloroquine/primaquine) will be included in the Safety and Intent to Treat (ITT) analysis populations.)

Secondary Endpoints:

Efficacy and Safety:

- **Proportion of subjects without relapse of *P. vivax* at 2, 3 and 4 months.**
- **Determination of Parasite, Gametocyte, and Fever Clearance Time (PCT, GCT, and FCT).**
- **Safety: To further establish the safety and tolerability of the tafenoquine dosing regimens.**

Pharmacology:

- **Population pharmacokinetic parameters (CL/F, V/F, ka) will be estimated for tafenoquine.**

Study Design

This will be a randomized, active-control, double-blind, double-dummy study, conducted in 2 sequential cohorts in a total of 140 subjects in order to obtain 120 evaluable (60/cohort) male and female subjects age 20-60 years with confirmed *P. vivax* malaria. Subjects enrolled into the study will be hospitalized at the [redacted] for the first 29 days of the study and will be asked to remain in a malaria free region until 90 days after the start of the study for follow up with scheduled assessments at Days 60 and 90. Subjects will be contacted at Day 120 for a follow-up blood smear. Subjects will be given tafenoquine alone or the standard regimen of chloroquine followed by primaquine (2:1 ratio, 40:20 subjects in each cohort). Subjects will provide written informed consent and will undergo screening and baseline procedures before the start of the study. Subjects in Cohort 1 will be randomized to receive either tafenoquine (400mg base) and chloroquine placebo for 3 days, followed by primaquine placebo for 14 days, or chloroquine (1000 mg chloroquine phosphate) and tafenoquine placebo for 2 days, followed by chloroquine (500 mg chloroquine phosphate) and tafenoquine placebo for 1 day, followed by primaquine, 15 mg/day for 14 days.

The efficacy (Day 28 cure rate) of the dosing regimen in Cohort 1 will be compared to prespecified criteria (as detailed in Section 11.3) once all subjects have completed the 90 day assessment. These efficacy results and the safety profile will be examined before enrolling Cohort 2. Subjects in Cohort 2 will be randomized to receive either tafenoquine (600 mg base) and chloroquine placebo for 1 day, followed by chloroquine placebo for 2 days, followed by primaquine placebo for 14 days, or chloroquine (1000 mg chloroquine phosphate) and tafenoquine placebo for 1 day, followed by chloroquine (1000 mg chloroquine phosphate) for 1 day, followed by chloroquine (500 mg chloroquine phosphate) for 1 day, followed by primaquine, 15 mg/day for 14 days.

Subjects will be involved in the study for 121 days from start of treatment to end of routine follow-up. Further follow-up will occur for any renal or ophthalmic findings at the discretion of the investigator.

Study Population

A total of approximately 140 male and female subjects between the ages of 20 and 60 years inclusive will be recruited for this study in order to obtain 40 evaluable subjects in each of the 2 cohorts for the tafenoquine arm and 20 evaluable subjects in each of the 2 cohorts for the comparator arm. Subjects presenting to [redacted] health care system with microscopy proven *Plasmodium vivax* malaria will be offered enrollment. They will be hospitalized for the first 29 days of the study for close monitoring and asked to remain where malaria is not endemic until 90 days after the start of the study, to reduce the chance of re-infection. Participants will undergo routine labs (including G6PD) and other baseline studies prior to receiving the first dose

of study drug. They must be willing to give informed consent and able to comply with the procedures of the protocol.

Study Assessments and Procedures

At the screening/baseline visit the study will be fully explained to the subjects and written informed consent obtained. Subjects will be assessed as to whether they meet the inclusion and exclusion criteria. Demographic details (age, sex, weight, height) and medical history and concomitant medication details will be recorded. Vital signs (pulse, blood pressure, respiratory rate, and temperature) will be recorded and a physical exam will be performed. A blood smear to confirm *Plasmodium vivax* malaria will be performed. A blood sample will be taken for biochemistry, methemoglobin, and hematology analysis and a urine sample provided for urinalysis. All subjects will have their G6PD status tested at baseline. All female subjects will undergo a pregnancy test (urine or serum); pregnant subjects may not enter the study.

Baseline eye examinations (visual acuity, amsler grid, color vision, corneal and retinal examination/photography) will be performed prior to first dose of tafenoquine or comparator medication if possible. If the scheduling of the full eye exam will significantly delay the start of treatment (> 10 hours) or if the subject is deemed by the investigator to be too ill to undergo the complete eye examination, a basic eye exam to assess vision problems will be performed prior to dosing. The full eye exam will then be conducted as soon as possible but no later than within 36 hours of receiving the first dose of study drug.

Clinical Chemistry, Methemoglobin, and Hematology

A blood sample will be taken for biochemistry analysis at baseline and at Days 3, 7, 14, 21, 28 and 90. For biochemistry the following parameters will be assessed: blood urea nitrogen (BUN), creatinine, glucose, sodium, potassium, chloride, total carbon dioxide, calcium, aspartate aminotransferase (AST), alanine aminotransferase (ALT), gamma-glutamyl transferase (GGT), alkaline phosphatase, total bilirubin, albumin, and total protein. A blood sample will be taken for hematology analysis at baseline and at Days 3, 7, and 14. For hematology the following parameters will be assessed: complete blood count, including red cell indices and white cell differential, and platelet count. A blood sample will be taken to determine methemoglobin concentration at baseline and Days 3, 7, and 21.

Urinalysis

A "clean-catch" (midstream) urine sample will be collected at baseline and a morning urine sample will be collected at the Day 7 and Day 28 visits and the morning after a sustained (≥ 14 days) serum creatinine increase of ≥26.6 μmol/L (0.3 mg/dL) from baseline is confirmed. The time of urine sample collection will be recorded and the following parameters will be assessed: specific gravity and/or urine osmolality, pH, glucose, protein, blood and ketones by dipstick. If urine dipstick analyses are abnormal, a urine microscopic examination will be performed. Abnormal urine dipsticks will be

repeated every week until normalized or hospital discharge. All urine samples will be stored at -20°C or colder for possible measurement of urine protein:creatinine ratio.

Efficacy Assessments

Thick and thin blood smears for malaria will be obtained by finger prick at baseline (Day 0) and then every 12 hours (± 2 hours) up to and including Day 7, until the blood smear becomes negative. Parasites and gametocytes will be considered cleared if 2 consecutive blood smears are negative. Once confirmed to be negative, blood smears will be conducted once a day up to and including Day 7. For both Cohort 1 and Cohort 2, if ≥4 Early Treatment Failures (ETFs) occur among the first 21 subjects enrolled into the Cohort, further enrollment of subjects will be suspended, and the data will be sent to an independent Statistical Data Analysis Center (SDAC) for treatment unblinding and analysis. If based on these data, the SDAC confirms that the actual response rate among evaluable tafenoquine subjects is ≥70%, enrollment will be re-initiated. If based on these data, the SDAC confirms that the actual response rate among evaluable tafenoquine subjects is below 70%, enrollment will remain suspended and the SDAC will be required to call an ad-hoc Independent Data Monitoring Committee (IDMC) meeting. The IDMC will be requested to review all relevant data and to provide guidance as to how to proceed, which may include recommending continuation of the study, scheduling a second interim look, a change to the protocol, or stopping of the study.

Renal Assessments

Serum creatinine will be measured as part of the biochemistry analysis at baseline (Day 0), Day 3, Day 7, Day 14, Day 21, Day 28, and Day 90. If serum creatinine is increased ≥26.6 µmol/L (0.3 mg/dL) compared to baseline, it will be repeated within 24 hours. Subjects with confirmed serum creatinine increases of ≥26.6 µmol/L (0.3 mg/dL) will be reassessed 7 and 14 days after the initial increase of ≥26.6 µmol/L (0.3 mg/dL), or more frequently at the discretion of the investigator, to determine if the serum creatinine level is returning to baseline. If a sustained (≥ 14 days) serum creatinine increase of ≥26.6 µmol/L (0.3 mg/dL) is confirmed it will be upgraded to GSK medically defined serious for fast-tracking on to the safety database and for rapid follow-up. A morning "clean-catch" (midstream) urine sample will be collected on the following day for urinalysis and will be stored at -20°C or colder for possible measurement of urine protein:creatinine ratio. If a sustained (≥ 14 days) serum creatinine increase of ≥26.6 µmol/L (0.3 mg/dL) is confirmed in ≥8 subjects in total within a cohort, enrollment of further subjects will be put on hold until the data can be reviewed by an IDMC for recommendation on continued enrollment of subjects. Follow up of subjects with raised creatinine will continue to assess the reversibility of any findings.

Ophthalmic Assessments

Ophthalmic safety will be assessed in all subjects using the following tests at baseline and at Day 28 and Day 90 visits.

- Macular Function Tests:

  Amsler Grid, Humphrey 10-2 Visual Field, Macular stress test, High contrast visual acuity (HCVA) – ETDRS chart, Color vision – PIP plates & Lanthony 40 hue

- Digital Photography:

  Corneal and retinal digital photographs

If at any timepoint, retinal changes from baseline are seen that cause clinical concern to the study ophthalmologist (as detailed in Section 6.2.5, these would include decreased vision, bull's eye retinopathy, distortions observed on the Amsler Grid test, abnormal color vision or development of a scotoma on visual field testing), it will be upgraded to GSK medically defined serious for fast-tracking on to the safety database and for rapid follow-up. If retinal changes from baseline that cause clinical concern are seen in ≥2 subjects in total within a cohort, enrollment of further subjects will be put on hold until the data can be reviewed by an IDMC for recommendation on continued enrollment of subjects. Subjects will be followed up to assess the reversibility of any findings.

Adverse Event Assessments

At each clinic visit, details of any adverse events experienced since the last visit, will be recorded. Due to the trend with tafenoquine of increased methemoglobin concentration and gastrointestinal disturbances (diarrhea, vomiting, and nausea) with increasing dose, adverse events of significantly increased methemoglobin concentration, vomiting, diarrhea, and nausea that are assessed by the investigator as severe, will be upgraded to GSK medically defined serious for fast-tracking on to the safety database and for rapid follow-up. If ≥5 subjects within a cohort have the following same drug related AE (based on body systems) of severe intensity:

- Methemoglobin ≥ 20%
- Recurrent (> 4 times within one day) or intractable vomiting
- Severe diarrhea resulting in loss of intravascular volume judged by the investigator to be sufficiently severe to warrant the initiation or increase in rate of administration of intravenous replacement fluids after onset of diarrhea
- Nausea resulting in no significant oral intake, requiring intravenous fluids

enrollment of further subjects will be put on hold until the data can be reviewed by an IDMC for recommendation on whether to continue enrolling into the study.

Population Pharmacokinetics

Sample Collection:

Blood samples will be collected from all subjects for population pharmacokinetic analysis at the following designated times:

i. One sample between 1 and 10 hours post-first dose

ii. One sample between 36 and 42 h post first dose (12-18 h post second dose)

iii. One sample 48 h post first-dose (pre-third dose for 400 mg x 3 d)

iv. One sample between 72 and 168 h post first dose (Day 3- Day 7)

v. Day 12-20

vi. Day 28-30

vii. At recurrence of *P. vivax* after Day 7 up to and including Day 28 if a PK sample has not been drawn within the previous 24 hours

Sampling times within a particular window should be spread across that window among subjects rather than being grouped at extreme ends of the window (eg. 1-10 h window – not all samples taken at 1 h or 10 h). Working instructions will be developed and followed to insure that sampling times will be adequately bracketed within the sampling window.

Investigational Product(s)

Subjects will be randomized to one of two treatment groups in a 2:1 (tafenoquine:chloroquine/primaquine) ratio.

Subjects within Cohort 1 will be randomized to one of the two treatment groups:

1. Tafenoquine (400mg base) and chloroquine placebo x 3d , followed by primaquine placebo for 14 days.
2. Chloroquine (1000 mg chloroquine phosphate) and tafenoquine placebo x 2 day, followed by chloroquine (500 mg chloroquine phosphate) and tafenoquine placebo x 1day, followed by primaquine, 15 mg/day for 14 days.

The efficacy and safety of the dosing regimen in Cohort 1 through Day 90 will be compared to prespecified criteria (see Section 11.3) before enrolling Cohort 2.

Subjects within Cohort 2 will be randomized to one of the two treatment groups:

1. Tafenoquine (600 mg base) and chloroquine placebo x 1d, chloroquine placebo x 2 days, followed by primaquine placebo for 14 days.
2. Chloroquine (1000 mg chloroquine phosphate) and tafenoquine placebo x 1 day, followed by chloroquine (1000 mg chloroquine phosphate) x 1 day, followed by chloroquine (500 mg chloroquine phosphate) x 1day, followed by primaquine, 15 mg/day for 14 days.

1. INTRODUCTION

1.1. Background

Malaria is a leading cause of morbidity and mortality in many developing countries with an estimated 300 to 500 million clinical cases worldwide every year and between 1 and 3 million deaths, mostly of children, are attributable to this disease [Sachs, 2002].

The sporozoite form of the Plasmodium parasite is transmitted through the saliva of Anopheles mosquitoes as they feed on human blood during bites. Within minutes after inoculation, the sporozoites travel through the blood and invade the liver where they undergo asexual division and mature. The pre-patent period (the time between the mosquito bite and the first appearance of plasmodia in the peripheral blood) for the Plasmodium parasite in man normally ranges between 9-12 days. The shortest reported pre-patent period in man is 5 days while the longest (in individuals not taking an antimalarial drug) is 25 days. From the liver, merozoites are released into the blood and invade the erythrocytes, where they develop into schizonts. In the case of *P. falciparum* malaria, there are no residual parasites in the liver after the initial cycle of entry, division, maturation and release. However, a proportion of *P. vivax* sporozoites develop into a dormant state in the liver, called hypnozoites. These latent sporozoites periodically re-enter the development cycle causing clinical relapses. Drugs with activity versus the exoerythrocytic stage (i.e., the hepatic stage) of the parasite's life cycle are known as causal prophylactic drugs. They effectively disrupt the life-cycle of the parasites, preventing parasitemia, systemic illness and further transmission. Drugs active on the erythrocytic schizonts are called blood schizontocides. These drugs are used to treat clinically apparent malaria infections, or as a suppressive prophylactic drug, which prevents clinical symptoms by destroying schizonts before they can cause illness.

There are currently no effective vaccines to prevent malaria infections. For non-immune individuals, drugs have been used over the years to prevent malaria. Although drugs such as chloroquine, pyrimethamine-sulfadoxine, mefloquine, and doxycycline have been used successfully in the past to prevent malaria infections, the advent of chloroquine-resistant strains of *P. falciparum* and *P. vivax* malaria parasites has necessitated the development of new drugs that can be used to prevent malaria in those exposed to the parasite.

Tafenoquine is an 8-aminoquinoline with an additional methoxy group at the 2 position, a methyl group at the 4 position, and a 3-trifluoromethylphenoxy substitution at the 5 position of the quinoline ring. It is "an excellent candidate for clinical evaluation as: (1) a causal prophylactic; and/or (2) a blood schizonticidal drug against human malaria parasites, including poly-resistant *P. falciparum* and chloroquine-resistant *P.vivax*", and/or for (3) post-exposure prophylaxis [Peters, 1993]. It is administered as a succinate salt and not as a free base.

The structure of tafenoquine (as the free base) and the related 8-aminoquinoline primaquine are shown:

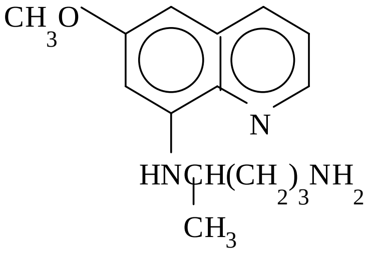

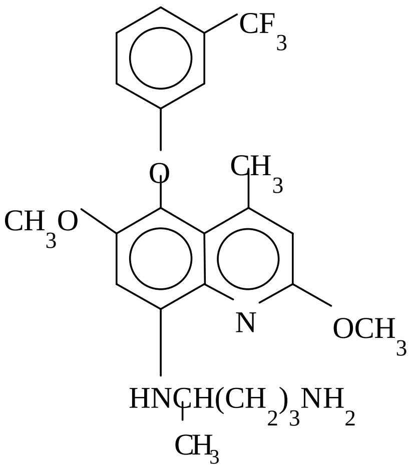

Primaquine **Tafenoquine**

A comprehensive program of clinical pharmacology and phase III studies has been completed - full details are given in the Clinical Investigators Brochure (CIB). Overall the data from these studies indicated that tafenoquine is well-tolerated and effective whether used in chemoprophylaxis of malaria, in eradication following an acute *P.vivax* infection or as a post-exposure prophylactic against *P.vivax* relapse.

In both Phase I and Phase II studies conducted with tafenoquine to date, single and repeat dosing of levels up to 600mg have been generally well tolerated although there is evidence of increased methemoglobin concentration and gastrointestinal disturbances (diarrhea, vomiting and nausea) with increasing dose.

Activity for clearing blood stages of *P. vivax* has not yet been demonstrated for tafenoquine, nor has gametocytocidal activity been demonstrated *in vivo.* However, two studies conducted in Thailand evaluated the safety and efficacy of tafenoquine in the prevention of relapse of *P. vivax* malaria following an acute infection. The first study compared a number of dosing regimens of tafenoquine (300 mg daily for seven days; 500 mg daily for three days followed by a repeat regime one week later; or a single dose of 500 mg), following chloroquine blood schizontocidal treatment, to chloroquine alone. Forty four subjects in total were enrolled in the study and were followed for up to 6 months after treatment to evaluate the rate of *P. vivax* relapse. In subjects who were followed for more than 63 days, the cumulative risk for relapse for the tafenoquine groups was markedly less than placebo.

Treatment Group				
	**Tafenoquine 500mg x1d** **N=7**	**Tafenoquine 500mg x 3d Rpt 1wk later** **N=9**	**Tafenoquine 300mg x 7d** **N=7**	**Chloroquine alone** **N=7**
**Relapses n (Day of FU)**	1 (112)	1 (120)	0	4 (40,43,49,84)
**% protected**	86. %	89%	100%	43%

In the second study further tafenoquine dosing regimens (300mg daily for seven days; 600mg daily for three days, or a single dose of 600 mg), following chloroquine blood schizontocidal treatment, were compared to chloroquine alone or chloroquine followed by a standard eradication course of primaquine.

Treatment Group					
	**Tafenoquine 600mg x1d** **N=15**	**Tafenoquine 600mg x 3d** **N=15**	**Tafenoquine 300mg x 7d** **N=16**	**Chloroquine alone** **N=10**	**Chloroquine followed by Primaquine 15mg x 14d** **N=12**
**Relapses n (Day of FU)**	1[1] (112)	0	0	8 (41,46,48,48, 53,56,63,145)	3 (72,108,143)
**% protected**	93.3%	100%	100%	20%	75%

1. The one relapse in the single dose tafenoquine group may be attributable to vomiting of drug half an hour after dosing – drug levels were not available.

Across the studies, in subjects completing >2 months of follow-up, the incidence of cure (ie lack of relapse) was between 86% and 100% on the tafenoquine regimes, compared to 20-43% receiving chloroquine alone and 75% receiving primaquine. In these studies there was evidence of some gastrointestinal disturbance associated with the administration of tafenoquine.

In 1998/99, 586 soldiers deployed to the Island of Bougainville participated in a randomized open-label, comparator study with the aim of evaluating tafenoquine in the role of a post-exposure prophylactic agent to eradicate and prevent *P. vivax* malaria (Study 252263/049). During deployment in Bougainville, generally for between 2-4 month periods, troops received the standard chemoprophylactic regimens (doxycycline 100 mg daily alone or in combination with chloroquine 300 mg weekly).

For the post-exposure prophylaxis phase, a total of 607 troops received tafenoquine, 400 mg daily (as a single 400 mg dose or a divided dose of 200 mg twice daily) for 3 days, while another 341 soldiers received primaquine 22.5 mg daily for 14 days at the end of the tour; the test drugs were given in various combinations with chloroquine and doxycycline, solely with doxycycline, or alone.

There were 28/607 (4.6%) *P. vivax* eradication failures (i.e. relapses) in the tafenoquine group and 23/341 (6.7%) in the primaquine group, occurring between 4 and 48 weeks after leaving Bougainville.

A further 564 troops were studied, 406 of which received a three day course of 200 mg tafenoquine once a day and 158 of which received a standard primaquine eradication course. There were 20/406 (5%) *P.vivax* eradication failures in the tafenoquine treated group and 7/158 (4%) in the primaquine treatment group during the 12 month follow-up period.

Ophthalmic and Renal Safety

Study SB252263/033 was a randomized, double-blind, comparative study, which evaluated safety, tolerability and effectiveness of tafenoquine and mefloquine for the prophylaxis of malaria in non-immune soldiers deployed in East Timor. The study treatment period started in October 2000 and completed in March 2001. A total of 654 subjects were randomized, 492 tafenoquine subjects and 162 mefloquine subjects.

The prophylaxis regimen in this study consisted of a loading dose during pre-deployment training of 200mg tafenoquine daily for three days or 250mg mefloquine daily for three days. All subjects subsequently received either 200mg tafenoquine or 250mg mefloquine weekly. The chemoprophylactic trial period was six months with a follow-up period of a further six months. Subjects were randomized to treatment in a ratio of 3:1 (tafenoquine : mefloquine).

This study sought to assess potential phospholipidosis effects of tafenoquine, in tissues where these effects could be readily observed, including ophthalmic effects. A sub-group of subjects underwent more detailed evaluation: 77 in the tafenoquine group and 21 in the mefloquine group. No clinically significant effects were seen in lung function, chest x-ray findings or peripheral blood lymphocytes, however in a subgroup of 99 subjects, 69/74 (93%) of tafenoquine subjects, compared to no mefloquine subjects, were noted to have pigmented deposits on the cornea (vortex keratopathy), although no subject suffered impairment or loss of vision. At the 3-month post-treatment follow-up, 61% of these subjects showed complete resolution of these findings, while the remainder had marked reduction of signs of vortex keratopathy. Five-six months after the end of dosing, 90% of subjects had complete resolution and all subjects were shown to have complete resolution approximately 1 year after stopping study drug.

An expert ophthalmology advisory board concluded that the corneal changes were benign, fully reversible and similar to those seen with other drugs, such as chloroquine. They did not consider the changes to be a safety risk that would preclude further development of tafenoquine in man. The time to onset of corneal deposit formation could not be established from Study 033, as assessments were performed at baseline and after 6 months only.

Baseline retinal photography data was needed to determine the relevance of the very minor retinal findings (observed on fundoscopy and fundus fluoroscein angiograms performed in a number of tafenoquine and mefloquine subjects) could not be ascertained. The expert ophthalmology advisory board advised that vision had not been affected in

any of these subjects and that future studies should assess the retina before and after treatment in order to determine whether any retinal drug effect exists

Renal function tests in participants for Study 033 (serum creatinine and BUN) were examined. Both mefloquine and tafenoquine subject's creatinine increased, more so in the tafenoquine group. Tafenoquine was associated with an increase in serum creatinine compared to baseline, in up to 19 % of subjects at any one visit, compared to 10% for mefloquine. At follow-up 6-8% of both groups had creatinine concentrations that were still 25% above baseline. However, whilst trends towards increased creatinine were seen, few subjects had values outside the normal ranges and none were considered clinically significant.

It should be noted that in Study 033 an increase in creatinine concentrations also occurred in mefloquine treated subjects (up to 10% subjects at any time-point) and that there is published evidence of increases in creatinine following chloroquine treatment [Landewe, 1995].

Percent of 033 subjects with creatinine increased from baseline values (+25%)							
**Treatment**	**Days 0-10**	**2-6 weeks**	**7-12 weeks**	**13-21 weeks**	**22-30 weeks**	**Total**	**Follow-up**
**Tafenoquine** 200 mg[1]	5.4 % (26/480)	18.9 % (92/487)	16.3 % (80/490)	8.7 % (42/481)	11.3 % (53/467)	34.8 % (171/491)	6.0 % (28/467)
**Mefloquine** 250 mg[2]	6.5 % (10/155)	9.4 % (15/160)	8.1 % (13/161)	10 % (16/160)	7.1 % (11/156)	21.1 % (34/161)	8.2 % (13/158)

1. TQ 200 mg daily for three days and then tafenoquine 200 mg once a week
2. MQ 250 mg daily for three days and then mefloquine 250 mg once a week

Note: 2 mefloquine and no tafenoquine subjects had creatinine values decreased by 25% from baseline during the study

Subjects with raised creatinine at the end of Study 033 are being evaluated in a follow-up study to provide reassurance that 6 months of dosing with tafenoquine is not associated with long-term chronic renal damage. To date, 131 of the proposed 210 subjects have started the follow up. 100 or 76% of these have completed follow up with no findings. Follow-up is ongoing in 31 subjects. All subjects are expected to have completed follow-up by March, 2003.

This trend of increased creatinine values was also seen in other completed Phase I-III studies.

Reproductive Toxicity

No adverse effects on fertility or embryofetal development (including at maternally toxic doses), or on postnatal survival, were observed in a complete battery of reproductive toxicology studies conducted in male and female rats and female rabbits. Full details of these studies are given in the Clinical Investigators Brochure (CIB).

Please refer to the Clinical Investigator's Brochure for a more complete review of the clinical data on tafenoquine.

1.2. Rationale

To prevent transmission of malaria, a drug, which eradicates both the asexual and sexual stages in the blood, and protects from further infection (prophylaxis) for longer than the 2-3 week incubation time in mosquitoes, would be needed. To prevent transmission of *P. vivax*, a drug which also eradicates the dormant liver stage (hypnozoites) would be needed. No currently available drug acts at all stages of the lifecycle of the plasmodium. Some, such as chloroquine are blood schizontocides and act quickly to kill parasites in the red blood cells. Others, such as primaquine have gametocytocidal activity, killing blood sexual forms thereby preventing transmission to the mosquito. Causal prophylactics, such as atovaquone and primaquine, kill the intrahepatic stages of the developing schizont and prevent the need for prophylaxis after leaving an endemic area. However, only primaquine is able to eradicate the hypnozoites of *P.vivax*.

Tafenoquine has been used very successfully as a prophylactic agent against both *P. vivax* and *P. falciparum* and has proven effective against *P. vivax* malaria hypnozoites (dormant liver stages) in small trials. With its long half-life and efficacy, tafenoquine has great promise as an agent to interrupt the transmission of malaria in large populations with limited repeat dosing.

Although developed primarily as an anti-relapse agent (eradication of the dormant liver stage (hypnozoites) of *P. vivax*), tafenoquine has also been found to possess significant blood schizonticidal activity. Tafenoquine cured established blood-induced infections of the Chesson strain of *vivax* malaria in *Aotus* monkeys. Clearance was obtained at 3 mg/kg and cures were achieved at 9 mg/kg total dose administered in a 3-day oral regimen [Obaldia, 1997]. In rhesus monkeys, a dose of 3.16 mg/kg x 7 days was found to be fully curative against blood stage infections of either *P. cynomolgi* or *P. fragile*. [Puri, 2003].

However, activity for clearing blood stages of *P. falciparum* and *P. vivax* in humans has not yet been demonstrated, nor has gametocytocidal activity been demonstrated *in vivo*. Furthermore, the optimum dose of this therapy for potentially blocking transmission is not yet known. Doses of tafenoquine of 200 mg are typically effective for prophylaxis. However, it is anticipated that higher doses of tafenoquine will be required to clear parasitemia, since other aminoquinolones (chloroquine and mefloquine), also require higher doses for treatment of malaria than are needed for prophylaxis. Based on efficacy, safety, tolerability and pharmacokinetic data gathered during Phase I and II, doses of 600 mg for one day, and 400 mg for three days have been selected as the doses to evaluate tafenoquine as a single agent for the radical cure of *P. vivax*. The 400 mg dose for 3 days was better tolerated than repeat regimens at higher doses (500 mg or 600 mg for 3 days), and has demonstrated efficacy for eradication of *P. vivax* hypnozoites. The 600 mg dose for one day, is the maximum single dose of tafenoquine administered to subjects and has also demonstrated efficacy for eradication of *P. vivax* hypnozoites.

This Phase II study is designed to determine whether a single 600 mg dose or 400mg/day for 3 days of tafenoquine is efficacious, and well tolerated for clearing *P.vivax* malaria infection (blood schizontocidal and gametocytocidal activity) and preventing *P. vivax*

relapse (hypnozoite eradication). It will also further establish the safety and tolerability of these doses of tafenoquine.

As the activity of tafenoquine for clearing blood stages of *P. vivax* has not yet been demonstrated, this study will be conducted in 2 cohorts. Cohort 2 (600mg tafenoquine for 1 day) will not be progressed if insufficient efficacy or safety findings of concern are observed in Cohort 1 (400mg tafenoquine for 3 days).

Enrollment will be suspended in the unlikely event that prespecified criteria for lack of efficacy, renal or ophthalmic findings, or adverse events of severe intensity, including vomiting, diarrhea, or nausea, are met. An Independent Data Monitoring Committee will be used to assist evaluation of relevant data and advise on resuming enrollment of subjects.

2. OBJECTIVE(S)

2.1. Primary

To assess whether a single 600 mg dose or 400mg/day for 3 days of tafenoquine alone can clear/cure *P. vivax* blood stage infections.

2.2. Secondary

To assess whether a single 600 mg dose or 400mg/day for 3 days of tafenoquine alone can prevent *P. vivax* relapse for 2, 3 and 4 months.

Determination of Parasite, Gametocyte and Fever Clearance Time (PCT, GCT, and FCT).

To establish the safety and tolerability of these doses of tafenoquine.

To characterize the population pharmacokinetics of tafenoquine.

3. ENDPOINT(S)

3.1. Primary

The primary efficacy endpoint will be:

- Day 28 cure rate. A subject will be considered a success (cure) if they have an Adequate Clinical Response (ACR).

  <u>Adequate Clinical Response</u>

  - -The subject does not develop any of the conditions of ETF or LTF.
  - -Parasitological clearance has been confirmed throughout the follow-up period (Day 28).

- A subject will be considered a failure if they meet the definition of Early Treatment Failure (ETF) or Late Treatment Failure (LTF) as defined below.

  Early Treatment Failure

  - Parasitemia on Day 7

  Late Treatment Failure

  - Parasitemia recurring after Day 7 up to and including Day 28 (same species as on Day 0).

Subjects who are treatment failures will be withdrawn from the study and given appropriate anti-malarial treatment according to [redacted] guidelines. (All randomized subjects who received at least one dose of study medication (i.e. tafenoquine or chloroquine/primaquine) will be included in the Safety and ITT analysis populations.)

3.2. Secondary

The secondary endpoints will be:

Efficacy and Safety:

- **Proportion of subjects without relapse of *P. vivax* at 2, 3 and 4 months.**
- **Determination of Parasite, Gametocyte, and Fever Clearance Time (PCT, GCT, and FCT).**
- **Safety: To evaluate the safety and tolerability of the tafenoquine dosing regimens.**

Pharmacology:

- **Population pharmacokinetic parameters (CL/F, V/F, ka) will be estimated for tafenoquine.**

4. STUDY DESIGN

4.1. General Study Design

This will be a randomized, active-control, double-blind, double-dummy study conducted in 2 sequential cohorts in a total of 140 subjects enrolled in order to obtain 120 evaluable (60/cohort) male and female subjects age 20-60 years with confirmed *P. vivax* malaria.

The study will be conducted at the [redacted] located in [redacted] Thailand (hereafter referred to as the [redacted] This institution enjoys a worldwide reputation for research on the treatment of malaria and malaria-related complications. The Hospital has a staff of approximately 50 physicians, several hundred nurses, and 15-20 laboratory technicians.

In addition to a large outpatient clinic, [redacted] has 250 beds, a 6 bed ICU capable of supporting hemodialysis (for malaria-related renal complications), mechanical ventilation (for the respiratory support of cerebral malaria), and laboratory facilities capable of supporting all aspects of malaria diagnostics and treatment, from expert microscopy to blood banking facilities capable of supporting exchange transfusions for severe malaria.

Study volunteers will be recruited by a study investigator or nurse, from persons presenting either at the [redacted] outpatient clinic or admitted to the inpatient ward following transfer from the [redacted] clinic, or clinics from [redacted] or [redacted] provinces, for evaluation of fever. Subjects deemed eligible for the study will be consented and enrolled if eligible and willing to participate. Subjects found not eligible or unwilling to participate in the study will be treated as clinically warranted. Subjects originally transported from the [redacted] clinic, or clinics from [redacted] or [redacted] provinces, who are found not eligible or unwilling to participate in the study, will receive free treatment as clinically warranted and provided free transportation back to their respective rural malaria clinics when clinically warranted. Subjects enrolled into the study will be hospitalized at the [redacted] for the first 29 days of the study and will be asked to remain in a malaria non-endemic area until 90 days after the start of the study for follow up with scheduled assessments at Days 60 and 90. While hospitalized subjects enrolled in this study will have the same [redacted] visitation policy as are those subjects who are not research participants.

Subjects will be contacted at Day 120 for a follow-up blood smear. Subjects will be given tafenoquine alone or the standard regimen of chloroquine followed by primaquine (2:1 ratio, 40:20 subjects in each cohort). Subjects will provide written informed consent and will undergo screening and baseline procedures before the start of the study. Subjects in Cohort 1 will be randomized to receive either tafenoquine (400mg base) and chloroquine placebo for 3 days, followed by primaquine placebo for 14 days, or chloroquine (1000 mg chloroquine phosphate) and tafenoquine placebo for 2 days, followed by chloroquine (500 mg chloroquine phosphate) and tafenoquine placebo for 1 day, followed by primaquine, 15 mg/day for 14 days.

The efficacy (Day 28 cure rate) of the dosing regimen in Cohort 1 will be compared to prespecified criteria (as detailed in Section 11.3) once all subjects have completed the 90 day assessment. These efficacy results and the safety profile will be examined before enrolling Cohort 2. Subjects in Cohort 2 will be randomized to receive either tafenoquine (600 mg base) and chloroquine placebo for 1 day, followed by chloroquine placebo for 2 days, followed by primaquine placebo for 14 days, or chloroquine (1000 mg chloroquine phosphate) and tafenoquine placebo for 1 day, followed by chloroquine (1000 mg chloroquine phosphate) for 1 day, followed by chloroquine (500 mg chloroquine phosphate) for 1 day, followed by primaquine, 15 mg/day for 14 days.

Subjects will remain in the study for 121 days from start of treatment to end of routine follow-up. Further follow-up will occur for any renal or ophthalmic findings at the discretion of the investigator.

4.2. Risks and Benefits to Research Subjects

4.2.1. Risks to Research Subjects

The subject may experience a brief moment of physical discomfort during the finger-prick procedure and the venipuncture and there is a possibility of bruising and/or infection at the site of the finger-prick or venipuncture. A maximum of 160 ml of blood will be taken from each subject during the study.

Subjects may experience side effects from the drugs. Side affects associated with chloroquine may include nausea, vomiting, blurred vision and headache. More frequent side effects associated with primaquine include nausea, vomiting and abdominal pain. The primary clinical events associated with tafenoquine include gastrointestinal disturbances (vomiting, nausea, diarrhea and abdominal pain) and headaches.

Subjects may experience slight stinging in the eyes after dilation of the pupils with tropicamide 1.0% and phenylephrine 2.5% for the corneal and fundus examinations.

There is increased risk of intravascular hemolysis associated with these drugs in subjects who are G6PD deficient. All subjects considered for this study will be screened for G6PD deficiency. Although it is not anticipated that any subject will be mistakenly enrolled in this study, if this should occur in exceptional circumstances, hemolysis would occur within the initial days following the first dose of trial medication, so the subjects will already be in a hospital setting, therefore prompt medical attention will be readily available.

In a sub-group of study SB252263/033, tafenoquine was shown to cause pigmented deposits on the cornea (vortex keratopathy), after 6 months of weekly dosing in adults. The time to onset of corneal deposit formation could not be established, as assessments were performed at baseline and after 6 months only. These deposits did not result in impairment or loss of vision and were shown to have complete resolution approximately 1 year after stopping study drug. It is not anticipated that these deposits will occur following 3 days of dosing with tafenoquine, however, this will be monitored through the eye assessments at baseline, Day 28 and Day 90.

In this same study some minor findings were seen at the end of the study in the retina. No subject had problems with vision and no major retinal changes were seen during follow-up. The relevance of these minor retinal findings could not be ascertained as baseline retinal photography data had not been obtained, however, this will be monitored through the eye assessments at baseline, Day 28 and Day 90.

In earlier studies, trends towards increased creatinine were seen in subjects receiving tafenoquine. Few subjects had values outside the normal ranges and none were considered clinically significant. These data were reviewed by a panel of clinical nephrologists which concluded that the existing data cannot exclude the occurrence of renal damage, although this is considered unlikely. Kidney function will be monitored through clinical chemistry assessments at baseline and at Days 3, 7, 14, 21, 28 and 90, and through urinalysis at baseline and Days 7 and 28.

All subjects will be closely monitored (daily for 29 days and monthly for a further 3 months) and quickly treated should any signs or symptoms of malaria be detected.

Should a subject be injured as a direct result of participating in this study, medical care will be provided, at no cost, for that injury. Injured subjects will also receive a travel and a per diem allowance to cover the cost of food and traveling to medical appointments required to treat a study related injury. Subjects will also be compensated for lost income, 170 Baht/day, resulting from treatment for a study related injury.

4.2.2. Benefits to Research Subjects

As subjects will be hospitalized during the anti-malarial compound administration periods, all treatment doses of chloroquine, tafenoquine, and primaquine will be administered under supervision by medical/nursing staff trained in drug administration, and any change in the course of their infection or any adverse experiences will be recognized and treated more rapidly than would normally occur in the their rural home setting.

Subjects will be immediately treated for any reappearance of parasitemia that occurs during the 3 month follow-up period. Subjects who fail initial therapy, based on parasitological parameters, will be treated with an alternative regimen that is known to be effective. In addition, the subjects will be examined and treated for other concurrent illnesses.

Subjects will also receive medical attention and appropriate standard medical care or referral should they become ill during the study. This will be done at no cost to the subject.

5. STUDY POPULATION

Malaria used to be endemic throughout Thailand. Overall, still 300,000 cases of malaria occur yearly, approximately 50% of which are *Plasmodium vivax*. Due to deforestation and other land use activities and a model public health control program, malaria is now limited to the mountainous forested and forest fringe regions along the border. The central plains of Thailand including [redacted] are largely malaria free, except for sporadic cases in travelers. Historically, approximately 2/3 of volunteer subjects enrolled in research trials at the [redacted] are from residents of this malaria-free zone who acquired the infection while traveling to an endemic region, and who present to this hospital for evaluation and treatment of their febrile illness. These persons come from all walks of life, whose business or pleasure takes them in to malaria-endemic areas of the country.

A lesser number of volunteers may be recruited from a population of those who come to [redacted] for free clinical services from a [redacted] outpatient facility in [redacted] district, [redacted] Province, located about 2 hours from [redacted] The Tropical Medicine Trust Fund, under royal patronage by the king's sister, Her Royal Highness, Princess [redacted] established the satellite clinic of the [redacted] in [redacted] in 1995 with a mandate to provide free treatment and prevention

programs for tropical diseases both on-site, as well as free transportation and treatment as needed at the hospital in [redacted] The local population of [redacted] consists largely of Thai and Karen involved in farming, forestry or factory work with annual wages of 28000-36000 THB ($680-850). The mountainous areas of [redacted] are part of the traditional homeland of the Karen peoples, so here they will have Thai residency cards and speak the Thai language. Education to the eighth grade level is typical. These persons routinely use this [redacted] outpatient facility in [redacted] for medical care of a variety of tropical diseases, including malaria, leptospirosis and helminthiasis. A daily shuttle and regular ambulance service will bring anyone needing or desiring medical services from the [redacted] outpatient facility to the [redacted] for free diagnosis and treatment. Some are referred (e.g. severe malaria) or request to go to [redacted] for further evaluation and treatment. For example, in 2001, the [redacted] clinic treated 719 falciparum malaria patients; 229 of whom were transferred to [redacted] No recruitment for research trials occurs onsite in [redacted] but those who come to [redacted] for clinical care may be assessed for inclusion in any ongoing research trials at the [redacted] during their medical evaluation at the hospital.

As a recognized facility specializing in the care of patients suffering from tropical infectious diseases, the [redacted] also serves as a referral tertiary care facility providing care to patients presenting to government operated rural malaria clinics located along the Thai-Myanmar border. Typically, such clinics are located in small villages in [redacted] and [redacted] provinces, approximately 4-7 hours from [redacted] Although malaria clinics are adequate for the routine diagnosis and treatment of malaria, these facilities operate as outpatient facilities only. As is the case with patients originating from the [redacted] facility, some patients presenting to these government malaria clinics may request further treatment and evaluation at the [redacted] In this event, the same [redacted] operated ambulance service will be employed to transport the patient from the malaria clinic to [redacted] Although no recruitment for research trials occurs in the malaria clinics, such patients may elect to participate in research trials while undergoing evaluation and treatment at [redacted]

5.1. Number of Subjects

A total of approximately 140 male and female subjects between the ages of 20 and 60 years inclusive will be recruited for this study in order to obtain 120 evaluable subjects (40 evaluable subjects in each of the 2 cohorts for the tafenoquine arm and 20 evaluable subjects in each of the 2 cohorts for the comparator arm).

5.2. Eligibility Criteria

5.2.1. Inclusion Criteria

A subject will be eligible for inclusion in this study only if all of the following criteria apply:

1. Positive smear for *P. vivax*.

2. Parasite density > 500 and < 200,000/µl
3. Age: 20-60 years old
4. Willing to sign consent form
5. Willing to be hospitalized for 29 days and remain in a malaria free region for 60 days thereafter for follow-up.
6. A female is eligible to enter and participate in this study if she is of:
    a non-childbearing potential (i.e., physiologically incapable of becoming pregnant), including any female who is post-menopausal or,
    b child-bearing potential, has a negative pregnancy (urine or serum) test at screen, and agrees to comply with recognized contraceptive methods during the treatment stage of the study and for a period of 12 weeks after stopping study drug. Recognized contraceptive methods include, abstinence, implants of levonorgestrel, injectable progestogen, or appropriate double barrier methods using licensed contraceptives such as diaphragm and condom (by the partner) or intrauterine device and condom. The use of oral/patch contraceptives during the study is not considered sufficient contraceptive protection.

5.2.2. Exclusion Criteria

A subject will not be eligible for inclusion in this study if any of the following criteria apply:

1. Mixed malaria infections by Field's stain.
2. Female subjects who are pregnant, lactating or unwilling/unable to comply with recognized contraceptive methods during the treatment stage of the study and for a period of 12 weeks after stopping study drug.
3. Symptoms of severe vomiting (no food or inability to take food during the previous 8 hours).
4. Demonstrated glucose-6-phosphate dehydrogenase deficiency.
5. Subject has taken other anti-malarials (mefloquine, primaquine, chloroquine) within the past 30 days by history
6. Clinically significant illness (intercurrent illness e.g. pneumonia, pre-existing condition e.g. renal disease, malignancy or conditions that may affect absorption of study medication e.g. severe diarrhea or any signs of malnutrition as defined clinically).
7. Clinically significant abnormal laboratory values as determined by history, physical examination or routine blood chemistries and hematology values (laboratory guideline values for exclusion are hemoglobin <7 gm/dL, platelets < 50,000/µl, White Blood Cell count (WBC) < 2000/µl, serum creatinine >2.0mg/dL, or ALT or AST more than 3 times the upper limit of normal for age.
8. History of allergy to chloroquine, mefloquine, tafenoquine, primaquine or any other 8-aminoquinolines.

9. Subject has taken another investigational drug within 30 days or 5 half lives (whichever is longer), of study start.
10. History of previous eye surgery or have evidence of corneal or retinal abnormalities identified in baseline ophthalmological examination.
11. Subjects taking concomitant medications likely to affect renal or ophthalmic function or that are known to be metabolized primarily by the cytochrome P450 isoforms 3A4/5 and 2C9 and whose therapeutic effect occurs within a narrow plasma concentration range (e.g. warfarin, ketoconazole).
12. Subjects whom, after examination by the study ophthalmologist, are judged to be at risk for acute angle closure glaucoma.
13. Females who are pre-menarchal.

5.2.3. Other Eligibility Criteria Considerations

To assess any potential impact on subject eligibility with regard to safety, the investigator must refer to the following document(s) for detailed information regarding warnings, precautions, contraindications, adverse events, and other significant data pertaining to the investigational product(s) being used in this study: Clinical Investigator's Brochure.

6. STUDY ASSESSMENTS AND PROCEDURES

A time and events schedule can be found in Appendix 1

6.1. Demographic and Baseline Assessments

At the screening/baseline visit the study will be fully explained to the subjects and written informed consent obtained. Subjects will be assessed as to whether they meet the inclusion exclusion criteria. Demographic details (age, sex, weight, height) and medical history and concomitant medication details will be recorded. Vital signs (pulse, blood pressure, respiratory rate, and temperature) will be recorded and a physical exam will be performed. A blood smear to confirm *Plasmodium vivax* malaria will be performed. A blood sample will be taken for biochemistry and hematology analysis and a urine sample provided for urinalysis. All subjects will have their G6PD status tested at screening. All female subjects will undergo a pregnancy test (urine or serum); pregnant subjects may not enter the study.

Baseline eye examinations (visual acuity, amsler grid, color vision, corneal and retinal examination/photography) will be performed prior to first dose of tafenoquine or comparator medication if possible. If the scheduling of the full eye exam will significantly delay the start of treatment (> 10 hours) or if the subject is deemed by the investigator to be too ill to undergo the complete eye examination, a basic eye exam to assess vision problems will be performed prior to dosing. The full eye exam will then be conducted as soon as possible but no later than within 36 hours of receiving the first dose of study drug.

At the baseline examination the study ophthalmologists will measure a subject's intraocular pressure and anterior chamber depth during the standard eye examination. If the anterior chamber is shallow, gonioscopic examination of the chamber angle will be performed to exclude an occludable angle prior to dilating the pupil with mydriatics. Subjects at risk for acute angle closure glaucoma will be excluded from the study.

The following ophthalmic tests will be performed:

- Macular Function Tests:

  Amsler Grid, Humphrey 10-2 Visual Field, Macular stress test, High contrast visual acuity (HCVA) – ETDRS chart, Color vision – PIP plates & Lanthony 40 hue

- Digital Photography:

  Corneal and retinal digital photographs

6.2. Safety

6.2.1. Vital Signs

Vital signs (pulse rate, blood pressure, respiration rate) will be measured and the results recorded in the eCRF at baseline and on Days 1-7, 14, 28, 60, and 90. Body temperature will be measured every 12 hours (± 2 hours) after the baseline measurement through Day 7, and then daily on Days 14, 28, 60, and 90.

6.2.2. Clinical Chemistry, Methemoglobin and Hematology

A blood sample will be taken for biochemistry analysis at baseline and on Days 3, 7, 14, 21, 28 and 90. For biochemistry the following parameters will be assessed: blood urea nitrogen (BUN), creatinine, glucose, sodium, potassium, chloride, total carbon dioxide, calcium, aspartate aminotransferase (AST), alanine aminotransferase (ALT), gamma-glutamyl transferase (GGT), alkaline phosphatase, total bilirubin, albumin, and total protein. A blood sample will be taken for hematology analysis at baseline, at Days 3, 7 and 14. For hematology the following parameters will be assessed: complete blood count, including red cell indices and white cell differential, and platelet count. A blood sample will be taken to determine methemoglobin concentration at baseline and Days 3, 7, and 21.

6.2.3. Renal Assessments

Serum creatinine will be measured as part of the biochemistry analysis at baseline and on Days 3, 7, 14, 21, 28, and 90 (see Section 6.2.2). If serum creatinine is increased ≥26.6 μmol/L (0.3 mg/dL) compared to baseline, it will be repeated within 24 hours. Subjects with confirmed serum creatinine increases of ≥26.6 μmol/L (0.3 mg/dL) will be reassessed 7 and 14 days after the initial increase of ≥26.6 μmol/L (0.3 mg/dL), or more frequently at the discretion of the investigator, to determine if the serum creatinine level is returning to baseline. If a sustained (≥ 14 days) serum creatinine increase of ≥26.6

μmol/L (0.3 mg/dL) is confirmed it will be upgraded to GSK medically defined serious for fast-tracking on to the safety database and for rapid follow-up. A morning "clean-catch" (midstream) urine sample will be collected on the following day for urinalysis and will be stored at ≤ -20°C for possible measurement of urine protein:creatinine ratio. If a sustained (≥ 14 days) serum creatinine increase of ≥26.6 μmol/L (0.3 mg/dL) is confirmed in ≥8 subjects within a cohort, enrollment of further subjects will be put on hold until the data can be reviewed by an IDMC for recommendation on continued enrollment of subjects. Follow up of subjects will continue to assess the reversibility of any findings.

6.2.4. Urinalysis

A "clean-catch" (midstream) urine sample will be collected at baseline and a morning urine sample will be collected at the Day 7 and Day 28 visits, and the morning after a sustained (≥ 14 days) serum creatinine increase of ≥26.6 μmol/L (0.3 mg/dL) from baseline is confirmed. The time of urine sample collection will be recorded and the following parameters will be assessed: specific gravity and/or urine osmolality, pH, glucose, protein, blood and ketones by dipstick. If urine dipstick analyses are abnormal, a urine microscopic examination will be performed. Abnormal urine dipsticks will be repeated every week until normalized or hospital discharge. All urine samples will be stored at -20°C or colder for possible measurement of urine protein:creatinine ratio.

6.2.5. Ophthalmic Assessments

Ophthalmic safety will be assessed in all subjects using the following tests at baseline and at the Day 28 and Day 90 visits.

- Macular Function Tests:

  Amsler Grid, Humphrey 10-2 Visual Field, Macular stress test, High contrast visual acuity (HCVA) – ETDRS chart, Color vision – PIP plates & Lanthony 40 hue

- Digital Photography:

  Corneal and retinal digital photographs

If at any timepoint, subjects develop retinal changes from baseline that cause clinical concern (as defined below), it will be upgraded to GSK medically defined serious for fast-tracking on to the safety database and for rapid follow-up. If retinal changes from baseline that cause clinical concern are seen in ≥2 subjects within a cohort enrollment of further subjects will be put on hold until the data can be reviewed by an IDMC for recommendation on continued enrollment of subjects. Subject will be followed up to assess the reversibility of any findings.

- Decreased vision (less than 20/20)
  - Loss of greater than 3 lines of best-corrected visual acuity in either eye. A significant change is 0.08 logMAR (4 letters on the high contrast chart; one line), the indication for treatment withdrawal is 0.3 log MAR (15 letters decrease on the high contrast chart, 3 lines).

- Bull's eye retinopathy
  - Presence confirmed by clinical assessment and digital photographs
- Distortions observed on the Amsler Grid test
  - Amsler should be done twice separated by at least 10 minutes to confirm presence of distortion. The indication for a treatment withdrawal is the presence of a repeatable area of distortion (metamorphopsia) or scotoma covering more than one block of the Amsler grid (greater than 1 degree of visual angle).
- Abnormal color vision
  - Development of a repeatable color vision defect diagnostic of color vision abnormality on the L'Anthony 40 Hue test. An increase of 2 in the number of pages missed on the PIP warrants further investigation. The L'Anthony 40 Hue test will be used to quantify the defect. Greater than 2 reversals is clinically significant, the indication for treatment withdrawal is 4 or more reversals.
- Development of a scotoma on visual field testing
  - Development of a repeatable scotoma evident on 2 visual field tests separated by at least 30 minutes, not present on baseline examination. A 5 decibel decrease in sensitivity (either local or overall visual field) is considered clinically significant, the indication for treatment withdrawal is 10 decibels decrease in sensitivity (scotoma or overall field)

6.2.6. Pregnancy

6.2.6.1. Pregnancy testing

All female subjects will undergo a pregnancy test (urine or serum) at baseline. Pregnant women will not be eligible for entry into the study. A pregnancy test (urine or serum) will also be performed on female subjects at the Day 7, Day 21, and Day 90 visit.

6.2.6.2. Time period for collecting pregnancy information

The time period for collection of information on the occurrence of pregnancy will be from the baseline visit to the Day 120 follow-up visit.

6.2.6.3. Action to be taken if pregnancy occurs

The investigator, or his/her designee, will collect pregnancy information on any female subject who becomes pregnant while participating in this study. The investigator, or his/her designee, will record pregnancy information on the appropriate form and submit it to USAMRMC and GSK within 2 weeks of learning of a subject's pregnancy. Any subject who becomes pregnant during the treatment phase of the study will have study medication withdrawn, and be withdrawn from the study. Any subject who becomes pregnant after the treatment phase of the study, may continue in the study at the discretion of the investigator. Any subject who received at least one dose of study medication (i.e. tafenoquine or chloroquine/primaquine) and is withdrawn from the study due to pregnancy will be included in the Safety and ITT analysis populations. Any subject who becomes pregnant during the study will also be followed to determine the

outcome of the pregnancy. Information on the status of the mother and child will be forwarded to USAMRMC and GSK. Generally, follow-up will be no longer than 6 to 8 weeks following the estimated delivery date. Any premature termination of the pregnancy will be reported.

While pregnancy itself is not considered to be an AE or SAE, any pregnancy complication or elective termination of a pregnancy for medical reasons will be recorded as an AE or a SAE, as described in Section 10.6., "Recording of AEs and SAEs" and will be followed as described in Section 10.8., "Follow-up of AEs and SAEs."

A spontaneous abortion is always considered to be a SAE and will be reported as described in Section 10, “Adverse Events (AE) and Serious Adverse Events (SAE).” Furthermore, any SAE occurring as a result of a post-study pregnancy **and** is considered reasonably related to the investigational product by the investigator, will be reported to USAMRMC and GSK as described in Section 10.11., "Post-study AEs and SAEs." While the investigator is not obligated to actively seek this information in former study participants, he/she may learn of an SAE through spontaneous reporting.

6.3. Efficacy

6.3.1. Primary Efficacy

Thick and thin blood smears for malaria will be obtained by finger prick at baseline (Day 0) and then every 12 hours (± 2 hours) up to and including Day 7, until the blood smear becomes negative. Parasites and gametocytes will be considered cleared if 2 consecutive blood smears are negative. Once confirmed to be negative, blood smears will be obtained once a day up to and including Day 7. Subjects whose smears first become negative with the initial smear on Day 7, must be negative on the repeat smear to be considered negative. Subjects positive for parasitemia on any smear on Day 7 will be considered early treatment failures and withdrawn from the study and will be included in the Safety and ITT analysis populations. After Day 7, thick and thin blood smears will be obtained once a day by finger prick on Day 14, Day 28, Day 60, Day 90, and Day 120 (see Study Schedule, Appendix 1). The subject will be asked to return to the clinic if they develop signs or symptoms of malaria. Additional smears will be done every day that a subject has signs or symptoms that could be malaria (unscheduled slides).

All blood smears will be stained with Field’s stain reagents and examined by two microscopists who are blinded to each other’s results. Parasite densities will be calculated based on a count of parasites per 200 WBC on a thick film or parasites per 1000 red blood cells (RBC) on a thin film. A total of 200 high power (1000 X) oil immersion fields will be examined before a blood smear is considered negative. In case of a difference in results (positive/negative; species diagnosis; or > 2-fold difference in parasite density) between the two microscopists, the blood smear will be re-examined by a third microscopist (a senior microscopist acting as referee) and the third reading will be accepted as the final result. Smears should be repeated after 12-24 hours to confirm parasitemia and species.

Following initial clearance of parasitemia, blood smears identified as positive for *P. vivax* through this procedure will result in the subject being considered as having had a relapse infection (i.e. have met the study endpoint). If a subject is considered as having a relapse, it should be recorded whether the subject was asymptomatic or symptomatic. Subjects who develop *P. falciparum* malaria prior to completion of the treatment phase will be withdrawn from the study and will be given appropriate treatment. Subjects who develop *P. falciparum* malaria prior to the Day 28 assessment will be given appropriate treatment and will not be evaluable for the primary endpoint (Day 28 cure rate for *P. vivax*). Subjects who present with *P. falciparum* malaria at the Day 28 assessment will be given appropriate treatment and will be evaluable for the primary endpoint (Day 28 cure rate for *P. vivax*). Any subject who develops *P. falciparum* malaria after completion of the treatment phase will continue to be monitored for *P. vivax* relapse infection following appropriate treatment. All randomized subjects who received at least one dose of study medication (i.e. tafenoquine or chloroquine/primaquine) will be included in the Safety and ITT analysis populations.

Molecular diagnosis with polymerase chain reaction (PCR) or antibody based methods may be performed. Microscopic species identification and determination of parasite antigen and deoxy ribonucleic acid (DNA) levels may be attempted in order to distinguish re-infection from recrudescence. These analyses will be performed on aliquots of blood samples collected for clinical biochemistry analysis and stored frozen at approximately -70°C or colder, from baseline, Day 7, Day 14, Day 28, and Day 90, or at the time of withdrawal due to treatment failure.

All blood smears must be retained at the study site.

6.3.2. Secondary Efficacy Measures

Prevention *P. vivax* relapse for 2, 3 and 4 months: blood smears will be obtained at Day 60, Day 90 and Day 120 to confirm the continued absence of *P.vivax* parasitemia.

Parasite and gametocyte clearance time (PCT and GCT): Serial blood smears to detect the presence of *P. vivax* parasites and gametocytes, conducted , every 12 hours (± 2 hours) after the baseline smear up to an including Day 7, until the blood smear becomes negative will be utilized to determine the time (in half-days) to clearance from the time of initial study drug administration. Parasites and gametocytes will be considered cleared if 2 consecutive blood smears are negative.

Fever Clearance Time (FCT): Body temperature, measured every 12 hours (± 2 hours) after the baseline measurement through Day 7 will be used to determine the time (in half-days) from initiation of treatment until a subject's temperature decreases to 37.2 °C and remains at or below that level for a minimum of 24 hours.

6.3.3. Efficacy Monitoring During Enrollment

For both Cohort 1 and Cohort 2, if ≥4 Early Treatment Failures (ETFs) occur among the first 21 subjects enrolled into the Cohort, further enrollment of subjects will be

suspended, and the data will be sent to an independent Statistical Data Analysis Center (SDAC) for treatment unblinding and analysis. If based on these data, the SDAC confirms that the actual response rate amongst evaluable tafenoquine subjects is ≥70%, enrollment will be re-initiated. However if the SDAC confirms that the actual response rate amongst evaluable tafenoquine subjects is below 70%, enrollment will remain suspended and the SDAC will be required to call an ad-hoc IDMC meeting. The IDMC will be requested to review all relevant safety and efficacy data and to provide guidance as to how to proceed with enrollment. This may include recommending continuation, scheduling a second interim look, a change to the protocol, or stopping the study.

As described above, a single look (rather than the continued monitoring of the number of ETFs) has been chosen in order to monitor efficacy during enrollment to each Cohort. This has been chosen as, although an early review entails using a limited quantity of data with high associated variability, 21 subjects is believed adequate in order to provide a strong indication if a lack of efficacy truly does exist. Using ETFs as the criteria for suspending enrollment and an IDMC review will allow determination of a true lack of efficacy at the earliest possible timepoint and thereby keep the number of subjects exposed to an ineffectual treatment regimen, to a minimum. Additionally subjects will be hospitalized and closely monitored during this time period and would receive rapid treatment upon relapse.

6.4. Pharmacokinetics

6.4.1. Sample Collection:

Blood samples will be collected from all subjects for population pharmacokinetic analysis at the following designated times:

i. One sample between 1 and 10 hours post-first dose
ii. One sample between 36 and 42 h post first dose (12-18 h post second dose)
iii. One sample 48 h post first-dose (pre-third dose for 400 mg x 3 d)
iv. One sample between 72 and 168 h post first dose (Day 3- Day 7)
v. Day 12-20
vi. Day 28-30
vii. At recurrence of *P. vivax* after Day 7 up to and including Day 28 if a PK sample has not been drawn within the previous 24 hours

Sampling times within a particular window should be spread across that window among subjects rather than being grouped at extreme ends of the window (eg. 1-10 h window – not all samples taken at 1 h or 10 h). Working instructions will be developed and followed to insure that sampling times will be adequately bracketed within the sampling window. The **date** and exact **time** of each sample will be recorded on the sample tube, the

case report form (CRF) and Quest requisition form. The **date** and exact **time** of administration of all doses must also be recorded in the CRF.

If *P. vivax* recurs after Day 7 up to and including Day 28, a PK sample will be taken to assess tafenoquine levels at the time of recurrence. This sample will be taken only if a PK sample has not been taken within the previous 24 hours. The **date** and exact **time** of the sample will be recorded on the sample tube, the case report form (CRF) and Quest requisition form.

6.4.2. Processing of Samples

Pharmacokinetic blood samples (2.5 mL) will be drawn into Ethylenediaminetetraacetic Acid (EDTA) tubes. The whole blood sample will be stored at approximately 4°C or on water ice until centrifuged, and must be centrifuged, separated and decanted within 2 hours of the sample collection. Plasma will be transferred to appropriately labeled polypropylene tubes. Plasma samples will be stored frozen at approximately -70°C or colder until shipped. GlaxoSmithKline or its designee will store the plasma samples at approximately -70°C or colder until analyzed. Shipping of samples will be co-ordinated by Quest Diagnostics and should be shipped intermittently during the study period.

Plasma concentrations of tafenoquine will be determined by Quintiles Limited, Edinburgh, Scotland using an approved assay. The drug analysis aspects of this study will be done under the direction of Worldwide Bioanalysis Dept., DMPK, GlaxoSmithKline Pharmaceuticals, UK. The randomization treatment code will be provided to the Worldwide Bioanalysis Dept. in order to avoid analyzing samples from subjects receiving the active comparator. All drug analysis data will be stored in the Archives, GlaxoSmithKline, Research and Development or its designee.

6.5. Other Studies

Pretreatment (and recurrence) aliquots of each subject's *P. vivax* infected blood will be cryopreserved in Dimethyl Sulfoxide (DMSO) or glycerol. Archiving viable parasites in this manner will potentially allow for future *in vitro* drug resistance testing. Samples may also be used to study the effect of the study drug(s) on parasite killing or inhibition. Archiving samples in this manner will also allow for future PCR or parasite genotyping studies, which may aid in determining the true efficacy of the study drug in the event of late treatment failure. Sample processing procedures will be provided in a separate document.

7. INVESTIGATIONAL PRODUCT(S)

7.1. Description of Investigational Product

Tafenoquine will be supplied as a size 1 hard gelatin capsule comprised of an opaque standard grey cap and medium orange body, containing tafenoquine 200mg (pure free base). A matched placebo will be identical in external appearance to the active capsules.

Chloroquine will be supplied as an opaque pink size DB-AA hard gelatin capsule, containing an overencapsulated 250mg chloroquine phosphate tablet. A matched placebo will be identical in external appearance to the active capsules.

Primaquine will be supplied as a size 1 hard gelatin capsule comprised of an opaque standard grey cap and medium orange body, containing two overencapsulated 7.5mg primaquine phosphate tablets (15mg free base in total). A matched placebo will be identical in external appearance to the active capsules.

The recommended storage condition for all medication is controlled room temperature, 15-30°C.

The investigational drug product (tafenoquine) was manufactured by:
GlaxoSmithKline
Magpie Wood
Manor Royal
Crawley
Sussex
RH10 2QJ
UK

The commercial supplies of chloroquine and primaquine used for overencapsulation were sourced from:
1. Choroquine (as Avloclor[†] tablets containing 250 mg chloroquine phosphate)
AstraZeneca UK Ltd.
600 Capability Green
Luton
LU1 3LU
UK

2. Primaquine (as Primacin[*] tablets containing primaquine phosphate equivalent to 7.5 mg primaquine)
Boucher and Muir Pty Ltd
Willoughby Road
Crows Nest
New South Wales
NSW 2065
Australia

Packaging of the investigational drug product, as well as manufacture and packaging of the matching placebos and overencapsulated comparator products (chloroquine and primaquine) was performed by:
GlaxoSmithKline

[†] Avloclor is a Trade Mark of AstraZeneca UK Limited. Registered in US Patent and Trademark Office.

[*] Primacin is a Trade Mark of Boucher & Muir Pty Limited.

Third Avenue
Harlow
Essex
CM19 5AW
UK

7.2. Dosage and Administration

Subjects within Cohort 1 will be randomized to one of the two treatment groups:

1. Tafenoquine: 2 capsules (200mg base/capsule for a total of 400mg base) and 4 chloroquine placebo capsules for 2 days, followed by 2 tafenoquine capsules and 2 chloroquine placebo capsules for 1 day, followed by 1 primaquine placebo capsule/day for 14 days.

2. Chloroquine: 4 capsules (250mg chloroquine phosphate/capsule for a total of 1000 mg chloroquine phosphate) and 2 tafenoquine placebo capsules for 2 days, followed by 2 chloroquine capsules (500mg chloroquine phosphate) and 2 tafenoquine placebo capsules for 1 day, followed by 1 primaquine capsule (15 mg base/capsule) per day for 14 days.

Subjects within Cohort 2 will be randomized to one of the two treatment groups:

1. Tafenoquine: 3 capsules (600 mg base total) and 4 chloroquine placebo capsules for 1 day, followed by 4 chloroquine placebo capsules for 1 day, followed by 2 chloroquine placebo capsules for 1 day, followed by 1 primaquine placebo capsule/day for 14 days.

2. Chloroquine: 4 capsules (250mg chloroquine phosphate/capsule for a total of 1000 mg chloroquine phosphate) and 3 tafenoquine placebo capsules for 1 day, followed by 4 chloroquine capsules (1000 mg chloroquine phosphate) for one day, followed by 2 chloroquine capsules (500mg chloroquine phosphate) for one day, followed by 1 primaquine capsule (15 mg base/capsule) per day for 14 days.

Study medication will be given with food. If a subject vomits within 1 hour following dosing, a repeat dose should be taken. If a subject sequentially vomits two doses of study medication he/she will be considered intolerant to study medication and be withdrawn from the study. All randomized subjects who received at least one dose of study medication (i.e. tafenoquine or chloroquine/primaquine) will be included in the Safety and ITT analysis populations.

7.3. Dose Rationale

Based on safety and tolerability data, efficacy data from dose-ranging chemoprophylaxis studies and pharmacokinetic data gathered during Phase I and II, a dose of either 600 mg for one day, or 400 mg for three days has been selected as the dose to evaluate tafenoquine as a single agent for the radical cure of *P. vivax*. The 400 mg dose for 3 days was better tolerated than repeat regimens at higher doses (500 mg or 600 mg for 3 days),

and has demonstrated efficacy for eradication of *P. vivax* hypnozoites. The 600 mg dose for one day, is the maximum single dose of tafenoquine administered to subjects and has demonstrated comparable tolerability, safety and efficacy for eradication of *P. vivax* hypnozoites, as the 400 mg dose for 3 days.

A standard blood schizonticidal dosing regimen of chloroquine (1000 mg chloroquine phosphate for 2 days followed by 500 mg chloroquine phosphate for 1 day) followed by a standard hypnozoite eradication dosing regimen for primaquine (15 mg base per day for 14 days) will be used for the control arm in this study. As the safety of chloroquine for the treatment of malaria is well established, the control arm will be used to allow for subjective comparisons with tafenoquine for the occurrence of ophthalmic and renal effects and to help discern if tafenoquine exacerbates the occurrence and severity of the adverse events that are to be closely monitored during this study (nausea, diarrhea, and vomiting) which are often symptoms of malaria.

7.4. Blinding

This will be an active-control, double-blind, double-dummy study, in which all subjects will receive both active and placebo study medication. Each randomized subject will be allocated the next (i.e. lowest) sequential treatment number and the corresponding medication pack will be assigned. Neither the subject, the Investigator nor the study staff will know which treatment has been allocated as all medication packs will be identical in appearance.

Emergency unblinding will be available via GSK's Registration and Medication Ordering System (RAMOS). To unblind a subject the site should call RAMOS using the usual toll-free number for that country. To identify the subject RAMOS will request the CRF number (subject number) or any of the previously dispensed container numbers for that subject. Detailed guidance will be provided in the study worksheet issued to each site before study start.

Only in the case of an emergency, when knowledge of the investigational product is essential for the clinical management or welfare of the subject, the investigator may unblind a subject's treatment assignment. If the blind is broken for any reason, the investigator must notify USAMRMC and GSK **immediately** of the unblinding incident without revealing the subject's study treatment assignment. In addition, the investigator will record the date and reason for revealing the blinded treatment assignment for that subject in the appropriate CRF.

If a serious adverse event (SAE; as defined in Section 10.2., "Definition of a SAE") is reported to GSK, Global Clinical Safety and Pharmacovigilance (GCSP) staff may unblind the treatment assignment for the individual subject. If an expedited regulatory report to one or more regulatory agencies is required, the report will identify the subject's treatment assignment. When applicable, a copy of the regulatory report may be sent to investigators in accordance with relevant regulations, GSK policy, or both.

Where the treatment blind is broken for any subject, treatment will be withdrawn for that subject.

7.5. Treatment Assignment

Subjects will be assigned to study treatment in accordance with the randomization schedule. Separate randomization lists will be generated for Cohorts 1 and 2 by a GSK statistician using the Coding Memo System (GSK randomization software package) to allocate subjects in a 2:1 ratio to the tafenoquine treatment group, using blocking. GSK will hold the master randomization list for each Cohort and the block size will remain confidential.

Subjects within Cohort 1 will be randomized to one of the two treatment groups:

1. Tafenoquine (400mg base) and chloroquine placebo x 3d, followed by primaquine placebo for 14 days.
2. Chloroquine (1000 mg chloroquine phosphate) and tafenoquine placebo x 2 day, followed by chloroquine (500 mg chloroquine phosphate) and tafenoquine placebo x 1day, followed by primaquine, 15 mg/day for 14 days.

Subjects within Cohort 2 will be randomized to one of the two treatment groups:

1. Tafenoquine (600 mg base) and chloroquine placebo x 1d, chloroquine placebo x 2 days, followed by primaquine placebo for 14 days.
2. Chloroquine (1000 mg chloroquine phosphate) and tafenoquine placebo x 1 day, followed by chloroquine (1000 mg chloroquine phosphate) x 1 day, followed by chloroquine (500 mg chloroquine phosphate) x 1 day, followed by primaquine, 15 mg/day for 14 days.

Each randomized subject will be allocated the next (i.e. lowest) sequential treatment number. Once a subject number has been allocated, it will not be reallocated to any other subject.

7.6. Packaging and Labeling

The study medication will be provided in white opaque high density polyethylene (HDPE) bottles with white opaque, child resistant closures with coated polyester film bonded aluminum foil inner seal.

Each bottle will contain sufficient medication for treatment and additional overage to allow for any re-dosing and other losses.

The contents of the label will be in accordance with all applicable regulatory requirements.

7.7. Handling and Storage

Investigational product must be administered according to procedures described herein. Only subjects enrolled in the study may be administered product, in accordance with all applicable regulatory requirements. Only authorized site staff may administer investigational product. All investigational products must be stored in a secure area with

access limited to the investigator and authorized site staff and under physical conditions that are consistent with investigational product-specific requirements.

The study medication should be stored at room temperature (between 15° to 30° C) under secure conditions.

7.8. Product Accountability

The investigator is responsible for investigational product accountability, reconciliation, and record maintenance. In accordance with all applicable regulatory requirements, the investigator or designated site staff must maintain investigational product accountability records throughout the course of the study. This person(s) will document the amount of investigational product received from GSK, and the amounts administered to subjects.

The investigator or designee, upon receipt of the investigational product supplies, will conduct an inventory and sign both copies of the forms provided by GSK. One copy should be forwarded as instructed in the form and the other copy must be retained for the investigator's study records.

The treatment bottle will be the accountability unit.

The investigator, or designee, will record the administration of the investigational product to subjects and any subsequent returns or losses of drug supply. These records will be made available to clinical monitoring personnel. An investigational product supply inspection for inventory purposes and assurance of proper storage will be conducted at regular intervals throughout the clinical investigation. Any significant discrepancy and/or deficiency is to be recorded, reported to USAMRMC and GSK, and a plan for resolution is to be documented.

7.9. Assessment of Compliance

Study drug will be administered under supervision during the treatment phase of this study. Compliance will be recorded in the CRF at each visit (date and time of administration of study medication).

7.10. Treatment of Investigational Product Overdose

There is no experience of overdose with tafenoquine. Overdose of study medication is unlikely to occur in this study, as dosing will be supervised at each treatment visit. In the event that overdose does occur, treatment will be according to the clinical judgement of the investigator.

7.11. Occupational Safety

Investigational product is not expected to pose significant occupational safety risk to site staff under normal conditions of use and administration. A Material Safety Data Sheet (MSDS) describing occupational hazards and recommended handling precautions either

will be provided to the investigator, where this is required by local laws, or is available upon request from GSK.

8. CONCOMITANT MEDICATIONS AND NON-DRUG THERAPIES

8.1. Permitted Medications

The use of concomitant medications should be limited to those essential for the care of the subject. Other medication which the subject takes regularly, i.e., for control of chronic conditions, should be continued as directed by the subject's physician.

All concomitant medications taken during the study will be recorded in the CRF with indication, dose information, and dates of administration.

8.2. Prohibited Medications

Subjects taking concomitant medications likely to affect renal or ophthalmic function should not be entered into the study. Subjects who require such medications during the study should be withdrawn from treatment and given appropriate anti-malarial treatment according to [redacted] guidelines.

In addition, concomitant medications that are known to be metabolized primarily by the cytochrome P450 isoforms 3A4/5 and 2C9 and whose therapeutic effect occurs within a narrow plasma concentration range (e.g. warfarin, ketoconazole) should not be taken during treatment with study medication. In view of the long half-life of tafenoquine (2-3 weeks) it is further recommended that co-administration of drugs metabolized by cytochrome P450 isoforms 3A4/5 and 2C9 is avoided for a period of 5 half-lives (approximately 3 months) after completion of study medication. Subjects who require such medications during the treatment phase of the study should be withdrawn from treatment and given appropriate anti-malarial treatment according to [redacted] guidelines.

Subjects will be discouraged from taking any prescription medication during the study and will be asked to contact the study clinic before commencing any such medication. Subjects may not take any herbal medication during the study period. Details of all concomitant medications will be entered into the CRF.

9. SUBJECT COMPLETION AND WITHDRAWAL

9.1. Subject Completion

A subject will be considered to have completed the study if they received all study medication, and attended all scheduled study visits up to and including the follow-up at Day 120.

9.2. Subject Withdrawal

A withdrawal is any subject who enters the study (i.e., gives informed consent and is randomized to study treatment), but does not complete the study (whether or not the subject received study medication).

When a subject is withdrawn, the investigator should carry out all the assessments that would have been carried out at the next scheduled visit (unless the subject is lost to follow-up). The Study Conclusion Page of the CRF must be completed and the study medication records should be brought up to date as far as possible.

Subjects withdrawing from the study should attend for a follow-up visit 90 days after enrollment in the study for the monitoring of adverse events and changes in concomitant medication. Every effort will be made to follow-up subjects who withdrew due to drug related adverse events in order to determine the final outcome. This must then be recorded in the CRF and reported to GSK. All randomized subjects who received at least one dose of study medication (i.e. tafenoquine or chloroquine/primaquine) will be included in the Safety and ITT analysis populations.

Subjects may be withdrawn from the study for any one of the following reasons; details must be documented in the CRF:

- Withdrawal of consent at any stage
- Presence of *P. vivax* parasitemia at Day 7 or if *P. vivax* parasitemia recurs
- Adverse Experience
- Protocol violation/deviation (including non-compliance)
- Lost to Follow-up
- Termination by USAMRMC and GSK
- Other

9.2.1. Subject Withdrawal from Study

Subject withdrawal from the study is defined as permanent discontinuation of study medication or attendance at study visits. The subject number and study medication will not be reallocated to any other subject. All randomized subjects who received at least one dose of study medication (i.e. tafenoquine or chloroquine/primaquine) will be included in the Safety and ITT analysis populations.

The reason for withdrawal must be recorded in the subject's medical record and in the CRF.

If the subject has any clinically significant renal or ophthalmic findings, they will be followed up, as appropriate, to assess reversibility of any findings.

- If the subject is prematurely withdrawn from the study due to an adverse event or serious adverse event, the procedures stated in Section 10 must be followed.

9.2.2. Subject Withdrawal from Investigational Product

Subject withdrawal from investigational product is defined as permanent discontinuation of study medication. The subject number and study medication will not be reallocated to any other subject.

The reason for investigational product withdrawal must be recorded in the subject's medical record and in the CRF. Reasons may include, but are not limited to:

- Adverse event
- Consent withdrawn
- Protocol violation

If the subject is withdrawn from the study medication due to an adverse event or serious adverse event, the procedures stated in Section 10 must be followed.

10. ADVERSE EVENTS (AE) AND SERIOUS ADVERSE EVENTS (SAE)

The investigator is responsible for the detection and documentation of events meeting the criteria and definition of an AE or SAE as provided in this protocol. During the study, when there is a safety evaluation, the investigator or site staff will be responsible for detecting AEs and SAEs, as detailed in this section of the protocol.

10.1. Definition of an AE

Any untoward medical occurrence in a subject or clinical investigation subject, temporally associated with the use of a medicinal product, whether or not considered related to the medicinal product.

An AE can therefore be any unfavorable and unintended sign (including an abnormal laboratory finding), symptom, or disease (new or exacerbated) temporally associated with the use of a medicinal product. For marketed medicinal products, this also includes failure to produce expected benefits (i.e. lack of efficacy), abuse or misuse.

Examples of an AE **includes**:

- Significant or unexpected worsening or exacerbation of the condition/indication under study. See Section 10.3., "Lack of Efficacy", for additional information.
- Exacerbation of a chronic or intermittent pre-existing condition including either an increase in frequency and/or intensity of the condition.
- New conditions detected or diagnosed after investigational product administration even though it may have been present prior to the start of the study.
- Signs, symptoms, or the clinical sequelae of a suspected interaction.

- Signs, symptoms, or the clinical sequelae of a suspected overdose of either investigational product or a concurrent medication (overdose per se should not be reported as an AE/SAE).
- Significant failure of expected pharmacological or biological action. See Section 10.3, “Lack of Efficacy” for additional information.

Examples of an AE **does not include** a/an:

- Medical or surgical procedure (e.g., endoscopy, appendectomy); the condition that leads to the procedure is an AE.
- Situations where an untoward medical occurrence did not occur (social and/or convenience admission to a hospital).
- Anticipated day-to-day fluctuations of pre-existing disease(s) or condition(s) present or detected at the start of the study that do not worsen.
- The disease/disorder being studied, or expected progression, signs, or symptoms of the disease/disorder being studied, unless more severe than expected for the subject’s condition.

AEs may include pre- or post-treatment events that occur as a result of protocol-mandated procedures (i.e., invasive procedures, modification of subject’s previous therapeutic regimen).

10.2. Definition of a SAE

A serious adverse event is any untoward medical occurrence that, at any dose:

a) results in death.

b) is life-threatening.

NOTE: The term 'life-threatening' in the definition of 'serious' refers to an event in which the subject was at risk of death at the time of the event. It does not refer to an event, which hypothetically might have caused death, if it were more severe.

c) requires hospitalization or prolongation of existing hospitalization.

NOTE: In general, hospitalization signifies that the subject has been detained (usually involving at least an overnight stay) at the hospital or emergency ward for observation and/or treatment that would not have been appropriate in the physician’s office or out-patient setting. Complications that occur during hospitalization are AEs. If a complication prolongs hospitalization or fulfills any other serious criteria, the event is serious. When in doubt as to whether “hospitalization” occurred or was necessary, the AE should be considered serious.

Hospitalization for elective treatment of a pre-existing condition that did not worsen from baseline is not considered an AE.

d) results in disability/incapacity, or

NOTE: The term disability means a substantial disruption of a person's ability to conduct normal life functions. This definition is not intended to include experiences of relatively minor medical significance such as uncomplicated headache, nausea, vomiting, diarrhea, influenza, and accidental trauma (e.g. sprained ankle) which may interfere or prevent everyday life functions but do not constitute a substantial disruption.

e) is a congenital anomaly/birth defect.

f) Medical or scientific judgement should be exercised in deciding whether reporting is appropriate in other situations, such as important medical events that may not be immediately life-threatening or result in death or hospitalization but may jeopardize the subject or may require medical or surgical intervention to prevent one of the other outcomes listed in the above definition. These should also be considered serious. Examples of such events are invasive or malignant cancers, intensive treatment in an emergency room or at home for allergic bronchospasm, blood dyscrasias or convulsions that do not result in hospitalization, or development of drug dependency or drug abuse.

g) The following will also be classified as serious adverse events and will be subject to close monitoring by GCSP:

- A confirmed serum creatinine increase from baseline of 26.6 µmol/L (0.3 mg/dL) that is sustained for ≥ 14 days.
- Retinal findings (as described in Section 6.2.5) or another significant ophthalmic event during the study.
- Methemoglobin concentration of ≥20%
- Recurrent (> 4 times within one day) or intractable vomiting
- Severe diarrhea resulting in loss of intravascular volume judged by the investigator to be sufficiently severe to warrant the initiation or increase in rate of administration of intravenous replacement fluids after onset of diarrhea
- Nausea resulting in no significant oral intake, requiring intravenous fluids

10.3. Lack of Efficacy

"Lack of efficacy" per se will not be reported as an AE. The signs and symptoms or clinical sequelae resulting from lack of efficacy will be reported if they fulfill the AE or SAE definition (including clarifications).

10.4. Clinical Laboratory Abnormalities and Other Abnormal Assessments as AEs and SAEs

Abnormal laboratory findings (e.g., clinical chemistry, hematology, urinalysis) that are judged by the investigator as **clinically significant** will be recorded as AEs or SAEs if they meet the definition of an AE, as defined in Section 10.1. ("Definition of an AE"), or SAE, as defined in Section 10.2. ("Definition of a SAE"). Clinically significant abnormal laboratory findings or other abnormal assessments that are detected during the study or

are present at baseline and significantly worsen following the start of the study will be reported as AEs or SAEs. However, clinically significant abnormal laboratory findings or other abnormal assessments that are associated with the disease being studied, unless judged by the investigator as more severe than expected for the subject's condition, or that are present or detected at the start of the study and do not worsen, will **not** be reported as AEs or SAEs.

The investigator will exercise his or her medical and scientific judgement in deciding whether an abnormal laboratory finding or other abnormal assessment is clinically significant.

The following laboratory and clinical assessments are subject to close monitoring by GCSP and will be upgraded to GSK medically defined serious adverse events for fast-tracking on to the safety database and for rapid follow-up:

- A confirmed serum creatinine increase from baseline of ≥26.6 μmol/L (0.3 mg/dL) that is sustained for ≥ 14 days.
- Retinal findings (as described in Section 6.2.5) or another significant ophthalmic event during the study.

At each clinic visit, details of any adverse events experienced since the last visit, will be recorded. Adverse events of significantly increased methemoglobin, vomiting, diarrhea and nausea will be subject to close safety monitoring. Any of these events that are assessed by the investigator as severe, will be upgraded to GSK medically defined serious for fast-tracking on to the safety database and for rapid follow-up. The criteria for seriousness are:

- Methemoglobin concentration of ≥20%
- Recurrent (> 4 times within one day) or intractable vomiting
- Severe diarrhea resulting in loss of intravascular volume judged by the investigator to be sufficiently severe to warrant the initiation or increase in rate of administration of intravenous replacement fluids after onset of diarrhea
- Nausea resulting in no significant oral intake, requiring intravenous fluids

If ≥ 5 subjects have the above following same drug related AE (based on body systems) of severe intensity, enrollment of further subjects will be put on hold until the data can be reviewed by an IDMC for recommendation on whether to continue enrolling into the study.

10.5. Time Period, Frequency, and Method of Detecting AEs and SAEs

At each visit/assessment, AEs will be actively collected and evaluated by the investigator. AEs not previously documented in the study will be recorded directly into the electronic CRF. The nature of each experience, date and time (where appropriate) of onset, outcome, course (i.e. intermittent or constant), maximum intensity, action taken with respect to dosage and relationship to treatment should be established. Details of changes

to the dosage schedule or any corrective treatment should be recorded in the electronic CRF.

The time period for the collection of SAE's and AE's will begin at first receipt of investigational product until Day 90. For Days 91-120, any SAEs the investigator learns of, including a death, will be reported promptly.

In addition, serious adverse events that are related to study participation (e.g. procedures) or are related to a concomitant medication will be collected and recorded from the time that the subject consents to participate in the study until he/she are discharged.

At each visit or contact with the subject, after the subject has had an opportunity to spontaneously mention any problems, the investigator should inquire about adverse events by asking the following standard questions:

1. "Have you had any (other) medical problems since your last visit/assessment?"
2. "Have you taken any new medicines, other than those given to you in this study, since your last visit/assessment?"

All unresolved serious adverse events observed at the last study visit should be followed by the investigator until they are resolved or stabilized, the subject is lost to follow-up, or the event is otherwise explained. The Investigator should report the follow-up for these serious adverse events

10.6. Recording of AEs and SAEs

Details of AE/SAE's will be entered directly into the electronic CRF. In the case of an SAE further information will be collected on the paper SAE form provided.

When an AE/SAE occurs, it is the responsibility of the investigator to review all documentation (e.g., hospital progress notes, laboratory, and diagnostics reports) relative to the event.

The investigator will attempt to establish a diagnosis of the event based on signs, symptoms, and/or other clinical information. In such cases, the diagnosis should be documented as the AE/SAE and not the individual signs/symptoms.

AEs and subject-completed questionnaires are independent components of the study. Responses to each question in the questionnaires will be treated in accordance with standard scoring and statistical procedures detailed by the scale’s developer. The use of a single question from a multidimensional health survey to designate a cause-effect relationship to an AE is inappropriate.

10.7. Evaluating AEs and SAEs

10.7.1. Assessment of Intensity

The investigator will make an assessment of intensity for each AE and SAE reported during the study. The assessment will be based on the investigator's clinical judgement. The intensity of each AE and SAE recorded in the electronic CRF should be assigned to one of the following categories:

Mild: An event that is easily tolerated by the subject, causing minimal discomfort and not interfering with everyday activities.

Moderate: An event that is sufficiently discomforting to interfere with normal everyday activities.

Severe: An event that prevents normal everyday activities.

An AE that is assessed as severe should not be confused with a SAE. Severity is a category utilized for rating the intensity of an event; and both AEs and SAEs can be assessed as severe. An event is defined as 'serious' when it meets one of the pre-defined outcomes as described in Section 10.2., "Definition of a SAE".

Adverse events of significantly increased methemoglobin concentration, vomiting, diarrhea and nausea will be subject to close safety monitoring. Any of these events that are assessed by the investigator as severe, for example

- Methemoglobin concentration of ≥20%
- Repeated (>4 times within one day) or intractable vomiting
- Severe diarrhea resulting in loss of intravascular volume judged by the investigator to be sufficiently severe to warrant the initiation or increase in rate of administration of intravenous replacement fluids after onset of diarrhea
- Nausea resulting in no significant oral intake, requiring intravenous fluids

will be upgraded to GSK medically defined serious for fast-tracking on to the safety database and for rapid follow-up.

10.7.2. Assessment of Causality

The investigator is obligated to assess the relationship between investigational product and the occurrence of each AE/SAE. The investigator will use clinical judgement to determine the relationship. Alternative causes, such as natural history of the underlying diseases, concomitant therapy, other risk factors, and the temporal relationship of the event to the investigational product will be considered and investigated. The investigator will also consult the CIB/IB and/or Product Information, for marketed products, in the determination of his/her assessment.

There may be situations when an SAE has occurred and the investigator has minimal information to include in the initial report to USAMRMC and GSK. However, it is very important that the investigator always make an assessment of causality for every event prior to transmission of the electronic CRF or paper SAE form to USAMRMC and GSK. The investigator may change his/her opinion of causality in light of follow-up information, amending the electronic CRF and paper SAE form accordingly. The causality assessment is one of the criteria used when determining regulatory reporting requirements.

The investigator will provide the assessment of causality as per instructions on the electronic CRF and paper SAE form.

10.8. Follow-Up of AEs and SAEs

After the initial AE/SAE report, the investigator is required to proactively follow each subject and provide further information to USAMRMC and GSK on the subject's condition.

All AEs and SAEs documented at a previous visit/contact and are designated as ongoing, will be reviewed at subsequent visits/contacts.

All AEs and SAEs will be followed until resolution, until the condition stabilizes, until the event is otherwise explained, or until the subject is lost to follow-up. Once resolved, the appropriate electronic CRF page(s) and paper SAE form will be updated. The investigator will ensure that follow-up includes any supplemental investigations as may be indicated to elucidate the nature and/or causality of the AE or SAE. This may include additional laboratory tests or investigations, histopathological examinations, or consultation with other health care professionals.

USAMRMC and GSK may request that the investigator perform or arrange for the conduct of supplemental measurements and/or evaluations to elucidate as fully as possible the nature and/or causality of the AE or SAE. The investigator is obligated to assist. If a subject dies during participation in the study or during a recognized follow-up period USAMRMC and GSK will be provided with a copy of any post-mortem findings, including histopathology.

New or updated information will be recorded on the originally completed paper "SAE" form and within the electronic CRF, with all changes signed and dated by the investigator. The updated electronic CRF and paper SAE form should be resent to GSK within the time frames outlined in Section 10.9.

10.9. Prompt Reporting of SAEs to USAMRMC and GSK

SAEs will be reported promptly to the U.S. Army Medical Research and Materiel Command (USAMRMC) and GSK as described in Section 10.9.1 once the investigator determines that the event meets the protocol definition of an SAE.

10.9.1. Timeframes for Submitting SAE Reports to USAMRMC and GSK

	Initial SAE Reports		Follow-up Information on a Previously Reported SAE	
Type of SAE	Time Frame	Documents	Time Frame	Documents
All SAEs	24 hrs	1. Paper SAE forms to be faxed to USAMRMC and GSK<br>2) eCRF data to be transferred to GSK (replicated)	24 hrs	1. Updated paper "SAE" form to be faxed to USAMRMC and GSK.<br>2) Updated eCRF data to be transferred to GSK (replicated)

10.9.2. Completion and Transmission of the SAE Reports

10.9.2.1. Completion and Transmission of the SAE Reports to GSK

Once an investigator becomes aware that an SAE has occurred in a study subject, she/he will report the information within 24 hours as outlined in Section 10.9., “Prompt Reporting of SAEs to USAMRMC and GSK”. The paper SAE form and electronic CRF will always be completed as thoroughly as possible with all available details of the event, signed by the investigator (or designee), and forwarded to USAMRMC and GSK within the designated time frames. If the investigator does not have all information regarding an SAE, he/she will not wait to receive additional information before notifying GSK of the event and completing the form. The form will be updated when additional information is received. Follow-up information should be forwarded to GSK as described in section 10.8.

The investigator will always provide an assessment of causality at the time of the initial report as described in Section 10.7.2, “Assessment of Causality”.

Facsimile transmission of the “SAE” CRF is the preferred method to transmit this information to the project contact for SAE receipt. In rare circumstances and in the absence of facsimile equipment, notification by telephone is acceptable, with a copy of the "SAE" CRF sent by overnight mail. Initial notification via the telephone does not replace the need for the investigator to complete and replicate the electronic CRF within the time frames outlined in Section 10.9, “Prompt Reporting of SAEs to USAMRMC and GSK”.

GSK will provide a list of project contacts for SAE receipt, fax numbers, telephone numbers, and mailing addresses.

The following sections of the electronic CRF must accompany the SAE forms that are forwarded to GSK: “Demography”, “Medical History”, “Concomitant Medications”, “Study Medication Records”, and death notification form “Form D” (if applicable).

10.9.2.2. Completion and Transmission of the SAE Reports to USAMRMC

Adverse experiences that are both serious and unexpected will be immediately reported by telephone to the USAMRMC Office of Regulatory Compliance and Quality [redacted] (non-duty hours call [redacted] and send information by facsimile to [redacted] A written report will follow the initial telephone call within 3 working days. Address the written report to the U.S. Army Medical Research and Materiel Command, ATTN: MCMR-RCQ, 504 Scott Street, Fort Detrick, Maryland 21702-5012.

10.10. Regulatory Reporting Requirements For SAEs

The investigator will promptly report all SAEs to USAMRMC and GSK in accordance with the procedures detailed in Section 10.9, "Prompt Reporting of SAEs to USAMRMC and GSK." USAMRMC and GSK have a legal responsibility to notify, as appropriate, both the local regulatory authority and other regulatory agencies about the safety of a product under clinical investigation. Prompt notification of SAEs by the investigator to the appropriate project contact for SAE receipt is essential so that legal obligations and ethical responsibilities towards the safety of other subjects are met.

The investigator, or responsible person according to local requirements, will comply with the applicable local regulatory requirements related to the reporting of SAEs to regulatory authorities and the Institutional Review Board (IRB)/Independent Ethics Committee (IEC).

This protocol has been filed under an Investigational New Drug (IND) application with the US Food and Drug Administration (FDA). A given SAE may qualify as an IND Safety Report if the SAE is both attributable to the investigational product and unexpected. In this case, all investigators filed to the IND (and associated INDs for the same compound) will receive an Expedited Investigator Safety Report (EISR), identical in content to the IND Safety Report submitted to the FDA.

EISRs are prepared according to GSK policy and are forwarded to investigators as necessary. An EISR is prepared for a SAE that is both attributable to investigational product and unexpected. The purpose of the EISR is to fulfill specific regulatory and Good Clinical Practice (GCP) requirements, regarding the product under investigation.

When a site receives from GSK an Initial or Follow-up EISR or other safety information (e.g., revised Clinical Investigator’s Brochure/Investigator’s Brochure), the responsible person according to local requirements is required to promptly notify his or her IRB or IEC.

10.11. Post-study AEs and SAEs

A post-study AE/SAE is defined as any event that occurs outside of the AE/SAE detection period defined in Section 10.5, “Time Period, Frequency, and Method of Detecting AEs and SAEs”, of the protocol.

Investigators are not obligated to actively seek AEs or SAEs in former study participants. However, if the investigator learns of any SAE, including a death, at any time after a subject has been discharged from the study, and he/she considers the event reasonably related to the investigational product, the investigator will promptly notify USAMRMC and GSK.

10.12. SAEs Related to Study Participation

An SAE considered related to study participation (e.g., procedures, invasive tests, a change in existing therapy), even if it occurs during the pre- or post-treatment period, will be reported promptly to USAMRMC and GSK (see Section 10.9, "Prompt Reporting of SAEs to USAMRMC and GSK").

11. DATA ANALYSIS AND STATISTICAL CONSIDERATIONS

11.1. Hypotheses

The null hypothesis for the primary endpoint of the Day 28 cure rate (i.e. the proportion of successes defined as subjects classified as having an 'Adequate Clinical Response' at Day 28 - see Section 3.1) is that a given tafenoquine dose under study (i.e. a single 600 mg dose or 400mg/day for 3 days) is not efficacious in clearing/curing *P. vivax* blood stage infections. The alternative hypothesis is that the tafenoquine dose under study is efficacious in clearing/curing these infections. This hypothesis will be tested separately for the two cohorts receiving the different tafenoquine doses.

It will be concluded that each dose of tafenoquine is efficacious if the lower bound of the one-sided 95% confidence interval for the Day 28 cure rate for tafenoquine subjects in the cohort is no less than 85%.

11.2. Treatment Comparisons of Interest

11.2.1. Primary Comparisons of Interest

No formal comparison between the treatment groups for the primary analysis is to be made for the purpose of drawing conclusions on efficacy, as the study is not sufficiently powered for such a comparison (due to the very high success rates expected in each treatment group). The primary objective of the study relates to assessing the efficacy of the tafenoquine doses alone.

The chloroquine plus primaquine treatment arm is included as a control group only. For the purpose of estimation a 95% CI (1 sided) will also be calculated for the Day 28 cure

rate in this treatment arm for each cohort. The treatment difference and associated 95% CI (2-sided) between tafenoquine and this control group will also be calculated for each cohort.

11.2.2. Other Comparisons of Interest

Secondary efficacy and safety endpoints will be summarized descriptively to allow informal comparisons to be made between treatment groups. No formal hypothesis testing will be applied to these additional endpoints, although interval estimation will be applied to some, as detailed in Section 11.7.2.

The baseline demographic characteristics of subjects will be summarized to assess the comparability of the treatment groups at baseline. If large differences are observed between treatment groups, then the effects of these characteristics will be investigated. No formal hypothesis testing or interval estimation will be applied to baseline or demographic characteristics.

11.3. Interim Analysis

11.3.1. Planned Interim Analysis After Cohort 1 Day 28 Assessment

A planned interim analysis will be performed after all subjects in Cohort 1 have completed the Day 28 assessment. This interim analysis will be based on all efficacy and safety data up to and including the Day 28 assessment. Data will be presented appropriately either as individual subject data listings, or as data summaries by treatment group. The format of these data presentations will be defined in the RAP. All data analyses conducted for this interim analysis will be prepared by an independent Statistical Data Analysis Center to ensure that GSK study related personnel remain blinded to treatment codes until the study is complete. An Independent Data Monitoring Committee (IDMC) will convene to evaluate the efficacy and safety of the tafenoquine dosing regimen (400 mg tafenoquine once per day for 3 days) used in Cohort 1. Only if the results from Cohort 1 meet pre-defined efficacy and safety criteria will enrollment begin for Cohort 2. The efficacy criterion for achieving the primary endpoint is that the lower limit of the one-sided 95% confidence interval is no less than 85%, and for safety that a review of trends in all AEs, tolerability, medical observations, methemoglobin and other lab data for all subjects indicates the dose is safe and well tolerated. The IDMC will review all the available relevant study data and advise on the appropriateness of initiating enrollment for Cohort 2. The data for Cohort 2, together with the Day 60, Day 90, and Day 120 safety and parasitological assessments for Cohort 1, will be reported separately at the end of the study, as described in Sections 11.7,11.8, and 11.9 and will be conducted by GSK.

11.3.2. Ad-hoc Interim Analyses During Enrollment

Additionally, as detailed in Sections 6.2.3, 6.2.5, 6.3.3, and 10.4, an ad-hoc IDMC meeting may be called at any time during enrollment to either cohort, if any of the pre-defined efficacy, renal, ophthalmic or adverse event triggers are reached. The purpose of

this meeting will be to advise on continuation of enrollment. In the event of such a meeting all relevant safety and efficacy data will be summarized by an independent Statistical Data Analysis Center and presented to the IDMC. The specification of these summaries will be agreed with the IDMC members in advance. All GSK study related personnel will remain blinded to the treatment codes until the planned analysis for each cohort is complete. The probability of reaching any of the safety triggers for an IDMC meeting during the study are discussed in Section 11.4.2.

Lack of efficacy will be reviewed after the first 21 subjects have been enrolled in each cohort. It is believed that 21 subjects will be sufficient to provide a strong indication of lack of efficacy, if it truly exists. Subjects will be hospitalized and closely monitored during this time period and would receive rapid treatment upon relapse. For both cohorts if 4 or more ETFs (as defined in Section 3.1) are observed in the first 21 subjects enrolled (as detailed in Section 6.3.3), enrollment will be suspended and the SDAC will unblind the data. All GSK personnel are to remain blinded to the data. If the early treatment (ET) response rate in the tafenoquine arm is found to be ≥70% enrollment will be re-initiated. If the ET response rate in the tafenoquine arm is found to be below 70% an ad-hoc IDMC meeting will be called.

From 21 subjects, with a 2:1 randomization, it is anticipated on unblinding that there could realistically be as few as 12, or as many as 16 evaluable tafenoquine treated subjects. The data presented shows the resultant tafenoquine ET response rates, as well as the lower limit of the one-sided 95% confidence interval, if either 3 or all 4 ETFs are found on unblinding, to be on the tafenoquine arm:

No of tafenoquine subjects	No of tafenoquine ETFs	ET response rate	Lower limit of one-sided 95% confidence interval
12	3	75.0%	47.3%
	4	66.7%	39.1%
13	3	76.9%	50.5%
	4	69.2%	42.7%
14	3	78.6%	53.4%
	4	71.4%	46.0%
15	3	80.0%	56.0%
	4	73.3%	48.9%
16	3	81.3%	58.3%
	4	75.0%	51.6%

Thus it is notable that due to the limited quantity of data and associated high variability, an ET response rate of ≥70% (hence not resulting in the calling of an ad-hoc IDMC meeting), could realistically yield an associated 95% confidence interval with a lower limit as low as 46%.

11.4. Sample Size Considerations

Unless enrollment to either cohort is terminated at the recommendation of an IDMC, approximately 140 subjects will be enrolled, 70 to Cohort 1 followed by 70 to Cohort 2. From this sample size, the aim is to yield 120 evaluable subjects in total, with 60 subjects for each cohort. A 2:1 randomization ratio will be used to obtain 40 evaluable subjects in each of the 2 cohorts for the tafenoquine arm and 20 evaluable subjects in each of the 2 cohorts for the comparator arm, (allowing approximately a 14% non-evaluability rate).

11.4.1. Sample Size Assumptions

The primary endpoint is the Day 28 cure rate for *P. vivax* (as defined in Section 3.1). If the lower limit of the one-sided 95% confidence interval for the proportion of tafenoquine subjects in the cohort who are considered successes (i.e. classified as having an 'Adequate Clinical Response' at Day 28) is less than 85%, then this dose would not be considered efficacious. This pre-specified lower limit of 85% has been based on the high expected cure rates (close to 100%) for both tafenoquine doses and is as required given the high cure rates available from existing therapies.

Thus cure rates of 100% for the primary endpoint have been assumed for both doses of tafenoquine. However it is of interest to establish the impact if tafenoquine failures occur within a cohort. With 40 evaluable tafenoquine subjects within a cohort, the lower limit of the one-sided 95% confidence interval for the Day 28 cure rate ranges from 92.8% for 0 failures to 81.7% for 3 failures.

No of failures[1]	Lower limit of one-sided 95% confidence interval[2]
0	92.8%
1	88.7%
2	85.1%
3	81.7%

1. Out of the 40 evaluable tafenoquine subjects in the cohort
2. Confidence interval based on the proportion of subjects with an 'Adequate Clinical Response' in the tafenoquine group, calculated using the Clopper-Pearson Exact methodology.

The data presented shows that the lower limit of the one-sided 95% confidence interval for the proportion of subjects in the tafenoquine group who are successes on the primary endpoint will be below 85% if three or more failures are observed.

With 40 evaluable tafenoquine subjects, the study has 90% power to show that the lower limit of the one-sided confidence interval is above 85%, if the true success rate for treatment response is 98% or more. (It is anticipated that all subjects in the tafenoquine groups will be successes).

As stated in Section 11.2.1, no formal comparison is to be made between the treatment groups with regard to the primary endpoint, although the treatment difference and associated 95% confidence interval for the Day 28 cure rate between the tafenoquine and control groups will be presented for each cohort.

11.4.2. Sample Size Sensitivity

The robustness and sensitivity of the above sample size should be considered in order to assess the impact of different circumstances on the power of the study.

The power of the analysis for the primary endpoint will vary if the observed success rate is different to that expected.

Given a one-sided 95% confidence interval, a lower bound limit of 85% and 40 evaluable tafenoquine subjects, the data presented shows how the power of the study is affected if the Day 28 cure rate (success rate) ranges between 93% and 99%.

	Success Rate on Primary Endpoint			
	99%	97%	95%	93%
Power	99%	88%	68%	46%

The data presented illustrates that as the success rate decreases, the power of the study diminishes rapidly.

Additionally the number of failures that are allowable for the lower limit of the one-sided 95% confidence interval to still be at least 85% varies if the proportion of evaluable subjects differs to that expected. The assumed proportion has been based on previous experience of conducting a study at one center and with the requirement for subjects to remain in the local area during the follow-up period.

The data presented shows the effect on the lower limit of the one-sided 95% confidence interval for the Day 28 cure rate:

	Total Number of Evaluable tafenoquine Subjects		
No of failures	44	39	36
0	93.4%	92.6	92.0%
1	89.7%	88.4	87.5%
2	86.4%	84.7	83.5%
3	83.3%	81.3	79.9%

Thus if only 39 evaluable tafenoquine subjects are obtained, then the lower limit of the confidence interval will fall below 85% if 2 failures are observed.

Safety

It is important to also understand the sensitivity of the study to detect the pre-specified renal, ophthalmic and safety limits that would result in suspension of enrollment and trigger an ad-hoc IDMC meeting. For example, after 70 subjects have been enrolled to a cohort (and assuming all subjects are eligible for the Safety population, there is approximately a 90% probability of observing the following within each cohort in the study:

- A sustained (≥ 14 days) serum creatinine increase of ≥26.6 μmol/L (0.3 mg/dL) in ≥8 subjects, if the underlying rate of glomerular filtration rate decrease across subjects in both treatment groups, is 1 in 6 (16.25%)
- Retinal changes from baseline that cause clinical concern are seen in ≥2 subjects, if the underlying rate of ophthalmic injury across subjects in both treatment groups, is 1 in 18 (5.45%).
- The same pre-specified drug related AE in ≥5 subjects, (based on body systems) of severe intensity, if the underlying rate of each of these severe AEs across subjects in both treatment groups, is 1 in 9 (11.1%).

Note that the above probabilities are based on thresholds that would trigger an ad-hoc IDMC meeting after 70 subjects have been enrolled. However the study will have a 90% probability of triggering an IDMC meeting prior to enrollment of 70 subjects, if the underlying rates are actually higher than these.

It is also of interest to note that the study still has a 25% probability of triggering an IDMC meeting if the following lower underlying rates are observed after all 70 subjects have been enrolled in each cohort:

- A sustained (≥ 14 days) serum creatinine increase of ≥26.6 μmol/L (0.3 mg/dL) in ≥8 subjects, if the underlying rate of GFR decrease across subjects in both treatment groups, is 1 in 12 (8.58%)
- Retinal changes from baseline that cause clinical concern in ≥2 subjects, if the underlying rate of ophthalmic injury across subjects in both treatment groups, is 1 in 73 (1.37%).
- The same pre-specified drug related AE in ≥5 subjects, (based on body systems) of severe intensity, if the underlying rate of each of these severe AEs across subjects in both treatment groups, is 1 in 21 (4.83%).

11.4.3. Sample Size Re-estimation

No sample size re-estimation is planned in this study.

11.5. Analysis Populations

Seven populations are defined for the analysis of the data to be collected as part of this study. All decisions on eligibility for inclusion in these populations will be made prior to unblinding of a cohort.

Safety Population: all randomized subjects who received at least one dose of study medication (i.e. tafenoquine or chloroquine/primaquine).

Intent to Treat (ITT) Population: all randomized subjects who received at least one dose of study medication (i.e. tafenoquine or chloroquine/primaquine).

Thus for this study, membership of the Safety and ITT populations will be the same.

Per Protocol (PP) Populations:

Day 7 Per Protocol Population: all subjects in the ITT population who attended all required efficacy assessments to Day 7 (including all blood smears and temperature readings, to enable an evaluable efficacy response), who do not violate any inclusion/exclusion criterion that could impact efficacy, have taken all medication and are compliant with the protocol (i.e. do not commit a major violation, as defined in the RAP) up to this time point.

Day 28 Per Protocol Population: all subjects in the Day 7 PP population who subsequently attend all required efficacy assessments to Day 28 (enabling an evaluable efficacy response at this timepoint), have taken all medication and are compliant with the protocol up to this timepoint. Note that subjects who are treatment failures (including withdrawing from treatment due to an AE) or relapse will be withdrawn and will not be required to attend subsequent efficacy assessments to be eligible for this population.

Day 60 Per Protocol Population: all subjects in the Day 28 PP population who have an evaluable efficacy assessment at Day 60 and remain compliant with the protocol to this time point.

Day 90 Per Protocol Population: all subjects in the Day 60 PP population who have an evaluable efficacy assessment at Day 90 and remain compliant with the protocol to this time point.

Day 120 Per Protocol Population: all subjects in the Day 90 PP population who have an evaluable efficacy assessment at Day 120.

The relevant per protocol population will be the primary population for efficacy analyses, with the ITT population used to provide supportive efficacy analyses. The Safety population will be used for all safety analyses.

Note: a subject will be considered to have been compliant with study medication if they have taken all study medication as detailed in the protocol.

Subjects who received the wrong coded study medication will be analyzed according to the medication they received.

11.5.1. Data Sets

It is anticipated that the vast majority of subjects recruited into the study will attend all visits and therefore have efficacy data available at all specified time points. Since as

stated in Section 11.5, the primary analyses for efficacy endpoints will be based on the appropriate per protocol population, which will only contain data for subjects with evaluable efficacy responses, ***observed case*** datasets will be used. Subjects who are treatment failures (including withdrawing from treatment due to an AE) or relapse will be withdrawn from the study and will not be required to have subsequent efficacy assessments. For these subjects, the response from their last efficacy assessment will be carried forward for analyses at subsequent time points. Subjects who withdraw for other reasons (including loss to follow-up and protocol deviation) will be omitted from this dataset from the time of withdrawal.

Other datasets will be derived and analyzed to assess the robustness of the primary analyses and also for the analysis of the secondary endpoints. For details, see Section 11.7.3.

11.6. General Considerations for Data Analysis

11.6.1. Withdrawal

The number and percentage of subjects withdrawing early from the study, for all reasons and for each individual reason, together with the number of completers, will be tabulated.

11.6.2. Missing Data

As subjects will be required to remain in the local area up to the Day 90 follow-up assessment, it is not anticipated that many problems will be encountered with missing data through to this time point. Additionally, primary analyses for all efficacy endpoints will be based only on subjects with evaluable efficacy data. Details of handling missing data for supportive analyses based on the ITT population, are given in Section 11.5.1. Further details of how to deal with other missing data, such as dates, will be provided in the RAP.

11.6.3. Derived and Transformed Data

The Parasite/Gametocyte Clearance Time (PCT/GCT) for a subject is the time (in half-days) from initiation of treatment until a subject becomes negative for *P. vivax* parasites/gametocytes.

Serial blood smears, conducted every 12 hours (± 2 hours) after the baseline smear to detect the presence of *P. vivax* parasites and gametocytes (Days 1, 2, 3, 4, 5, 6, and 7) will be utilized to determine the time to clearance (date and time of first blood smear testing negative for *P. vivax* parasites/gametocytes) – (date and time of first treatment)+1. Parasites and gametocytes will be considered cleared if 2 consecutive blood smears are negative.

Subjects whose *P.vivax* parasites/gametocytes do not clear by Day 7 will be censored at the time of their last available blood smear. Censored subjects will not be included in the calculation of summary statistics for PCT/GCT.

Body temperature, measured every 12 hours (± 2 hours) after the baseline measurement through Day 7 will be used to determine the Fever Clearance Time (FCT), defined as the time (in half-days) from initiation of treatment until a subject's temperature decreases to 37.2 °C and remains at or below that level for a minimum of 24 hours:

(date and time of first temperature reading of 37.2 °C or below, that is sustained for at least 24 hours) – (date and time of first treatment)+1.

Subjects whose fever has not cleared by Day 7 (i.e. dropped below 37.2 °C for at least 24 hours) will be censored at the time of their last available temperature and subjects without fever at study entry will be censored at baseline. Censored subjects will not be included in the primary analysis for calculation of summary statistics for fever clearance time.

11.6.4. Assessment Windows

Assessment windows will be defined in the RAP prior to the unblinding of the study.

11.6.5. Other Issues

11.6.5.1. Multiplicity

There is a requirement to demonstrate efficacy in Cohort 1 prior to beginning enrollment to Cohort 2 (as defined in Section 11.3). There is also an early efficacy criterion (defined in Section 6.3.3) for both cohorts that the tafenoquine doses must reach in order for enrollment to continue without triggering an ad-hoc IDMC meeting. Thus since tafenoquine efficacy is required to meet each of the defined criteria and there is only one primary analysis for each Cohort for the efficacy endpoints, there will be no formal adjustment for multiplicity in this study

11.6.5.2. Premature Discontinuation of Study

Should the study be prematurely discontinued for any reason, all outstanding data will be summarized as appropriate.

11.6.5.3. Deviations from the Analysis Plan

Any deviations from the analysis planned as part of this protocol will be documented in the RAP, prior to the unblinding of the Cohort 1.

11.7. Efficacy Analyses

11.7.1. Primary Analysis

The primary endpoint (defined fully in Section 3.1) is the Day 28 cure rate on tafenoquine.

Subjects will have a blood smear taken at screening and at time points up to the Day 28 follow-up and will be classified as cured at Day 28 if they meet the criteria for evaluability and have an 'Adequate Clinical Response' (as defined in Section 3.1). Subjects who are evaluable, but do not have an 'Adequate Clinical Response' will be considered failures for the primary analysis, while subjects who are not evaluable will be excluded from it. This primary analysis will be based on a confidence interval for the proportion of subjects in the tafenoquine group in the ***Day 28 per protocol*** population, who are cured at Day 28.

A one-sided 95% confidence interval for the proportion of successes will be calculated using the Clopper-Pearson Exact method [Clopper,1934]. This method will be used to calculate any confidence intervals for tafenoquine and comparator subjects required for primary and secondary analyses. If the lower limit of this one-sided 95% confidence interval is no lower than 85%, it will be concluded that the tafenoquine dose is efficacious in clearing/curing P.vivax blood stage infections.

A one-sided 95% confidence interval for the Day 28 cure rate in the comparator group will also be calculated. However this is for estimation purposes only and will not be used to draw conclusions regarding efficacy of this treatment.

Although no formal comparisons between the treatment groups with regard to the primary endpoint are to be used in order to draw conclusions of relative efficacy, the difference in the Day 28 cures rate and associated 95% confidence interval will be calculated for each cohort.

11.7.2. Other Analyses

No formal comparisons are to be made between the treatment groups for any of the secondary endpoints and no formal hypothesis testing will be carried out. Various data summaries (i.e. tabulations and listings) will be produced, with categorical (and binary) data summarized by counts and percentages in each category. Continuous data will be summarized using means, standard deviations, medians, minima and maxima.

In addition, for the secondary efficacy endpoint of the proportion of subjects without relapse of *P.vivax* at 2 months (Day 60), 3 months (Day 90) and at 4 months (Day 120), one-sided 95% confidence intervals will be calculated for each treatment group and for both timepoints. These are for estimation purposes only (and will be calculated using the Clopper Pearson 'Exact' methodology).

For the remaining secondary efficacy endpoints, (parasite, gametocyte and fever clearance times), frequency tabulations will be produced showing numbers cleared by each sampling time point. For these endpoints and for secondary safety endpoints, descriptive summary statistics will be calculated in order to allow informal comparisons across treatment groups to be made.

Analyses to be carried out for secondary pharmacokinetic endpoints are detailed in Section 11.9.1.

11.7.3. Robustness Analyses

The robustness of the principal efficacy analyses to the method used to handle missing data will be assessed by repeating the analysis using the ***intent to treat*** (ITT) population (defined in Section 11.5). This population will provide a worst case analysis:

- for the primary endpoint, this analysis will include data for all subjects in the ITT population. Subjects who were not included in the principal analysis using the ***per protocol*** population due to having a missing efficacy response will be considered to be failures in this analysis.

  This also applies for the secondary endpoint of prevention of *P.vivax* relapse for 2, 3 and 4 months.

- for the secondary endpoints of PCT, GCT and FCT, censored observations (as defined in Section 11.6.3) will be included in the analysis for calculation of summary statistics, using appropriate survival analysis techniques (e.g. Kaplan Meier survival function). Frequency tabulations for the number of subjects cleared at each sampling time point will also be repeated for the ITT population.

11.8. Safety Analyses

All safety reporting will be based on the safety population and will be analyzed descriptively.

11.8.1. Extent of Exposure

The extent of exposure to study medication will be defined as:

Date of last dose of active study medication – date of first dose of study medication + 1.

11.8.2. Adverse Events

Adverse Event (AE) reporting will be performed using the MedDRA (Medical Dictionaries for Regulatory Activities) coding system. Each AE coded using the MedDRA system can be associated with more than one system organ class (SOC). However, for reporting purposes, an AE will be associated with the primary system organ class only.

Counting of adverse events will be based on the number of subjects – not the number of AEs. For example, if a subject reports the same AE on three occasions within a time interval, that AE will only be counted once. Subjects reporting more than one AE in a system organ class will only be counted once in the system organ class total. All AEs occurring whilst a subject is receiving study medication, or during the 90 day follow-up period, will be reported.

Summary tables to compare the AE profiles of the two treatment groups will be produced for the Safety population (as defined in Section 11.5) for each cohort.

11.8.3. Clinical Laboratory Evaluations

In addition to the endpoints described previously, clinical laboratory data (clinical chemistry, hematology and urinalysis) will be evaluated by tabulating the number of subjects in each treatment group with values outside normal ranges and pre-determined clinically important ranges at each assessment. The number of subjects with clinically significant changes from baseline to the timepoints at which data are collected (which include days 3, 7, 14, 21, 28 and 90 will also be summarized. (The laboratory ranges will be defined in the RAP, prior to the unblinding of the study).

11.8.4. Renal Data Analyses

As detailed in Section 6.2.3, if a sustained (≥ 14 days) serum creatinine increase of ≥26.6 μmol/L (0.3 mg/dL) is confirmed in ≥8 subjects in a cohort, enrollment of further subjects will be put on hold until the data can be reviewed by an IDMC. As these data are of particular interest in establishing the safety of the tafenoquine dosings, appropriate summary tabulations will be provided for Cohort 1 (at the interim analysis) and Cohort 2 (at the end of the study), regardless of whether these data are reviewed by an IDMC. Full details will be provided in the RAP.

11.8.5. Ophthalmic Data Analyses

As detailed in Section 6.2.5, ophthalmic safety will be assessed in all subjects using tests at baseline and at Day 28 and Day 90 visits. If at any time point, retinal changes from baseline that cause clinical concern (as defined in Section 6.2.5) are seen, it will be upgraded to GSK medically defined serious for fast-tracking on to the safety database and for rapid follow-up. If retinal changes from baseline that cause clinical concern are seen in ≥2 subjects enrollment of further subjects will be put on hold until the data can be reviewed by an IDMC. Appropriate summary tabulations will be provided for Cohort 1 (at the interim analysis) and Cohort 2 (at the end of the study). Full details will be provided in the RAP.

11.8.6. Methemoglobin, Vomiting, Diarrhea and Nausea AEs

As detailed in Section 10.4, adverse events of significantly increased methemoglobin concentration, vomiting, diarrhea and nausea will be subject to close safety monitoring. Any of these events that are assessed by the investigator as severe, will be upgraded to GSK medically defined serious for fast-tracking on to the safety database and for rapid follow-up. If ≥ 5 subjects have the above severe drug related AE's (based on body system) enrollment of further subjects will be put on hold until the data can be reviewed by an IDMC. Appropriate summary tabulations will be provided for these AEs for Cohort 1 (at the interim analysis) and Cohort 2 (at the end of the study). Full details will be provided in the RAP.

11.9. Clinical Pharmacology Data Analyses

11.9.1. Pharmacokinetic Analyses

Plasma concentration-time data for tafenoquine will be tabulated for each subject. Population pharmacokinetic methods will be performed using software such as NONMEM or other currently acceptable methods to assess the pharmacokinetics of tafenoquine, if data are appropriate. To support the population pharmacokinetic analysis, the data from this study may be combined with data from other studies. Mean population pharmacokinetic parameters will be assessed taking into account demographic variables (such as age, weight, gender), and concomitant medications. If data permit, the relationship between tafenoquine concentrations and selected adverse events will be explored.

Pharmacokinetic analysis of tafenoquine data will be the responsibility of the Clinical Pharmacokinetics, Modelling & Simulation Department, CPDM, GlaxoSmithKline. All pharmacokinetic data will be stored in the Archives, GlaxoSmithKline, Research and Development.

12. STUDY ADMINISTRATION

12.1. Regulatory and Ethical Considerations

12.1.1. Regulatory Authority Approval

GSK will obtain approval to conduct the study from the appropriate regulatory agency in accordance with any applicable country-specific regulatory requirements prior to a site initiating the study in that country.

12.1.2. Ethical Conduct of the Study and Ethics Approval

This study will be conducted in accordance with "good clinical practice" (GCP) and all applicable regulatory requirements, including, where applicable, the 1996 version of the Declaration of Helsinki.

The investigator (or USAMRMC and GSK, where applicable) is responsible for ensuring that this protocol, the site's informed consent form, and any other information that will be presented to potential subjects (e.g., advertisements or information that supports or supplements the informed consent) are reviewed and approved by the appropriate IEC/IRBs and [redacted] The investigator agrees to allow the IEC/IRB and [redacted] direct access to all relevant documents. The IEC/IRB must be constituted in accordance with all applicable regulatory requirements. GSK will provide the investigator with relevant document(s)/data that are needed for IEC/IRB and [redacted] review and approval of the study. Before investigational product(s) and CRFs can be shipped to the site, GSK must receive copies of the IEC/IRB and [redacted] approval, the approved informed consent form, and any other information that the IEC/IRB and [redacted] has approved for presentation to potential subjects.

For all modifications to the protocol, the informed consent form, or any other information that the IEC/IRB and [redacted] have approved for presentation to potential subjects, the investigator is responsible for ensuring the IEC/IRB and [redacted] reviews and approves, where applicable, these amended documents prior to initiation. The investigator must follow all applicable regulatory requirements pertaining to the use of an amended informed consent form including obtaining IEC/IRB and [redacted] approval of the amended form before new subjects consent to take part in the study using this version of the form. Copies of the IEC/IRB and [redacted] approval of the amended informed consent form/other information and the approved amended informed consent form/other information must be forwarded to GSK promptly.

12.1.3. Informed Consent

Informed consent will be obtained by the Investigator or designee before the subject can participate in the study. The contents and process of obtaining informed consent will be in accordance with all applicable regulatory requirements.

The informed consent process will be initated after evaluation and diagnosis of subjects presenting to the [redacted] The consent process will be initiated by a [redacted] study nurse or investigator in a private, one on one conversation. Each potential subject must be adequately informed of the aims, methods, anticipated benefits and potential hazards of the study and the discomfort it may entail. The subject will be informed that they are at liberty to abstain from participation in the study and that if they choose not to participate they will receive the standard of care antimalarial medication and will be discharged when medically warranted. A subject will also be informed that they may discontinue participation at any time without penalty or loss of benefits to which the subject is otherwise entitled. Transportation back home (for [redacted] residents) will be provided free of charge with financial support from the Tropical Medicine Trust Fund. The subject will be instructed to take time to read the information in the consent form carefully, to discuss it with friends, relatives and their personal doctor, and to take time to decide whether or not they wish to participate. The subject will be encouraged to ask questions if there is anything that is not clear to them. The informed consent should be freely given by the subject, preferably in writing.

12.1.4. Investigator Reporting Requirements

As indicated in Section 10.10, the investigator (or USAMRMC and GSK, where applicable) is responsible for reporting SAEs to the IEC/IRB, in accordance with all applicable regulations. Furthermore, the investigator may be required to provide periodic safety updates on the conduct of the study at his or her site and notification of study closure to the IEC/IRB. Such periodic safety updates and notifications are the responsibility of the investigator and not of GSK.

12.2. Medical Monitor

A medical monitor will be assigned to this study. The name and curriculum vitae of the medical monitor will be provided. This individual will be a qualified physician, other

than the Principal Investigator, who is not associated with this particular protocol, and will be able to provide medical care to research subjects for conditions that may arise during the conduct of the study, and who will monitor the subjects during the conduct of the study. The medical monitor is required to review all serious and unexpected adverse events (per ICH definitions) associated with the protocol and provide an unbiased written report of the event within 10 calendar days of the initial report to the [redacted] At a minimum, the medical monitor should comment on the outcomes of the adverse event (AE) and relationship of the AE to the study medication. The medical monitor should also indicate whether he/she concurs with the details of the report provided by the study investigator. The Medical Monitor is also required to submit AE findings to the [redacted] the Sponsor's Representative will relay these reports to the [redacted]

12.3. Study Monitoring

Monitoring responsibilities for this protocol will be performed by USAMRMC's Quality Assurance Office, and the Division of Microbiology and Infectious Diseases (DMID). In accordance with applicable regulations, GCP, and USAMRMC and GSK procedures, monitors will contact the site prior to the subject enrollment to review the protocol and data collection procedures with site staff. Monitoring visits will be scheduled to take place before entry of the first subject, during the study at appropriate intervals and after the last subject is completed. A Pre-Study/Initiation visit will be conducted with monitors from USAMRMC and DMID. In addition the monitor(s) will periodically contact the site, including conducting on-site visits. The extent, nature and frequency of on-site visits will be based on such considerations as the study objective and/or endpoints, the purpose of the study, study design complexity, and enrollment rate. At least one of these visits will be conducted by USAMRMC Quality Assurance Office and the rest will be conducted by monitors from DMID. The Closeout monitoring visit will be conducted by monitors from both USAMRMC Quality Assurance Office and DMID. Monitoring Reports will be provided to USAMRMC Quality Assurance Office and GlaxoSmithKline after each monitoring visit.

During these contacts, the monitor will:

- Check the progress of the study.
- Review study data collected.
- Conduct source document verification.
- Identify any issues and address their resolution.

This will be done in order to verify that the:

- Data are authentic, accurate, and complete.
- Safety and rights of subjects are being protected.
- Study is conducted in accordance with the currently approved protocol (and any amendments), GCP, and all applicable regulatory requirements.

The investigator agrees to allow the monitor direct access to all relevant documents and to allocate his/her time and the time of his/her staff to the monitor to discuss findings and any relevant issues.

At study closure, monitors will also conduct all activities described in Section 12.5, "Study and Site Closure."

12.4. Quality Assurance

To ensure compliance with GCP and all applicable regulatory requirements, USAMRMC, GSK, and DMID/National Institute of Allergy and Infectious Diseases (NIAID) may conduct a quality assurance audit. Regulatory agencies may also conduct a regulatory inspection of this study. Such audits/inspections can occur at any time during or after completion of the study. If an audit or inspection occurs, the investigator and institution agree to allow the auditor/inspector direct access to all relevant documents and to allocate his/her time and the time of his/her staff to the auditor/inspector to discuss findings and any relevant issues.

12.5. Study and Site Closure

Upon completion of the study, the monitor will confirm that the following activities have been conducted, in conjunction with the investigator or site staff, as appropriate:

- Return of all study data to GSK.
- Data queries.
- Accountability, reconciliation, and arrangements for unused investigational product(s).
- Review of site study records for completeness.
- Return of treatment codes to GSK.
- Shipment of PK samples to assay laboratory(ies).

In addition, USAMRMC and GSK reserve the right to temporarily suspend or prematurely discontinue this study at any time for reasons including, but are not limited to, safety or ethical issues or severe non-compliance. If USAMRMC and GSK determine such action is needed, USAMRMC and GSK will discuss this with the Investigator (including the reasons for taking such action) at that time. When feasible, USAMRMC and GSK will provide advance notification to the investigator of the impending action prior to it taking effect.

USAMRMC and GSK will promptly inform all other investigators and/or institutions conducting the study if the study is suspended or terminated for safety reasons, and will also inform the regulatory authorities of the suspension or termination of the study and the reason(s) for the action. If required by applicable regulations, the investigator must inform the IEC/IRB promptly and provide the reason for the suspension or termination.

If the study is prematurely discontinued, all study data must be returned to GSK. In addition, arrangements will be made for all unused investigational product(s) in accordance with the applicable GSK procedures for the study.

Financial compensation to investigators and/or institutions will be in accordance with the agreement established between the investigator and USAMRMC and GSK.

12.6. Records Retention

Following closure of the study, the investigator must maintain all site study records in a safe and secure location. The records must be maintained to allow easy and timely retrieval, when needed (e.g., audit or inspection), and, whenever feasible, to allow any subsequent review of data in conjunction with assessment of the facility, supporting systems, and staff. Where permitted by local laws/regulations or institutional policy, some or all of these records can be maintained in a format other than hard copy (e.g., microfiche, scanned, electronic); however, caution needs to be exercised before such action is taken. The investigator must assure that all reproductions are legible and are a true and accurate copy of the original, and meet accessibility and retrieval standards, including re-generating a hard copy, if required. Furthermore, the investigator must ensure there is an acceptable back-up of these reproductions and that an acceptable quality control process exists for making these reproductions.

USAMRMC and GSK will inform the investigator of the time period for retaining these records to comply with all applicable regulatory requirements. The minimum retention time will meet the strictest standard applicable to that site for the study, as dictated by any institutional requirements or local laws or regulations, or USAMRMC/GSK standards/procedures; otherwise, the retention period will default to 15 years.

It is the policy of the U.S. Army Medical and Materiel Command that data sheets are to be completed on all subjects participating in research for entry into the Command's Volunteer Registry Database. This is a confidential database and the data entered includes name, address, social security or equivalent identification number and details of the clinical study. This information is needed to answer questions concerning subjects participating in research sponsored by USAMRMC, and to ensure that subjects can be contacted if there is new information on the study drug. The information should be stored for 75 years. This information will be collected once the subject has agreed to participate in the study and signed the informed consent.

The investigator must notify USAMRMC and GSK of any changes in the archival arrangements, including, but not limited to, the following: archival at an off-site facility, transfer of ownership of the records in the event the investigator leaves the site.

12.7. Provision of Study Results and Information to Investigators

When a clinical study report is completed, USAMRMC and GSK will provide the major findings of the study to the investigator.

In addition, details of the study treatment assignment will be provided to the investigator to enable him/her to review the data to determine the outcome of the study for his/her subject.

12.8. Information Disclosure and Inventions

Ownership:

All information provided by USAMRMC and GSK and all data and information generated by the site as part of the study (other than a subject's medical records) are the sole property of USAMRMC and GSK.

All rights, title, and interests in any inventions, know-how or other intellectual or industrial property rights which are conceived or reduced to practice by site staff during the course of or as a result of the study are the sole property of USAMRMC and GSK, and are hereby assigned to USAMRMC and GSK.

If a written contract for the conduct of the study which includes ownership provisions inconsistent with this statement is executed between USAMRMC and GSK and the study site, that contract's ownership provisions shall apply rather than this statement.

Confidentiality:

All information provided by USAMRMC and GSK and all data and information generated by the site as part of the study (other than a subject's medical records) will be kept confidential by the investigator and other site staff. This information and data will not be used by the investigator or other site personnel for any purpose other than conducting the study. These restrictions do not apply to: (1) information which becomes publicly available through no fault of the investigator or site staff; (2) information which it is necessary to disclose in confidence to an IEC or IRB solely for the evaluation of the study; (3) information which it is necessary to disclose in order to provide appropriate medical care to a study subject; or (4) study results which may be published as described in the next paragraph. If a written contract for the conduct of the study which includes confidentiality provisions inconsistent with this statement is executed, that contract's confidentiality provisions shall apply rather than this statement.

Publication:

Prior to submitting for publication, presentation, use for instructional purposes, or otherwise disclosing the study results generated by the site (collectively, a "Publication"), the investigator shall provide USAMRMC and GSK with a copy of the proposed Publication and allow a period of at least thirty (30) days [or, for abstracts, at least five (5) working days] to review the proposed Publication. Proposed Publications shall not include either USAMRMC or GSK confidential information other than the study results or personal data on any subject, such as name or initials.

At USAMRMC and GSK's request, the submission or other disclosure of a proposed Publication will be delayed a sufficient time to allow USAMRMC and GSK to seek

patent or similar protection of any inventions, know-how or other intellectual or industrial property rights disclosed in the proposed Publication.

If a written contract for the conduct of the study, which includes publication provisions inconsistent with this statement is executed, that contract's publication provisions shall apply rather than this statement.

12.9. Data Management

Subject data are collected by the investigator or designee using the electronic Case Report Form (eCRF) defined by GSK. Subject data necessary for analysis and reporting will be entered/transmitted into a validated database. Clinical data management will be performed in accordance with applicable GSK standards and data cleaning procedures. The site will receive electronic data queries from checks run on the data at site and checks run in-house. The investigator or designee will need to edit the data accordingly or resolve the queries electronically.

The site will keep a copy of the investigator signature sheet and send the original copy to GSK.

Database freeze will occur when data management quality control procedures are completed. Once the study has been completed, the investigator will receive a copy of the data entered at their site on a compact disc (PDF format). It will include all subject data and all data queries and will need to be kept by the investigator according to regulatory requirements.

Data Security

Access to the data will be strictly controlled. The computer equipment will be maintained in good working condition, in a secured area with limited access, and used exclusively for the collection of clinical data for this study. Access to the eCRF will be password-controlled. Unique password(s) will be assigned to the appropriate site personnel at the time of training or installation. All subject data transmitted during the conduct of the study will be identified only by the unique subject number. Additional subject data (name, initials, etc.) collected for use by the clinical site personnel are not transmitted and so are not entered into the GSK database.

12.10. Independent Data Monitoring Committee (IDMC)

An IDMC will be utilized during the conduct of this study. An IDMC is generally assembled when there are significant safety or efficacy issues that warrant external objective medical and/or statistical review in order to protect the ethical and safety interests of subjects and to protect the scientific validity of the study. A copy of the IDMC charter is available from GSK upon request. It details the full responsibilities of the IDMC and a copy will be made available to the investigator.

The IDMC will consist of experts in nephrology, ophthalmology and malaria and a statistician from an independent SDAC, and will assist in the following:

1. Review of all relevant efficacy and safety data from a planned interim analysis after all subjects in Cohort 1 have completed the Day 28 assessment. The IDMC will determine if pre-defined efficacy and safety criteria, as described in Section 11.3.1 have been met and advise on the appropriateness of initiating enrollment for Cohort 2.

2. Review of ongoing efficacy and safety data from this study if any of the following criteria are fulfilled:

   - If ≥4 Early Treatment Failures (ETFs) occur among the first 21 subjects enrolled into the Cohort and an independent Statistical Data Analysis Center (SDAC) confirms that the actual response rate among evaluable tafenoquine subjects is below 70%
   - A sustained (≥ 14 days) serum creatinine increase of ≥26.6 µmol/L (0.3 mg/dL) is confirmed in ≥8 subjects
   - If at any timepoint, retinal changes from baseline that cause clinical concern (these would include decreased vision, bull’s eye retinopathy, distortions observed on the Amsler Grid test, abnormal color vision or development of a scotoma on visual field testing) are seen in ≥2 subjects
   - If ≥5 subjects have the following same drug related AE (based on body systems) of severe intensity:
     - Methemoglobin concentration of ≥ 20%
     - Repeated (> 4 times within one day) or intractable vomiting
     - Severe diarrhea resulting in loss of intravascular volume judged by the investigator to be sufficiently severe to warrant the initiation or increase in rate of administration of intravenous replacement fluids after onset of diarrhea
     - Nausea resulting in no significant oral intake, requiring intravenous fluids

Enrollment of further subjects will be put on hold and an *ad hoc* meeting of the IDMC will be called. The IDMC will review all the available relevant safety and efficacy study data and advise on the appropriateness of continuing the study. Outputs for an IDMC will be produced by an independent SDAC appointed by GSK. All GSK study related personnel will remain blinded to the treatment codes for both cohorts until the end of the study.

13. REFERENCES

Clopper CJ and Pearson ES. The use of confidence or fiducial limits illustrated in the case of the binomial. *Biometrika* 1934; 26: 404-413,

Landewe RB. et al. Antimalarial drug induced decrease in creatinine clearance. *J. of Rheumatology* 1995; 22:34-7.

Obaldia N 3rd, Rossan RN, Cooper RD, Kyle DE, Nuzum EO, Rieckmann KH, Shanks GD. WR 238605, chloroquine, and their combinations as blood schizonticides against a chloroquine-resistant strain of *Plasmodium vivax* in Aotus monkeys. *Am J Trop Med Hyg*. 1997; 56:508-10

Peters W, Robinson BL and Milhous WK. The chemotherapy of rodent malaria. LI. Studies on a new 8-aminoquinoline, WR 238605. *Ann Trop Med Parasitol*. 1993; 87:547-552.

Puri SK and Dutta GP. Blood schizontocidal activity of WR 238605 (Tafenoquine) against *Plasmodium cynomolgi* and *Plasmodium fragile* infections in rhesus monkeys. *Acta Tropic* 2003; in press.

Sachs J and Malaney P. The economic and social burden of malaria. *Nature* 2002; 415:680-5.

14. APPENDICES

14.1. Appendix 1: Time and Events Table

Cohort 1

PROCEDURE	DAYS																										
	0	1	2	3	4	5	6	7	8	9	10	11	12	13	14	15	16	17	18	19	20	21	28	29	30	60	90	120
Informed Consent	X																											
G6PD (1 sample)	X																											
Eligibility Criteria	X																											
Parasitological Assessment (smear)	X[1]	X[1]	X[1]	X[1]	X[1]	X[1]	X[1]	X[1]							X								X			X	X	X
Demography	X																											
Pregnancy Test (urine or serum)	X							X														X					X	
Physical Examination	X																											
Vital Signs	X[2]	X[2]	X[2]	X[2]	X[2]	X[2]	X[2]	X[2]							X								X			X	X	
History of Malaria, Medical History	X																											
Eye Examinations	X[3]																						X				X	
Baseline Signs and Symptoms	X																											
Prior/Concomitant Medication	X	X	X	X	X	X	X	X	X	X	X	X	X	X	X	X	X	X	X	X	X	X	X	X	X	X	X	
Randomization	X																											
**Study Medication Tafenoquine Arm**																												
Tafenoquine + Chloroquine Placebo	X	X	X																									
Primaquine Placebo				X	X	X	X	X	X	X	X	X	X	X	X	X	X											
**Study Medication Chloroquine/Primaquine Arm**																												
Chloroquine + Tafenoquine Placebo	X	X	X																									
Primaquine				X	X	X	X	X	X	X	X	X	X	X	X	X	X											

Continued

Cohort 1 (Continued)

**PROCEDURE**	**DAYS**																											
	**0**	**1**	**2**	**3**	**4**	**5**	**6**	**7**	**8**	**9**	**10**	**11**	**12**	**13**	**14**	**15**	**16**	**17**	**18**	**19**	**20**	**21**	**28**	**29**	**30**	**60**	**90**	**120**
Adverse Experiences	X	X	X	X	X	X	X	X	X	X	X	X	X	X	X	X	X	X	X	X	X	X	X	X	X	X	X	X[4]
Safety Samples – hematology	X			X				X							X													
Safety Samples – clinical chemistry	X			X				X							X							X	X				X	
Urinalysis	X							X															X					
Methemoglobin Assessment	X			X				X														X						
Population Pharmacokinetic Samples[5]	X	X	X	X------------------X									X--X										X------------X					

1. Blood smears to be conducted every 12 hours (± 2 hours) until negative for 2 consecutive smears. Once confirmed will be obtained once a day up to and including Day 7.
2. Body temperature to be measured every 12 hours (± 2 hours)
3. Baseline eye examinations (visual acuity, amsler grid, color vision, corneal and retinal examination/photos) will performed prior to first dose of study drug if possible, but no later than within 36 hours of receiving the first dose of study drug.
4. Serious Adverse events only
5. Samples will be taken within specified time windows. See Study Assessments and Procedures section for details

Cohort 2

PROCEDURE	DAYS																										
	0	1	2	3	4	5	6	7	8	9	10	11	12	13	14	15	16	17	18	19	20	21	28	29	30	60	90	120
Informed Consent	X																											
G6PD (1 sample)	X																											
Eligibility Criteria	X																											
Parasitological Assessment (smear)	X[1]	X[1]	X[1]	X[1]	X[1]	X[1]	X[1]	X[1]							X								X			X	X	X
Demography	X																											
Pregnancy Test (urine or serum)	X							X														X					X	
Physical Examination	X																											
Vital Signs	X[2]	X[2]	X[2]	X[2]	X[2]	X[2]	X[2]	X[2]							X								X			X	X	
History of Malaria, Medical History	X																											
Eye Examinations	X[3]																						X				X	
Baseline Signs and Symptoms	X																											
Prior/Concomitant Medication	X	X	X	X	X	X	X	X	X	X	X	X	X	X	X	X	X	X	X	X	X	X	X	X	X	X	X	
Randomization	X																											
**Study Medication Tafenoquine Arm**																												
Tafenoquine	X																											
Chloroquine Placebo	X	X	X																									
Primaquine Placebo				X	X	X	X	X	X	X	X	X	X	X	X	X	X											
**Study Medication Chloroquine/Primaquine Arm**																												
Chloroquine	X	X	X																									
Tafenoquine Placebo	X																											
Primaquine				X	X	X	X	X	X	X	X	X	X	X	X	X	X											

Continued

Cohort 2 (Continued)

PROCEDURE	DAYS																										
	**0**	**1**	**2**	**3**	**4**	**5**	**6**	**7**	**8**	**9**	**10**	**11**	**12**	**13**	**14**	**15**	**16**	**17**	**18**	**19**	**20**	**21**	**28**	**29**	**30**	**60**	**90**	**120**
Adverse Experiences	X	X	X	X	X	X	X	X	X	X	X	X	X	X	X	X	X	X	X	X	X	X	X	X	X	X	X	X[4]
Safety Samples – hematology	X			X				X							X													
Safety Samples – clinical chemistry	X			X				X							X							X	X				X	
Urinalysis	X							X															X					
Methemoglobin Assessment	X			X				X														X						
Population Pharmacokinetic Samples[5]	X	X	X	X-------------------X									X--X										X-----------X					

1. Blood smears to be conducted every 12 hours (± 2 hours) until negative for 2 consecutive smears. Once confirmed will be obtained once a day up to and including Day 7.
2. Body temperature to be measured every 12 hours (± 2 hours)
3. Baseline eye examinations (visual acuity, amsler grid, color vision, corneal and retinal examination/photos) will performed prior to first dose of study drug if possible, but no later than within 36 hours of receiving the first dose of study drug.
4. Serious Adverse events only.
5. Samples will be taken within specified time windows. See Study Assessments and Procedures section for details.

14.2. Appendix 2: Ophthalmic Assessment Details

Macular Function Tests:

Test: Amsler Grid

Purpose: Determine macular function and detect defects such as metamorphopsia (distortions of vision, usually due to localized degenerations of retinal tissue or fluid within the retinal layers) or relative or absolute scotomas (due to loss of function of the retinal receptors, generally cones in the macular area).

Measurement: Number, size and shape of macular defects.

How measured: The Amsler grid test is a subjective test in which the subject views a black grid on a white background at a distance of 12 inches (30 cm) with one eye at a time (monocular measurement). The subject is asked if all four corners and sides of the square are present while they concentrate on the center dot. Then they are asked if there are any wavy lines in the grid, blurred areas or missing areas. The subject will be asked to sketch out the areas of metamorphopsia or scotoma.

How analyzed: Distortions or missing areas are noted.

Norms: No missing corners or sides to the square, no distortions of the lines, no blurred areas and no missing areas.

Significance: The development of any anomaly on the Amsler Grid is considered significant (the examiner must be careful to make sure the subject's tear layer is not drying up during this test as this can lead to false positives; additionally the subject must be properly corrected for the testing distance, especially presbyopes, and the test should not be completed right after exposure to bright light, such as a camera flash or ophthalmoscopy).

Test: Humphrey 10-2 Visual Field

Purpose: Determination of threshold sensitivity of specific loci in the central retina and detection and definition of relative or absolute scotomas.

Measurement: Threshold sensitivity of 68 points in the central 10 degree radius area of the retina.

How measured: The subject fixates on a central point in a visual field testing bowl and responds to targets of decreasing brightness presented in the paracentral region. Using a SITA testing paradigm (a stairstep thresholding procedure), the threshold of 68 points can be determined in 3 to 4 minutes per eye.

How analyzed: The Humphrey system includes a STATPAC analysis program. This program provides statistics on changes in the visual field over time, including mean

deviation from normal, depth of field defects and corrected pattern standard deviation. Each of these parameters is analyzed in terms of probability.

Norms: Mean threshold is age dependent. For the expected test population, the mean threshold is estimated to be 28 decibels (+/- 5 dB, 1 Standard Deviation (SD)).

Significance: A decrease of sensitivity (increase in threshold) of 5 decibels either at local areas or overall is significant.

Test: Macular stress test

Purpose: Determination of macular function, specifically the photoreceptor outer segments and retinal pigment epithelial function. Sometimes dubbed the “poor man’s electroretinography.”

Measurement: Number of seconds required to recover vision after exposure to a bright light source.

How measured: Determine and record the subject’s best-corrected visual acuity (BCVA). Occlude one eye and place a transilluminator (or strong penlight) 3 to 5 cm in front of the other eye. The subject stares at this light for 10 seconds. The light is removed and the subject is asked to read the eyechart again.

How analyzed: The number of seconds required to read one line above BCVA is the endpoint.

Norms: Normal recovery time is 50 to 60 seconds (mean 50 seconds +/- 15 seconds, 1 SD); greater than 90 seconds is considered abnormal recovery.

Significance: An increase of 30 seconds in recovery time from baseline measurement will be considered significant.

Test: High contrast visual acuity (HCVA) – ETDRS chart

Purpose: Determine retinal resolution to high contrast, high spatial frequency targets (letters).

Measurement: Minimum angle of resolution (MAR) in minutes of arc describing the smallest detail of the target, converted to the logarithm of the MAR (logMAR).

How measured: The subject views a backlit chart with letters of decreasing size (resolution angle) and attempts to read the smallest letters they are able to see. To determine threshold, the examiner has the subject continue to read smaller letters on the chart until 3 or more letters are missed on a line of 5 letters. The subject must be wearing their manifest refraction to determine best spectacle-corrected visual acuity.

How analyzed: Threshold is calculated based on the number of letters correctly identified, with each letter on the chart carrying a weight of 0.2 logMAR.

Norms: For the expected population, the norm is –0.13 logMAR (+/- 0.09, 1 SD). This converts to a 20/15 Snellen equivalent.

Significance: A change of 4 letters or more (0.08 logMAR) is considered significant.

Test: Color vision – PIP plates

Purpose: Assessment of macular function, specifically the cone photoreceptors and the neural path supporting the cones.

Measurement: Number of plates correctly identified out of 14 presentations (depends on version of PIP charts)

How measured: The plates must be properly lit with a C luminance bulb (Macbeth easel lamp works best for this). The plates are held 30 inches from the subject and presented one at a time for 2 seconds each. The subject must properly identify the number embedded in the plate.

How analyzed: Misidentification of certain plates is due to either congenital or acquired color deficiency. The PIP test only determines red-green (deutan or protan) deficiency. Blue-yellow (tritan) deficiency is more common in acquired conditions and requires use of other tests, such as the D-15, 40 hue or 100 hue tests.

Norms: 8% of all males have some form of color deficiency, most anomalous trichromats (either deutan or protan); 0.64% of females are color deficient. Acquired color deficiency due to retinal or neural degenerations is generally tritan in nature. Individuals with normal color vision will miss no more than 4 plates in the presentation set.

Significance: Development of a color deficiency is an indication of a change in retinal function. A change in score of 2 or more plates is significant. The test is generally considered a screening test, not a diagnostic test. Failure would require further testing on a more specific color test, such as the Lanthony 40-hue.

Test: Color vision – Lanthony 40 hue

Purpose: Assessment of macular function, specifically the cone photoreceptors and the neural path supporting the cones. This is a more specific test than the PIP plates in that the axis of color confusion can be determined.

Measurement: Errors in arrangement of colored caps.

How measured: The subject arranges four sequences of colored caps each with a different level of saturation. Caps that appear gray in color to the subject are removed and only the remaining caps are arranged. These caps constitute the saturation threshold at particular colors for that subject. The results are plotted on a chart indicating the specific color confusions.

How analyzed: Confusion of certain colors is due to either congenital or acquired color deficiency and may be a red-green (deutan or protan) or blue-yellow (tritan) deficiency.

Norms: 8% of all males have some form of color deficiency; 0.64% of females are color deficient. Acquired color deficiency due to retinal or neural degenerations is generally tritan in nature. Individuals with normal color vision may make mistakes within the color circle, but rarely miss colors across the color circle. In other words, reversals of color caps in the 40-hue test are considered normal if the errors are between adjacent colors.

Significance: Development of a color deficiency is an indication of a change in retinal function. The development of 2 or more confusions across the color axis is significant.

Digital Photography:

Test: Corneal digital photographs

Purpose: Document presence of corneal deposits.

Measurement: Deposition density per millimeter squared over the central 3-millimeter zone.

How measured: Digital photographs will provide information on backscattered light from the corneal surface. Light scattered by particle depositions in the anterior cornea (subepithelial region) will appear as lighter areas in the digital images.

How analyzed: A central 3 mm zone is selected and luminance level determined using either the built-in programs on the Topcon analysis system or after importing the images into Adobe Photoshop. In Adobe Photoshop, luminance in the form of a histogram can be extracted for analysis.

Norms: Normal corneas reflect 3 to 4% of incident light.

Significance: In the previous assessment of corneal deposition, the criteria used were presence or absence of deposits (binomial). In order for an observer to detect corneal deposits, the contrast between the particles and the background image of the cornea has to be sufficient. The presence or absence of deposits will be verified through review of the photographs by trained vision experts (optometrists or ophthalmologists). To get a quantitative value for the backscatter, backscattered light in pre-drug corneas will be used as baseline (this will provide a luminance value unique to that cornea); a significant change in the post-drug cornea would be an increase in reflected light by 10% or greater.

Test: Retinal digital photographs

Purpose: Document changes in retinal morphology

Measurement: Retinal uniformity over the central 10 degrees around the macula and presence of pigment clumping or depigmentation out to 30 degrees from the macula.

How measured: Digital images will be taken of the central retina.

How analyzed: Using digital overlay techniques, difference maps will be created to show areas of pigmentation changes of the retina (either increased or decreased pigmentation).

Norms: Individual retinal pigmentation varies; therefore the baseline retinal image is the best “norm” for this test.

Significance: The qualitative level of retinal uniformity will be verified through review of the photographs by trained vision experts (optometrists or ophthalmologists). Using the digital comparison technique, a change in retinal uniformity, due to the development of mottling, hyperpigmentation or depigmentation, by more than 10% will be considered significant.

14.3. Appendix 3: Organization Of Research Effort

<u>Principal Investigator:</u>

[redacted] MD.
[redacted] Thailand
Tel. [redacted] Fax [redacted]
Email: [redacted]

<u>Co-Investigators</u>

[redacted] M.D.
[redacted] Thailand
Tel. [redacted] Fax [redacted]
Email: [redacted]

[redacted] MD.
[redacted] Thailand
Tel. [redacted] Fax [redacted]
Email: [redacted]

<u>Research Staff</u>

[redacted] MD. (Study ophthalmologist)
[redacted] Thailand
Tel. [redacted] Fax [redacted]
Email: [redacted]

[redacted] M.D., Ph.D. (Study physician)
[redacted] Thailand
Tel. [redacted] Fax [redacted]

[redacted] M.D. (Study physician)
[redacted]
[redacted] Thailand
Tel. [redacted] Fax [redacted]
Email: [redacted]

[redacted] MD.(Study physician)
[redacted]
[redacted] Thailand
Tel. [redacted] Fax [redacted]
Email: [redacted]

[redacted] (Study ophthalmologist)
[redacted]
[redacted] Thailand
Tel. [redacted] Fax [redacted]

[redacted] MD. (Study physician)
[redacted]
[redacted] Thailand
Tel. [redacted] Fax [redacted]

[redacted] MD. (Study physician)
[redacted]
[redacted] Thailand
Tel. [redacted] Fax [redacted]
Email: [redacted]

[redacted] RN (Study coordinator)
[redacted]
[redacted] Thailand
Tel. [redacted]

[redacted] RN (Study Nurse)
[redacted] Thailand
Tel. [redacted]

[redacted] MD (Medical Monitor)
[redacted] Thailand
Tel. [redacted] Fax [redacted]

[redacted] MD (Sponsor's Medical Expert)
Division of Experimental Therapeutics
Room 1A28
Walter Reed Army Institute of Research
503 Robert Grant Avenue
Silver Spring, MD 20910-7500
[redacted] Fax [redacted]

14.4. Appendix 4: Research Facilities and IEC/IRBs

Research Facilities

[redacted] Thailand
TEL: [redacted]

[redacted] Thailand.
Tel : [redacted]

[redacted] Thailand
Tel: [redacted]

IEC/IRBs

[redacted] Thailand
Tel: [redacted]

[redacted] Thailand
Tel: [redacted]

[redacted] USA
Tel: [redacted]

14.5. Appendix 5: Protocol Changes

Protocol Amendment 01 Changes

The following changes were made to the original protocol, approved 12 March 2003, for study SB252263/058: A randomized, active-control, double-blind, double-dummy study to evaluate the efficacy and safety of tafenoquine for the treatment of *Plasmodium vivax* in adults.

1. Title page. Sponsor Signatory changed to GSK Signatory as Office of the Surgeon General, Department of the Army is the sponsor for tafenoquine and GSK is its co-development partner.
2. Information page has been modified to clarify that Office of the Surgeon General, Department of the Army is the sponsor for tafenoquine and GSK is its co-development partner.

Was:

SPONSOR INFORMATION PAGE

Title:	A randomized, active-control, double-blind, double-dummy study to evaluate the efficacy and safety of tafenoquine for the treatment of *Plasmodium vivax* in adults
Study Identifier:	058

GlaxoSmithKline
1250 South Collegeville Road
Collegeville, PA 19426, USA
Telephone Number: [redacted]

Sponsor Contact Information:

Dr. [redacted]
GlaxoSmithKline
Mail Code UP4215
1250 S. Collegeville Road
Collegeville, PA 19426

Phone: [redacted]
Fax: [redacted]

IND Number: 38,503

Is:

SPONSOR INFORMATION PAGE

Title:	A randomized, active-control, double-blind, double-dummy study to evaluate the efficacy and safety of tafenoquine for the treatment of *Plasmodium vivax* in adults
Study Identifier:	058

The Sponsor for tafenoquine, IND #38,503, is the Office of the Surgeon General, Department of the Army. Tafenoquine is being developed by the U.S. Army Medical Research and Materiel Command (USAMRMC) in collaboration with its co-development partner GlaxoSmithKline.

Sponsor:
Department of Defense
Office of the Surgeon General, Department of the Army

Point Of Contact, Coordinating Office:
U.S. Army Medical Research and Material Command
Office of Regulatory Compliance and Quality
504 Scott Street
Fort Detrick, MD 21702-5012
[redacted]

Sponsor Representative :
[redacted] Ph.D.
U.S. Army Medical Materiel Development Activity
622 Neiman Street
Fort Detrick, MD 21702-5012
[redacted]

GSK Representative: GlaxoSmithKline
1250 South Collegeville Road
Collegeville, PA 19426, USA
Telephone Number: [redacted]

Contact Information:
Dr. [redacted]
GlaxoSmithKline
Mail Code UP4215
1250 S. Collegeville Road
Collegeville, PA 19426
Phone: [redacted] Fax: [redacted]

IND Number: 38,503

3. Abbreviations. Case Record Form was corrected to Case Report Form. Abbreviations and definitions for [redacted] DMID, ITT, PP, RAMOS, and USAMRMC were added.

CRF	Case Report Form
[redacted]	[redacted]
DMID	Division of Microbiology and Infectious Diseases
ITT	Intent To Treat
PP	Per Protocol
RAMOS	Registration and Medication Ordering System
USAMRMC	United States Army Medical Research and Materiel Command

4. Protocol Summary, sub-section Rationale, 4th paragraph, 1st sentence. Spelling of gametocytocidal corrected.
5. Protocol Summary, sub-section Rationale, 5th paragraph, 1st sentence. Text clarifying that this study is a phase II study added. Change is underlined:

This Phase II study is designed to determine whether a single 600 mg dose or 400mg/day for 3 days of tafenoquine is efficacious, and well tolerated for clearing *P.vivax* malaria infection (blood schizontocidal and gametocytocidal activity) and preventing *P. vivax* relapse (hypnozoite eradication).

6. Protocol Summary, sub-section Endpoints, paragraph following Late Treatment Failure. Text added at end of paragraph, clarifying Safety and Intent to Treat populations.

(All randomized subjects who received at least one dose of study medication (i.e. tafenoquine or chloroquine/primaquine) will be included in the Safety and Intent to Treat (ITT) analysis populations.)

7. Protocol Summary, sub-section Study Design, 1st paragraph, 2nd sentence. Text corrected from 28 days to 29 days to indicate subjects will be hospitalized for the first 29 days of the study and to clarify that they will be asked to remain in a malaria free region as the term ‘local’ is not well-defined.

Subjects enrolled into the study will be hospitalized at the [redacted] for the first 29 days of the study and will be asked to remain ~~local~~ in a malaria free region until 90 days after the start of the study for follow up with scheduled assessments at Days 60 and 90.

8. Protocol Summary, sub-section Study Population section, 1st paragraph, 2nd sentence. Text modified to clarify the recruitment process. Text corrected from 28 days to 29 days to indicate subjects will be hospitalized for the first 29 days of the study and to clarify that they will be asked to remain in a malaria free region as the term ‘local’ is not well-defined.

Was: Subjects will be recruited from the Thailand-Myanmar border and brought to the [redacted] where they will be hospitalized for the first 29 days of the study for close monitoring and asked to remain local until 90 days after the start of the study, to reduce the chance of re-infection.

Is: Subjects presenting to [redacted] health care system with microscopy proven *Plasmodium vivax* malaria will be offered enrollment. They will be hospitalized for the first 29 days of the study for close monitoring and asked to remain where malaria is not endemic until 90 days after the start of the study, to reduce the chance of re-infection.

9. Protocol Summary, sub-section Study Assessments and Procedures section, 1st paragraph, 5th sentence. Text added to correct omission of methemoglobin as an assessment parameter.

A blood sample will be taken for biochemistry, methemoglobin, and hematology analysis and a urine sample provided for urinalysis.

10. Protocol Summary, sub-section Study Assessments and Procedures section, 2nd paragraph, 2nd sentence. Sentence has been modified to permit delaying the full eye exam if subject is deemed too ill to undergo the required testing. The window for completing the full eye exam after dosing has been increased from 24 hours to 36 hours to permit sufficient time for improvement of symptoms for these subjects, before undergoing the full eye examination.

Was: If the scheduling of the full eye exam will significantly delay the start of treatment (> 10 hours), a basic eye exam to assess vision problems will be performed prior to dosing, with a full eye exam to be conducted as soon as possible but no later than within 24 hours of receiving the first dose of study drug.

Is: If the scheduling of the full eye exam will significantly delay the start of treatment (> 10 hours) or if the subject is deemed by the investigator to be too ill to undergo the complete eye examination, a basic eye exam to assess vision problems will be performed prior to dosing. The full eye exam will then be conducted as soon as possible but no later than within 36 hours of receiving the first dose of study drug.

11. Protocol Summary, sub-section Clinical Chemistry and Hematology. Sub-section title changed to correct omission of methemoglobin.

Clinical Chemistry, Methemoglobin, and Hematology

12. Protocol Summary, sub-section Clinical Chemistry and Hematology, 1st paragraph, 2nd sentence. Text added to correct omission of GGT as an assessment parameter.

For biochemistry the following parameters will be assessed: blood urea nitrogen (BUN), creatinine, glucose, sodium, potassium, chloride, total carbon dioxide, calcium, aspartate aminotransferase (AST), alanine aminotransferase (ALT), gamma-glutamyl transferase (GGT), alkaline phosphatase, total bilirubin, albumin, and total protein.

13. Protocol Summary, sub-section Clinical Chemistry and Hematology, 1st paragraph. Text added at end of paragraph to correct omission of methemoglobin as an assessment parameter.

A blood sample will be taken to determine methemoglobin concentration at baseline and Days 3, 7, and 21.

14. Protocol Summary, sub-section Efficacy Assessments, 1st paragraph, 1st sentence. Text added to allow for ±2 hour window around blood smears.

Thick and thin blood smears for malaria will be obtained by finger prick at baseline (Day 0) and then every 12 hours (± 2 hours) up to and including Day 7, until the blood smear becomes negative.

15. Protocol Summary, sub-section Efficacy Assessments, 1st paragraph, 6th sentence. Text added to define abbreviation for IDMC.

If based on these data, the SDAC confirms that the actual response rate among evaluable tafenoquine subjects is below 70%, enrollment will remain suspended and the SDAC will be required to call an ad-hoc Independent Data Monitoring Committee (IDMC) meeting.

16. Protocol Summary, sub-section Population Pharmacokinetics, after bullet number vi. Additional bullet, vii, added to indicate that a PK sample will be taken for recurrences of *P. vivax*, after Day 7 up to and including Day 28.

vii At recurrence of *P. vivax* after Day 7 up to and including Day 28 if a PK sample has not been drawn within the previous 24 hours

17. Protocol Summary, sub-section Population Pharmacokinetics, end of last paragraph. Text added to indicate working instructions will be provided to insure sample times are adequately bracketed.

Working instructions will be developed and followed to insure that sampling times will be adequately bracketed within the sampling window.

18. Section 1.1 Background, 8th paragraph, 1st sentence. Spelling of gametocytocidal corrected.

19. Section 1.1 Background, end of section. Sub-section entitled Reproductive Toxicity added to briefly summarize data for tafenoquine since women of child-bearing potential are not excluded from this study.

Reproductive Toxicity

No adverse effects on fertility or embryofetal development (including at maternally toxic doses), or on postnatal survival, were observed in a complete battery of reproductive toxicology studies conducted in male and female rats and female rabbits. Full details of these studies are given in the Clinical Investigators Brochure (CIB).

20. Section 1.2 Rationale, 4th paragraph, 1st sentence. Spelling of gametocytocidal corrected.

21. Section 1.2 Rationale, 5th paragraph, 1st sentence. Text clarifying that this study is a phase II study added.

This Phase II study is designed to determine whether a single 600 mg dose or 400mg/day for 3 days of tafenoquine is efficacious, and well tolerated for clearing *P.vivax* malaria infection (blood schizontocidal and gametocytocidal activity) and preventing *P. vivax* relapse (hypnozoite eradication).

22. Section 3.1 Primary, last paragraph. Text added at end of paragraph clarifying Safety and Intent to Treat populations.

(All randomized subjects who received at least one dose of study medication (i.e. tafenoquine or chloroquine/primaquine) will be included in the Safety and Intent to Treat (ITT) analysis populations.)

23. Section 4 Study Design. Section 4 was sub-divided into additional sub-sections, entitled, 4.1 General Study Design, 4.2 Risks and Benefits to Research Subjects, 4.2.1 Risks to Research Subjects, and 4.2.2 Benefits to Research Subjects to allow more complete descriptions of the study logistics and study specific risks and benefits to subjects.

24. Section 4 Study Design. Text from this section was incorporated into the new sub-section 4.1 General Study Design includes with additional text added to better describe study logistics (changes indicated by underline).

Was: This will be a randomized, active-control, double-blind, double-dummy study conducted in 2 sequential cohorts in a total of 140 subjects enrolled in order to obtain 120 evaluable (60/cohort) male and female subjects age 20-60 years with confirmed *P. vivax* malaria. Subjects enrolled into the study will be hospitalized at the [redacted] for the first 28 days of the study and will be asked to remain local until 90 days after the start of the study for follow up with scheduled assessments at Days 60 and 90. Subjects will be contacted at Day 120 for a follow-up blood smear. Subjects will be given tafenoquine alone or the standard regimen of chloroquine followed by primaquine (2:1 ratio, 40:20 subjects in each cohort). Subjects will provide written informed consent and will undergo screening and baseline procedures before the start of the study. Subjects in Cohort 1 will be randomized to receive either tafenoquine (400mg base) and chloroquine placebo for 3 days, followed by primaquine placebo for 14 days, or chloroquine (1000 mg chloroquine phosphate) and tafenoquine placebo for 2 days, followed by chloroquine (500 mg chloroquine phosphate) and tafenoquine placebo for 1 day, followed by primaquine, 15 mg/day for 14 days.

The efficacy (Day 28 cure rate) of the dosing regimen in Cohort 1 will be compared to prespecified criteria (as detailed in Section 11.3) once all subjects have completed the 90 day assessment. These efficacy results and the safety profile will be examined before enrolling Cohort 2. Subjects in Cohort 2 will be randomized to receive either tafenoquine (600 mg base) and chloroquine placebo for 1 day, followed chloroquine placebo for 2 days, followed by primaquine placebo for 14 days, or chloroquine (1000 mg chloroquine phosphate) and tafenoquine placebo for 1 day, followed by chloroquine (1000 mg chloroquine phosphate) for 1 day, followed by chloroquine (500 mg chloroquine phosphate) for 1 day, followed by primaquine, 15 mg/day for 14 days.

Subjects will remain in the study for 121 days from start of treatment to end of routine follow-up. Further follow-up will occur for any renal or ophthalmic findings at the discretion of the investigator.

Is: This will be a randomized, active-control, double-blind, double-dummy study conducted in 2 sequential cohorts in a total of 140 subjects enrolled in order to obtain 120 evaluable (60/cohort) male and female subjects age 20-60 years with confirmed *P. vivax* malaria.

The study will be conducted at the [redacted] located in [redacted] Thailand (hereafter referred to as the [redacted] This institution enjoys a worldwide reputation for research on the treatment of malaria and malaria-related complications. The Hospital has a staff of approximately 50 physicians, several hundred nurses, and 15-20 laboratory technicians. In addition to a large outpatient clinic, [redacted] has 250 beds, a 6 bed ICU capable of supporting hemodialysis (for malaria-related renal complications), mechanical ventilation (for the respiratory support of cerebral malaria), and laboratory facilities capable of supporting all aspects of malaria diagnostics and treatment, from expert microscopy to blood banking facilities capable of supporting exchange transfusions for severe malaria.

Study volunteers will be recruited by a study investigator or nurse, from persons presenting either at the [redacted] outpatient clinic or admitted to the inpatient ward following transfer from the [redacted] [redacted] clinic, for evaluation of fever. Subjects deemed eligible for the study will be consented and enrolled if eligible and willing to participate. Subjects found not eligible or unwilling to participate in the study will be treated as clinically warranted. Subjects originally transported from the [redacted] clinic, who are found not eligible or unwilling to participate in the study, will receive free treatment as clinically warranted and provided free transportation back to [redacted] when clinically warranted. Subjects enrolled into the study will be hospitalized at the [redacted] for the first 29 days of the study and will be asked to remain in a malaria non-endemic area until 90 days after the start of the study for follow up with scheduled assessments at Days 60 and 90. While hospitalized subjects enrolled in this study will have the same [redacted] visitation policy as are those subjects who are not research participants.

Subjects will be contacted at Day 120 for a follow-up blood smear. Subjects will be given tafenoquine alone or the standard regimen of chloroquine followed by primaquine (2:1 ratio, 40:20 subjects in each cohort). Subjects will provide written informed consent and will undergo screening and baseline procedures before the start of the study. Subjects in Cohort 1 will be randomized to receive either tafenoquine (400mg base) and chloroquine placebo for 3 days, followed by primaquine placebo for 14 days, or chloroquine (1000 mg chloroquine phosphate) and tafenoquine placebo for 2 days, followed by chloroquine (500 mg chloroquine phosphate) and tafenoquine placebo for 1 day, followed by primaquine, 15 mg/day for 14 days.

The efficacy (Day 28 cure rate) of the dosing regimen in Cohort 1 will be compared to pre-specified criteria (as detailed in Section 11.3) once all subjects have completed the 90 day assessment. These efficacy results and the safety profile will be examined before enrolling Cohort 2. Subjects in Cohort 2 will be randomized to receive either tafenoquine (600 mg base) and chloroquine placebo for 1 day, followed by chloroquine placebo for 2 days, followed by primaquine placebo for 14 days, or chloroquine (1000 mg chloroquine phosphate) and tafenoquine placebo for 1 day, followed by chloroquine (1000 mg chloroquine phosphate) for 1 day, followed by chloroquine (500 mg chloroquine phosphate) for 1 day, followed by primaquine, 15 mg/day for 14 days.

Subjects will remain in the study for 121 days from start of treatment to end of routine follow-up. Further follow-up will occur for any renal or ophthalmic findings at the discretion of the investigator.

25. Section 4.2.1 Risks to Research Subjects. The following text was added into this new section to better describe the study specific risks of this study.

The subject may experience a brief moment of physical discomfort during the finger-prick procedure and the venipuncture and there is a possibility of bruising and/or infection at the site of the finger-prick or venipuncture. A maximum of 160 ml of blood will be taken from each subject during the study.

Subjects may experience side effects from the drugs. Side affects associated with chloroquine may include nausea, vomiting, blurred vision and headache. More frequent side effects associated with primaquine include nausea, vomiting and abdominal pain. The primary clinical events associated with tafenoquine include gastrointestinal disturbances (vomiting, nausea, diarrhea and abdominal pain) and headaches.

Subjects may experience slight stinging in the eyes after dilation of the pupils with tropicamide 1.0% and phenylephrine 2.5% for the corneal and fundus examinations.

There is increased risk of intravascular hemolysis associated with these drugs in subjects who are G6PD deficient. All subjects considered for this study will be screened for G6PD deficiency. Although it is not anticipated that any subject will be mistakenly enrolled in this study, if this should occur in exceptional circumstances, hemolysis would occur within the initial days following the first dose of trial medication, so the subjects will already be in a hospital setting, therefore prompt medical attention will be readily available.

In a sub-group of study SB252263/033, tafenoquine was shown to cause pigmented deposits on the cornea (vortex keratopathy), after 6 months of weekly dosing in adults. The time to onset of corneal deposit formation could not be established, as assessments were performed at baseline and after 6 months only. These deposits did not result in impairment or loss of vision and were shown to have complete resolution approximately 1 year after stopping study drug. It is not anticipated that these deposits will occur following 3 days of dosing with tafenoquine, however, this will be monitored through the eye assessments at baseline, Day 28 and Day 90.

In this same study some minor findings were seen at the end of the study in the retina. No subject had problems with vision and no major retinal changes were seen during follow-up. The relevance of these minor retinal findings could not be ascertained as baseline retinal photography data had not been obtained, however, this will be monitored through the eye assessments at baseline, Day 28 and Day 90.

In earlier studies, trends towards increased creatinine were seen in subjects receiving tafenoquine. Few subjects had values outside the normal ranges and none were considered clinically significant. These data were reviewed by a panel of clinical nephrologists which concluded that the existing data cannot exclude the occurrence of renal damage, although this is considered unlikely. Kidney function will be monitored through clinical chemistry assessments at baseline and at Days 3, 7, 14, 21, 28 and 90, and through urinalysis at baseline and Days 7 and 28.

All subjects will be closely monitored (daily for 29 days and monthly for a further 3 months) and quickly treated should any signs or symptoms of malaria be detected.

Should a subject be injured as a direct result of participating in this study, medical care will be provided, at no cost, for that injury. Injured subjects will also receive a travel and a per diem allowance to cover the cost of food and traveling to medical appointments required to treat a study related injury. Subjects will also be compensated for lost income, 170 Baht/day, resulting from treatment for a study related injury.

26. Section 4.2.2 Benefits to Research Subjects added. The following text was added into this new section to better describe the study specific benefits of this study.

As subjects will be hospitalized during the anti-malarial compound administration periods, all treatment doses of chloroquine, tafenoquine, and primaquine will be administered under supervision by medical/nursing staff trained in drug administration, and any change in the course of their infection or any adverse experiences will be recognized and treated more rapidly than would normally occur in the their rural home setting.

Subjects will be immediately treated for any reappearance of parasitemia that occurs during the 3 month follow-up period. Subjects who fail initial therapy, based on parasitological parameters, will be treated with an alternative regimen that is known to be effective. In addition, the subjects will be examined and treated for other concurrent illnesses.

Subjects will also receive medical attention and appropriate standard medical care or referral should they become ill during the study. This will be done at no cost to the subject.

27. Section 5 Study Population. New text has been added to this section describing the subject population for this study.

Malaria used to be endemic throughout Thailand. Overall, still 300,000 cases of malaria occur yearly, approximately 50% of which are Plasmodium vivax. Due to deforestation and other land use activities and a model public health control program, malaria is now limited to the mountainous forested and forest fringe regions along the border. The central plains of Thailand including [redacted] are largely malaria free, except for sporadic

cases in travelers. Historically, approximately 2/3 of volunteer subjects enrolled in research trials at the [redacted] are from residents of this malaria-free zone who acquired the infection while traveling to an endemic region, and who present to this hospital for evaluation and treatment of their febrile illness. These persons come from all walks of life, whose business or pleasure takes them in to malaria-endemic areas of the country.

A lesser number of volunteers may be recruited from a population of those who come to [redacted] for free clinical services from a [redacted] outpatient facility in [redacted] district, [redacted] Province, located about 2 hours from [redacted] The Tropical Medicine Trust Fund, under royal patronage by the king's sister, Her Royal Highness, Princess [redacted] established the satellite clinic of the [redacted] [redacted] in [redacted] in 1995 with a mandate to provide free treatment and prevention programs for tropical diseases both on-site, as well as free transportation and treatment as needed at the hospital in [redacted] The local population of [redacted] consists largely of Thai and Karen involved in farming, forestry or factory work with annual wages of 28000-36000 THB ($680-850). The mountainous areas of [redacted] are part of the traditional homeland of the Karen peoples, so here they will have Thai residency cards and speak the Thai language. Education to the eighth grade level is typical. These persons routinely use this [redacted] outpatient facility in [redacted] for medical care of a variety of tropical diseases, including malaria, leptospirosis and helminthiasis. A daily shuttle and regular ambulance service will bring anyone needing or desiring medical services from the [redacted] [redacted] outpatient facility to the [redacted] [redacted] for free diagnosis and treatment. Some are referred (e.g. severe malaria) or request to go to [redacted] for further evaluation and treatment. For example, in 2001, the [redacted] clinic treated 719 falciparum malaria patients; 229 of whom were transferred to [redacted] No recruitment for research trials occurs onsite in [redacted] but those who come to [redacted] for clinical care may be assessed for inclusion in any ongoing research trials at the [redacted] during their medical evaluation at the hospital.

28. Section 5.2.1 Inclusion Criteria, inclusion criterion number 5. Text changed to clarify subjects will be hospitalized for 29 days instead of 28 and to clarify that they will be asked to remain in a malaria free region as the term 'local' is not well-defined.

5. Willing to be hospitalized for 29 days and remain ~~local~~ in a malaria free region for 60 days thereafter for follow-up.

29. Section 5.2.1 Inclusion Criteria, inclusion criterion number 6a. Text pertaining to pre-menarchal subjects deleted.

a. non-childbearing potential (i.e., physiologically incapable of becoming pregnant), including any female who is ~~pre-menarchal or~~ post-menopausal or

30. Section 5.2.1 Inclusion Criteria, inclusion criterion number 6b. Text added at end of paragraph describing acceptable methods of birth control for this study.

Recognized contraceptive methods include, abstinence, implants of levonorgestrel, injectable progestogen, or appropriate double barrier methods using licensed contraceptives such as diaphragm and condom (by the partner) or intrauterine device and

condom. The use of oral/patch contraceptives during the study is not considered sufficient contraceptive protection.

31. Section 5.2.2 Exclusion Criteria, exclusion criterion number 1. Text changed to indicate malaria will be diagnosed using Field's stain instead of Giemsa only. A dipstick will not be used.

Was: 1. Mixed malaria infections by dipstick or Giemsa smear.

Is: 1. Mixed malaria infections by Field's stain.

32. Section 5.2.2 Exclusion Criteria, exclusion criterion number 8. Text added to correct omission of chloroquine.

5. History of allergy to chloroquine, mefloquine, tafenoquine, primaquine or any other 8-aminoquinolines.

33. Section 5.2.2 Exclusion Criteria. Additional exclusion criteria have been added, numbers 11, 12, and 13 to exclude subjects taking prohibited concomitant medications as originally indicated in Section 8.2 Prohibited Medications, to exclude subjects at risk for acute angle closure glaucoma, and to exludes females who are pre-menarchal.

11. Subjects taking concomitant medications likely to affect renal or ophthalmic function or that are known to be metabolized primarily by the cytochrome P450 isoforms 3A4/5 and 2C9 and whose therapeutic effect occurs within a narrow plasma concentration range (e.g. warfarin, ketoconazole).

12. Subjects whom, after examination by the study ophthalmologist, are judged to be at risk for acute angle closure glaucoma.

13. Females who are pre-menarchal.

34. Section 6.1 Demographic and Baseline Assessments, 2nd paragraph, 2nd sentence. Sentence has been modified to permit delaying the full eye exam if subject is deemed too ill to undergo the required testing. The window for completing the full eye exam after dosing has been increased from 24 hours to 36 hours to permit sufficient time for improvement of symptoms for these subjects, before undergoing the full eye examination.

Was: If the scheduling of the full eye exam will significantly delay the start of treatment (> 10 hours), a basic eye exam to assess vision problems will be performed prior to dosing, with a full eye exam to be conducted as soon as possible but no later than within 24 hours of receiving the first dose of study drug.

Is: If the scheduling of the full eye exam will significantly delay the start of treatment (> 10 hours) or if the subject is deemed by the investigator to be too ill to undergo the complete eye examination, a basic eye exam to assess vision problems will be performed prior to dosing. The full eye exam will then be conducted as soon as possible but no later than within 36 hours of receiving the first dose of study drug.

35. Section 6.1 Demographic and Baseline Assessments, after 2nd paragraph. A new paragraph has been added to clarify that subjects will be screened for the risk for acute angle closure glaucoma and will be excluded if at risk.

At the baseline examination the study ophthalmologists will measure a subject's intraocular pressure and anterior chamber depth during the standard eye examination. If the anterior chamber is shallow, gonioscopic examination of the chamber angle will be performed to exclude an occludable angle prior to dilating the pupil with mydriatics. Subjects at risk for acute angle closure glaucoma will be excluded from the study.

36. Section 6.2.1 Vital Signs, 1st paragraph. Text changed to indicate body temperature is to be measured every 12 hours.

Was: Vital signs (pulse rate, blood pressure, respiration rate, and temperature) will be measured and the results recorded in the eCRF at baseline and on Days 1-7, 14, 28, 60, and 90.

Is: Vital signs (pulse rate, blood pressure, respiration rate) will be measured and the results recorded in the eCRF at baseline and on Days 1-7, 14, 28, 60, and 90. Body temperature will be measured every 12 hours ($\pm$ 2 hours) after the baseline measurement through Day 7, and then daily on Days 14, 28, 60, and 90.

37. Section 6.2.2 Clinical Chemistry and Hematology. Section title changed to correct omission of methemoglobin.

Clinical Chemistry, <u>Methemoglobin,</u> and Hematology

38. Section 6.2.2 Clinical Chemistry and Hematology, 1st paragraph. Text added at end of paragraph to correct omission of methemoglobin as an assessment parameter.

A blood sample will be taken to determine methemoglobin concentration at baseline and Days 3, 7, and 21.

39. Section 6.2.6.1 Pregnancy Testing, 1st paragraph, 1st and 3rd sentences. Text added to clarify that pregnancy testing may be done via urine or serum.

All female subjects will undergo a pregnancy test <u>(urine or serum)</u> at baseline. Pregnant women will not be eligible for entry into the study. A pregnancy test <u>(urine or serum)</u> will also be performed on female subjects at the Day 7, Day 21, and Day 90 visit.

40. Section 6.2.6.3 Action to be taken if pregnancy occurs, 1st paragraph, 2nd sentence. Text added to indicate USAMRMC as well as GSK will be sent the pregnancy notification form.

The investigator, or his/her designee, will record pregnancy information on the appropriate form and submit it to <u>USAMRMC and</u> GSK within 2 weeks of learning of a subject's pregnancy.

41. Section 6.2.6.3 Action to be taken if pregnancy occurs, 1st paragraph, after 4th sentence. New sentence added to clarify subjects to be included in safety and ITT populations in case of a pregnancy.

Any subject who received at least one dose of study medication (i.e. tafenoquine or chloroquine/primaquine) and is withdrawn from the study due to pregnancy will be included in the Safety and ITT analysis populations.

42. Section 6.2.6.3 Action to be taken if pregnancy occurs, 1st paragraph, 6th sentence. Text added to indicate USAMRMC as well as GSK will be sent information on the status of the mother and child.

Information on the status of the mother and child will be forwarded to USAMRMC and GSK.

43. Section 6.2.6.3 Action to be taken if pregnancy occurs, 3rd paragraph, 2nd sentence. Text added to indicate USAMRMC as well as GSK will be sent reports on the indicated SAE.

Furthermore, any SAE occurring as a result of a post-study pregnancy **and** is considered reasonably related to the investigational product by the investigator, will be reported to USAMRMC and GSK as described in Section 10.11., "Post-study AEs and SAEs."

44. Section 6.3.1 Primary Efficacy, 1st paragraph, 1st sentence. Text added to allow for ±2 hour window around blood smears.

Thick and thin blood smears for malaria will be obtained by finger prick at baseline (Day 0) and then every 12 hours (± 2 hours) up to and including Day 7, until the blood smear becomes negative.

45. Section 6.3.1 Primary Efficacy, 1st paragraph, 5th sentence. Text added to sentence to clarify that these subjects will be included in the safety and ITT populations.

Subjects positive for parasitemia on any smear on Day 7 will be considered early treatment failures and withdrawn from the study and will be included in the Safety and ITT analysis populations.

46. Section 6.3.1 Primary Efficacy, 1st paragraph, 1st sentence. Text changed to indicate that malaria will be diagnosed using Field's stain instead of Giemsa.

All blood smears will be stained with Field's stain reagents and examined by two microscopists who are blinded to each other's results.

47. Section 6.3.1 Primary Efficacy, end of 3rd paragraph. Sentence added to clarify which subjects will be included in the safety and ITT populations.

All randomized subjects who received at least one dose of study medication (i.e. tafenoquine or chloroquine/primaquine) will be included in the Safety and ITT analysis populations.

48. Section 6.3.2 Secondary Efficacy Measures, 2nd paragraph, 1st sentence. Text added to allow for ±2 hour window around blood smears.

Parasite and gametocyte clearance time (PCT and GCT): Serial blood smears to detect the presence of *P. vivax* parasites and gametocytes, conducted , every 12 hours (± 2 hours) after the baseline smear up to an including Day 7, until the blood smear becomes negative will be utilized to determine the time (in half-days) to clearance from the time of initial study drug administration.

49. Section 6.3.2 Secondary Efficacy Measures, 3rd paragraph, 1st sentence. Text added to allow for ±2 hour window around temperature measurements.

Fever Clearance Time (FCT): Body temperature, measured every 12 hours (± 2 hours) after the baseline measurement through Day 7 will be used to determine the time (in half-days) from initiation of treatment until a subject's temperature decreases to 37.2 °C and remains at or below that level for a minimum of 24 hours.

50. Section 6.3.3. Efficacy Monitoring During Enrollment, 1st paragraph, 4th sentence. Text added to clarify IDMC will review all relevant safety and efficacy data.

The IDMC will be requested to review all relevant safety and efficacy data and to provide guidance as to how to proceed with enrollment. This may include recommending continuation, scheduling a second interim look, a change to the protocol, or stopping the study.

51. Section 6.3.3. Efficacy Monitoring During Enrollment, 2nd paragraph, after 2nd sentence. Additional sentence added providing rationale for using ETFs as the trigger for the IDMC review.

Using ETFs as the criteria for suspending enrollment and an IDMC review will allow determination of a true lack of efficacy at the earliest possible timepoint and thereby keep the number of subjects exposed to an ineffectual treatment regimen, to a minimum.

52. Section 6.4.1 Sample Collection, after bullet number vi. Additional bullet, vii, added to indicate that a PK sample will be taken for recurrences of *P. vivax*, after Day 7 up to and including Day 28.

vii At recurrence of *P. vivax* after Day 7 up to and including Day 28 if a PK sample has not been drawn within the previous 24 hours

53. Section 6.4.1 Sample Collection, last paragraph, 3rd sentence. Word "should" changed to "will" for clarity.

The **date** and exact **time** of each sample will be recorded on the sample tube, the case report form (CRF) and Quest requisition form. The **date** and exact **time** of administration of all doses must also be recorded in the CRF.

54. Section 6.4.1 Sample Collection, after last paragraph. New paragraph added providing additional instructions for taking PK samples at recurrence of *P. vivax*.

If *P. vivax* recurs after Day 7 up to and including Day 28, a PK sample will be taken to assess tafenoquine levels at the time of recurrence. This sample will be taken only if a PK sample has not been taken within the previous 24 hours. The **date** and exact **time** of the sample will be recorded on the sample tube, the case report form (CRF) and Quest requisition form.

55. Section 6.4.2 Processing of Samples, 1st paragraph, 2nd sentence. Word "should" changed to "will" for clarity and the word "approximately" added before 4°C to allow for some temperature variation.

The whole blood sample will be stored at approximately 4°C or on water ice until centrifuged, and must be centrifuged, separated and decanted within 2 hours of the sample collection.

56. Section 6.4.2 Processing of Samples, 1st paragraph, 4th and 5th sentences. Temperature changed from -20°C to -70°C to increase stability of samples.

Plasma samples will be stored frozen at approximately -70°C or colder until shipped. GlaxoSmithKline or its designee will store the plasma samples at approximately -70°C or colder until analyzed.

57. Section 6.4.2 Processing of Samples, 2nd paragraph, last sentence. Text modified to clarify role of Quest Diagnostics as co-ordinating the shipping of the pharmacokinetic samples.

Was: Samples should be shipped intermittently during the study period.

Is: Shipping of samples will be co-ordinated by Quest Diagnostics and should be shipped intermittently during the study period.

58. Section 6.5 Biomarkers renamed Other Studies to better reflect nature of tests. Additional text added to clarify tests to be done.

Was: Pretreatment (and relapse) aliquots of each subject's *P. vivax* infected blood will be cryopreserved in Dimethyl Sulfoxide (DMSO) or glycerol. Archiving viable parasites in this manner will potentially allow for future *in vitro* resistance testing. Sample processing procedures will be provided in a separate document.

Is: Pretreatment (and recurrence) aliquots of each subject's *P. vivax* infected blood will be cryopreserved in Dimethyl Sulfoxide (DMSO) or glycerol. Archiving viable parasites in this manner will potentially allow for future *in vitro* drug resistance testing. Samples may also be used to study the effect of the study drug(s) on parasite killing or inhibition. Archiving samples in this manner will also allow for future PCR or parasite genotyping studies, which may aid in determining the true efficacy of the study drug in the event of late treatment failure. Sample processing procedures will be provided in a separate document.

59. Section 7.1 Description of Investigational Product, following last paragraph. Manufacturing and packaging details of tafenoquine and comparators added.

The investigational drug product (tafenoquine) was manufactured by:
GlaxoSmithKline
Magpie Wood
Manor Royal
Crawley
Sussex
RH10 2QJ
UK

The commercial supplies of chloroquine and primaquine used for overencapsulation were sourced from:
1. Choroquine (as Avloclor* tablets containing 250 mg chloroquine phosphate)
AstraZeneca UK Ltd.

600 Capability Green
Luton
LU1 3LU
UK

2. Primaquine (as Primacin* tablets containing primaquine phosphate equivalent to 7.5 mg primaquine)
Boucher and Muir Pty Ltd
Willoughby Road
Crows Nest
New South Wales
NSW 2065
Australia

Packaging of the investigational drug product, as well as manufacture and packaging of the matching placebos and overencapsulated comparator products (chloroquine and primaquine) was performed by:
GlaxoSmithKline
Third Avenue
Harlow
Essex
CM19 5AW
UK

60. Section 7.2 Dosage and Administration, Cohort 2, chloroquine arm. Text modified to correct dosing regimen for subjects randomized to chloroquine/primaquine arm.

Was: Chloroquine: 4 capsules (250mg chloroquine phosphate/capsule for a total of 1000 mg chloroquine phosphate) and 2 tafenoquine placebo capsules for 2 days, followed by 2 chloroquine capsules (500mg chloroquine phosphate) and 2 tafenoquine placebo capsules for 1 day, followed by 1 primaquine capsule (15 mg base/capsule) per day for 14 days

Is: Chloroquine: 4 capsules (250mg chloroquine phosphate/capsule for a total of 1000 mg chloroquine phosphate) and 3 tafenoquine placebo capsules for 1 day, followed by 4 chloroquine capsules (1000 mg chloroquine phosphate) for one day, followed by 2 chloroquine capsules (500mg chloroquine phosphate) for one day, followed by 1 primaquine capsule (15 mg base/capsule) per day for 14 days.

61. Section 7.2 Dosage and Administration, end of last paragraph. Sentence added to clarify which subjects will be included in the safety and ITT populations.

All randomized subjects who received at least one dose of study medication (i.e. tafenoquine or chloroquine/primaquine) will be included in the Safety and ITT analysis populations.

62. Section 7.3 Dose Rationale, 1st paragraph, 3rd sentence. Text added to clarify the single 600 mg dose is comparable to 3 X 400 mg dose for tolerability, safety and efficacy for eradication of *P. vivax* hypnozoites.

Was: The 600 mg dose for one day, is the maximum single dose of tafenoquine administered to subjects and has also demonstrated efficacy for eradication of *P. vivax* hypnozoites.

Is: The 600 mg dose for one day, is the maximum single dose of tafenoquine administered to subjects and has demonstrated comparable tolerability, safety and efficacy for eradication of *P. vivax* hypnozoites, as the 400 mg dose for 3 days.

63. Section 7.3 Dose Rationale, new paragraph added after 1st paragraph. Text added to further clarify rationale for comparator arm.

A standard blood schizonticidal dosing regimen of chloroquine (1000 mg chloroquine phosphate for 2 days followed by 500 mg chloroquine phosphate for 1 day) followed by a standard hypnozoite eradication dosing regimen for primaquine (15 mg base per day for 14 days) will be used for the control arm in this study. As the safety of chloroquine for the treatment of malaria is well established, the control arm will be used to allow for subjective comparisons with tafenoquine for the occurrence of ophthalmic and renal effects and to help discern if tafenoquine exacerbates the occurrence and severity of the adverse events that are to be closely monitored during this study (nausea, diarrhea, and vomiting) which are often symptoms of malaria.

64. Section 7.4 Blinding, 1st and 2nd paragraphs. Text has been modified to clarify blinding and unblinding logistics which will be handled via RAMOS.

Was: This will be an active-control, double-blind, double-dummy study. Each randomized subject will be allocated the next (i.e. lowest) sequential treatment number. Neither the subject, the Investigator nor the study staff will know which treatment has been allocated.

In order to unblind a subject treatment the disclosure panel must be scratched with a coin.

Is: This will be an active-control, double-blind, double-dummy study, in which all subjects will receive both active and placebo study medication. Each randomized subject will be allocated the next (i.e. lowest) sequential treatment number and the corresponding medication pack will be assigned. Neither the subject, the Investigator nor the study staff will know which treatment has been allocated as all medication packs will be identical in appearance.

Emergency unblinding will be available via GSK's Registration and Medication Ordering System (RAMOS). To unblind a subject the site should call RAMOS using the usual toll-free number for that country. To identify the subject RAMOS will request the CRF number (subject number) or any of the previously dispensed container numbers for that subject. Detailed guidance will be provided in the study worksheet issued to each site before study start.

65. Section 7.4 Blinding, 3rd paragraph, 2nd sentence. Text added to indicate that USAMRMC as well as GSK must be informed if the study blind is broken.

If the blind is broken for any reason, the investigator must notify USAMRMC and GSK **immediately** of the unblinding incident without revealing the subject's study treatment assignment.

66. Section 7.5 Treatment Assignment, 1st paragraph. Text has been added to further describe the generation of the randomization schedule.

Was: Subjects will be assigned to study treatment in accordance with the randomization schedule. The randomization schedule will be produced by the GSK Study Statistician.

Is: Subjects will be assigned to study treatment in accordance with the randomization schedule.

Separate randomization lists will be generated for Cohorts 1 and 2 by a GSK statistician using the Coding Memo System (GSK randomization software package) to allocate subjects in a 2:1 ratio to the tafenoquine treatment group, using blocking. GSK will hold the master randomization list for each Cohort and the block size will remain confidential.

67. Section 7.7 Handling and Storage, 1st paragraph. Text modified to clarify that subjects will not be self-administering any study medication.

Was: Investigational product must be dispensed or administered according to procedures described herein. Only subjects enrolled in the study may receive investigational product, in accordance with all applicable regulatory requirements. Only authorized site staff may supply or administer investigational product. All investigational products must be stored in a secure area with access limited to the investigator and authorized site staff and under physical conditions that are consistent with investigational product-specific requirements.

Is: Investigational product must be administered according to procedures described herein. Only subjects enrolled in the study may be administered product, in accordance with all applicable regulatory requirements. Only authorized site staff may administer investigational product. All investigational products must be stored in a secure area with access limited to the investigator and authorized site staff and under physical conditions that are consistent with investigational product-specific requirements.

68. Section 7.8 Product Accountability. Text modified throughout to clarify that subjects will not be self-administering any study medication.

Was: The investigator or designee, upon receipt of the investigational product supplies, will conduct an inventory and sign both copies of the forms provided by GSK. One copy should be forwarded as instructed in the form and the other copy must be retained for the investigator's study records.

The treatment bottle will be the accountability unit. Where applicable, subjects will be instructed **not** to discard un-used study medication and to bring it with them to the next study visit.

The investigator, or designee, will record the dispensing of the investigational product to subjects and any subsequent returns or losses of drug supply. These records will be made available to clinical monitoring personnel. An investigational product supply inspection for inventory purposes and assurance of proper storage will be conducted at regular intervals throughout the clinical investigation. Any significant discrepancy and/or deficiency is to be recorded, reported to the Sponsor, and a plan for resolution is to be documented.

Is: The investigator is responsible for investigational product accountability, reconciliation, and record maintenance. In accordance with all applicable regulatory requirements, the investigator or designated site staff must maintain investigational product accountability records throughout the course of the study. This person(s) will document the amount of investigational product received from GSK, and the amounts administered to subjects.

The investigator or designee, upon receipt of the investigational product supplies, will conduct an inventory and sign both copies of the forms provided by GSK. One copy should be forwarded as instructed in the form and the other copy must be retained for the investigator's study records.

The treatment bottle will be the accountability unit.

The investigator, or designee, will record the administration of the investigational product to subjects and any subsequent returns or losses of drug supply. These records will be made available to clinical monitoring personnel. An investigational product supply inspection for inventory purposes and assurance of proper storage will be conducted at regular intervals throughout the clinical investigation. Any significant discrepancy and/or deficiency is to be recorded, reported to USAMRMC and GSK, and a plan for resolution is to be documented.

69. Section 8.1 Permitted Medications. A paragraph has been added at the beginning to clarify which medications are permitted.

The use of concomitant medications should be limited to those essential for the care of the subject. Other medication which the subject takes regularly, i.e., for control of chronic conditions, should be continued as directed by the subject's physician.

70. Section 8.2 Prohibited Medications, 2nd paragraph, last sentence. Additional text has been added to end of sentence to clarify that subjects will be treated if withdrawn during the treatment phase of the study.

Subjects who require such medications during the treatment phase of the study should be withdrawn from treatment and given appropriate anti-malarial treatment according to [redacted] guidelines.

71. Section 8.2 Prohibited Medications, 3rd paragraph. The paragraph has been modified to clarify that herbal medications are not permitted during the study.

Was: Subjects will be discouraged from taking any prescription or herbal medication during the study period and will be asked to contact the study clinic before

commencing any such medication. Details of all concomitant medications will be entered into the CRF.

Is: Subjects will be discouraged from taking any prescription medication during the study and will be asked to contact the study clinic before commencing any such medication. Subjects may not take any herbal medication during the study period. Details of all concomitant medications will be entered into the CRF.

72. Section 9.2 Subject Withdrawal, end of 3rd paragraph. A sentence has been added to clarify which subjects will be included in the safety and ITT populations.

All randomized subjects who received at least one dose of study medication (i.e. tafenoquine or chloroquine/primaquine) will be included in the Safety and ITT analysis populations.

73. Section 9.2.1 Subject Withdrawal from Study, end of 1st paragraph. A sentence has been added to clarify which subjects will be included in the safety and ITT populations.

All randomized subjects who received at least one dose of study medication (i.e. tafenoquine or chloroquine/primaquine) will be included in the Safety and ITT analysis populations.

74. Section 10.1 Definition of an AE, last paragraph, 1st sentence. Sentence has been modified with the deletion of the phrase “For GSK clinical studies” to reflect that this is a collaborative study with the USAMRMC.

Was: For GSK clinical studies, AEs may include pre- or post-treatment events that occur as a result of protocol-mandated procedures (i.e., invasive procedures, modification of subject’s previous therapeutic regimen).

Is: AEs may include pre- or post-treatment events that occur as a result of protocol-mandated procedures (i.e., invasive procedures, modification of subject’s previous therapeutic regimen).

75. Section 10.5 Time Period, Frequency, and Method of Detecting AEs and SAEs, 1st paragraph, first sentence. The words, “actively collected” were added to the text for clarity on AE assessments.

At each visit/assessment, AEs will be actively collected and evaluated by the investigator. AEs not previously documented in the study will be recorded directly into the electronic CRF.

76. Section 10.5 Time Period, Frequency, and Method of Detecting AEs and SAEs, 2nd paragraph, 1st sentence. The sentence was clarified to indicate the time period for collecting SAEs and AEs, by deleting the phrase “at randomization or”.

Was: The time period for the collection of SAE's and AE's will begin at randomization or first receipt of investigational product until Day 90.

Is: The time period for the collection of SAE's and AE's will begin at first receipt of investigational product until Day 90.

77. Section 10.5 Time Period, Frequency, and Method of Detecting AEs and SAEs, end of 2nd paragraph. Additional sentence has been added to clarify reporting of SAEs for the period Day 91-120.

For Days 91-120, any SAEs the investigator learns of, including a death, will be reported promptly.

78. Section 10.9.1 Timeframes for Submitting SAE Reports to USAMRMC and GSK. Table text changed to clarify SAEs to reported to USAMRMC and GSK.

Was:

	Initial SAE Reports		Follow-up Information on a Previously Reported SAE	
Type of SAE	Time Frame	Documents	Time Frame	Documents
All SAEs	24 hrs	1. Paper SAE forms to be faxed to GSK.<br>2) eCRF data to be transferred to GSK (replicated)	24 hrs	1. Updated paper "SAE" form to be faxed to GSK.<br>2) Updated eCRF data to be transferred to GSK (replicated)

Is:

	Initial SAE Reports		Follow-up Information on a Previously Reported SAE	
Type of SAE	Time Frame	Documents	Time Frame	Documents
All SAEs	24 hrs	1. Paper SAE forms to be faxed to USAMRMC and GSK<br>2) eCRF data to be transferred to GSK (replicated)	24 hrs	1. Updated paper "SAE" form to be faxed to USAMRMC and GSK.<br>2) Updated eCRF data to be transferred to GSK (replicated)

79. Section 10.9.2 Completion and Transmission of the SAE Reports. This section has been subdivided into 2 sub-sections. Section 10.9.2.1 Completion and Transmission of the SAE Reports to GSK, and Section 10.9.2.2 Completion and Transmission of the SAE Reports to USAMRMC. The text for section 10.9.2.1 remains the same except for the USAMRMC and GSK text replacements for "GSK" (see change number 106), and the addition of the following sentence at the end of the 1st paragraph, to clarify the timeframe for reporting follow-up information for a SAE.

<u>Follow-up information should be forwarded to GSK as described in section 10.8.</u>

In addition the last paragraph of Section 10.9.2.1 was modified to clarify what "Form D" is.

Was: The following sections of the electronic CRF must accompany the SAE forms that are forwarded to GSK: "Demography", "Medical History", "Concomitant Medications", "Study Medication Records", and "Form D" (if applicable)

Is: The following sections of the electronic CRF must accompany the SAE forms that are forwarded to GSK: "Demography", "Medical History", "Concomitant Medications", "Study Medication Records", and death notification form "Form D" (if applicable).

The text for section 10.9.2.2 Completion and Transmission of the SAE Reports to USAMRMC reads as follows:

Adverse experiences that are both serious and unexpected will be immediately reported by telephone to the USAMRMC Office of Regulatory Compliance and Quality [redacted] (non-duty hours call [redacted] and send information by facsimile to [redacted] A written report will follow the initial telephone call within 3 working days. Address the written report to the U.S. Army Medical Research and Materiel Command, ATTN: MCMR-RCQ, 504 Scott Street, Fort Detrick, Maryland 21702-5012.

80. Section 11.4.1 Sample Size Assumptions, 2nd paragraph, 3rd sentence. Sentence modified as original sentence was not complete.

Was: With 40 evaluable tafenoquine subjects within a cohort, the lower limit of the one-sided 95% confidence interval for the Day 28 cure rate, if the number of failures on the primary endpoint ranges between 0 and 3.

Is: With 40 evaluable tafenoquine subjects within a cohort, the lower limit of the one-sided 95% confidence interval for the Day 28 cure rate ranges from 92.8% for 0 failures to 81.7% for 3 failures.

81. Section 11.4.2 Sample Size Sensitivity, Safety sub-section, 1st paragraph, 1st bullet. Abbreviation GFR deleted and spelled out as glomerular filtration rate, as this is the only occurrence in the document.

82. Section 11.5 Analysis Populations, 3rd paragraph. Abbreviation ITT added following Intent to Treat for clarity.

Intent to Treat (ITT) Population: all randomized subjects who received at least one dose of study medication (i.e. tafenoquine or chloroquine/primaquine).

83. Section 11.5 Analysis Populations, 5th paragraph. Abbreviation PP added following Per Protocol to define this abbreviation at first occurrence.

Per Protocol (PP) Populations:

84. Section 11.6.3 Derived and Transformed Data, 2nd paragraph, 1st sentence. Text added to allow for ±2 hour window around blood smears.

Serial blood smears, conducted every 12 hours (± 2 hours) after the baseline smear to detect the presence of *P. vivax* parasites and gametocytes (Days 1, 2, 3, 4, 5, 6, and 7) will be utilized to determine the time to clearance (date and time of first blood smear testing negative for *P. vivax* parasites/gametocytes) – (date and time of first treatment)+1

85. Section 11.6.3 Derived and Transformed Data, 4th paragraph, 1st sentence. Text changed to indicate body temperature is to be measured every 12 hours.

Body temperature, measured every 12 hours (± 2 hours) after the baseline measurement through Day 7 will be used to determine the Fever Clearance Time (FCT), defined as the time (in half-days) from initiation of treatment until a subject's temperature decreases to 37.2 °C and remains at or below that level for a minimum of 24 hours:

86. Section 12.1.2 Ethical Conduct of the Study and Ethics Approval, 2nd paragraph. Text added to clarify that the [redacted] along with the local IEC/IRB will review and approve required documents for this study.

Was: The investigator (or sponsor, where applicable) is responsible for ensuring that this protocol, the site's informed consent form, and any other information that will be presented to potential subjects (e.g., advertisements or information that supports or supplements the informed consent) are reviewed and approved by the appropriate IEC/IRB. The investigator agrees to allow the IEC/IRB direct access to all relevant documents. The IEC/IRB must be constituted in accordance with all applicable regulatory requirements. GSK will provide the investigator with relevant document(s)/data that are needed for IEC/IRB review and approval of the study. Before investigational product(s) and CRFs can be shipped to the site, GSK must receive copies of the IEC/IRB approval, the approved informed consent form, and any other information that the IEC/IRB has approved for presentation to potential subjects.

Is: The investigator (or USAMRMC and GSK, where applicable) is responsible for ensuring that this protocol, the site's informed consent form, and any other information that will be presented to potential subjects (e.g., advertisements or information that supports or supplements the informed consent) are reviewed and approved by the appropriate IEC/IRBs and [redacted] The investigator agrees to allow the IEC/IRB and [redacted] direct access to all relevant documents. The IEC/IRB must be constituted in accordance with all applicable regulatory requirements. GSK will provide the investigator with relevant document(s)/data that are needed for IEC/IRB and [redacted] review and approval of the study. Before investigational product(s) and CRFs can be shipped to the site, GSK must receive copies of the IEC/IRB and [redacted] approval, the approved informed consent form, and any other information that the IEC/IRB and [redacted] has approved for presentation to potential subjects.

87. Section 12.1.2 Ethical Conduct of the Study and Ethics Approval, 3rd paragraph, 1st sentence. Text added to clarify that all modifications, not just amendments to the protocol will be reviewed and approved by the IEC/IRB and [redacted] prior to initiation..

Was: If the protocol, the informed consent form, or any other information that the IEC/IRB has approved for presentation to potential subjects is amended during the study, the investigator is responsible for ensuring the IEC/IRB reviews and approves, where applicable, these amended documents. The investigator must follow all applicable regulatory requirements pertaining to the use of an amended informed consent form including obtaining IEC/IRB approval of the amended form before new subjects consent to take part in the study using this version of the form. Copies of the IEC/IRB approval of the amended informed

consent form/other information and the approved amended informed consent form/other information must be forwarded to GSK promptly.

Is: For all modifications to the protocol, the informed consent form, or any other information that the IEC/IRB and [redacted] have approved for presentation to potential subjects, the investigator is responsible for ensuring the IEC/IRB and [redacted] reviews and approves, where applicable, these amended documents prior to initiation. The investigator must follow all applicable regulatory requirements pertaining to the use of an amended informed consent form including obtaining IEC/IRB and [redacted] approval of the amended form before new subjects consent to take part in the study using this version of the form. Copies of the IEC/IRB and [redacted] approval of the amended informed consent form/other information and the approved amended informed consent form/other information must be forwarded to GSK promptly.

88. Section 12.1.2 Ethical Conduct of the Study and Ethics Approval, last paragraph. Paragraph was deleted as it does not apply to this study.

Paragraph Deleted: ~~IEC/IRB approval of the PG consent forms must be obtained in addition to the approval given for the clinical study. Regulatory review and approval may be required in some countries before IEC/IRB approval can be sought.~~

89. Section 12.1.3 Informed Consent, after 1st paragraph, 1st sentence. Text added to clarify that informed consent will be obtained by investigator or designee. Additional paragraph added to provide additional information on the informed consent process.

Informed consent will be obtained <u>by the Investigator or designee</u> before the subject can participate in the study. The contents and process of obtaining informed consent will be in accordance with all applicable regulatory requirements.

The informed consent process will be initated after evaluation and diagnosis of subjects presenting to the [redacted] The consent process will be initiated by a [redacted] study nurse or investigator in a private, one on one conversation. Each potential subject must be adequately informed of the aims, methods, anticipated benefits and potential hazards of the study and the discomfort it may entail. The subject will be informed that they are at liberty to abstain from participation in the study and that if they choose not to participate they will receive the standard of care antimalarial medication and will be discharged when medically warranted. A subject will also be informed that they may discontinue participation at any time without penalty or loss of benefits to which the subject is otherwise entitled. Transportation back home (for [redacted] residents) will be provided free of charge with financial support from the Tropical Medicine Trust Fund. The subject will be instructed to take time to read the information in the consent form carefully, to discuss it with friends, relatives and their personal doctor, and to take time to decide whether or not they wish to participate. The subject will be encouraged to ask questions if there is anything that is not clear to them. The informed consent should be freely given by the subject, preferably in writing.

90. New Section 12.2 Medical Monitor. A new section was added describing the role of the independent medical monitor for this study.

12.2. Medical Monitor

A medical monitor will be assigned to this study. The name and curriculum vitae of the medical monitor will be provided. This individual will be a qualified physician, other than the Principal Investigator, who is not associated with this particular protocol, and will be able to provide medical care to research subjects for conditions that may arise during the conduct of the study, and who will monitor the subjects during the conduct of the study. The medical monitor is required to review all serious and unexpected adverse events (per ICH definitions) associated with the protocol and provide an unbiased written report of the event within 10 calendar days of the initial report to the [redacted] At a minimum, the medical monitor should comment on the outcomes of the adverse event (AE) and relationship of the AE to the study medication. The medical monitor should also indicate whether he/she concurs with the details of the report provided by the study investigator. The Medical Monitor is also required to submit AE findings to the [redacted] the Sponsor's Representative will relay these reports to the [redacted]

91. Section 12.3 Study Monitoring, 1st paragraph. Text has been modified to clarify monitoring roles during the study.

Was: In accordance with applicable regulations, GCP, and GSK procedures, GSK monitors will contact the site prior to the subject enrollment to review the protocol and data collection procedures with site staff. In addition, the monitor will periodically contact the site, including conducting on-site visits. The extent, nature and frequency of on-site visits will be based on such considerations as the study objective and/or endpoints, the purpose of the study, study design complexity, and enrollment rate.

Is: Monitoring responsibilities for this protocol will be performed by USAMRMC's Quality Assurance Office, and the Division of Microbiology and Infectious Diseases (DMID). In accordance with applicable regulations, GCP, and USAMRMC and GSK procedures, monitors will contact the site prior to the subject enrollment to review the protocol and data collection procedures with site staff. Monitoring visits will be scheduled to take place before entry of the first subject, during the study at appropriate intervals and after the last subject is completed. A Pre-Study/Initiation visit will be conducted with monitors from USAMRMC and DMID. In addition the monitor(s) will periodically contact the site, including conducting on-site visits. The extent, nature and frequency of on-site visits will be based on such considerations as the study objective and/or endpoints, the purpose of the study, study design complexity, and enrollment rate. At least one of these visits will be conducted by USAMRMC Quality Assurance Office and the rest will be conducted by monitors from DMID. The Closeout monitoring visit will be conducted by monitors from both USAMRMC Quality Assurance Office and DMID. Monitoring Reports will be provided to USAMRMC Quality Assurance Office and GlaxoSmithKline after each monitoring visit.

92. Section 12.4 Quality Assurance, 1st paragraph, 1st sentence. Text added to include USAMRMC and DMID as entities which may conduct quality assurance audits.

Was: To ensure compliance with GCP and all applicable regulatory requirements, GSK may conduct a quality assurance audit. Regulatory agencies may also conduct a regulatory inspection of this study.

Is: To ensure compliance with GCP and all applicable regulatory requirements, USAMRMC, GSK, and DMID/National Institute of Allergy and Infectious Diseases (NIAID) may conduct a quality assurance audit.

93. Section 12.5 Study and Site Closure, 1st paragraph, 1st sentence. Text modified to clarify site closure activities of the monitor.

Upon completion of the study, the monitor will ~~conduct~~ confirm that the following activities have been conducted, in conjunction with the investigator or site staff, as appropriate:

94. Section 12.5 Study and Site Closure, 2nd paragraph, text deleted for clarity since this is a single site study.

In addition, USAMRMC and GSK reserve~~s~~ the right to temporarily suspend or prematurely discontinue this study ~~either at a single site or at all sites~~ at any time for reasons including, but are not limited to, safety or ethical issues or severe non-compliance.

95. Section 12.6 Records Retention, after 2nd paragraph. A new paragraph has been added detailing the USAMRMC Volunteer Registry Database requirement

It is the policy of the U.S. Army Medical and Materiel Command that data sheets are to be completed on all subjects participating in research for entry into the Command's Volunteer Registry Database. This is a confidential database and the data entered includes name, address, social security or equivalent identification number and details of the clinical study. This information is needed to answer questions concerning subjects participating in research sponsored by USAMRMC, and to ensure that subjects can be contacted if there is new information on the study drug. The information should be stored for 75 years. This information will be collected once the subject has agreed to participate in the study and signed the informed consent.

96. Section 12.7 Provision of Study Results and Information to Investigators. The following paragraph was deleted as it does not pertain to this study.

~~GSK will not routinely inform the investigator or subject of the test results, because the information generated from this study will be preliminary in nature, and the significance and scientific validity of the results will be undetermined at such an early stage of research. Individual genotype results will only be shared with an investigator or subject if this is a requirement of a governmental agency or other legal authority.~~

97. Section 12.8 Information Disclosure and Inventions, sub-section Publication: The following paragraph was deleted as it does not pertain to this study.

~~For multicenter studies, the first publication or disclosure of study results shall be a complete, joint multicenter publication or disclosure coordinated by GSK. Thereafter, any secondary publications will reference the original publication(s).~~

98. Section 12.9 Data Management, sub-section Data Security. Text added after 1st sentence describing measures to secure eCRF data.

The computer equipment will be maintained in good working condition, in a secured area with limited access, and used exclusively for the collection of clinical data for this study. Access to the eCRF will be password-controlled. Unique password(s) will be assigned to the appropriate site personnel at the time of training or installation. All subject data transmitted during the conduct of the study will be identified only by the unique subject number. Additional subject data (name, initials, etc.) collected for use by the clinical site personnel are not transmitted and so are not entered into the GSK database.

99. Section 12.10 Independent Data Monitoring Committee (IDMC), last paragraph, 2nd sentence. Text added to clarify that IDMC will be reviewing all relevant safety and efficacy data.

The IDMC will review all the available relevant safety and efficacy study data and advise on the appropriateness of continuing the study

100. Appendix 1: [Time and Events Table], footnote 1 for both Cohort 1 and Cohort 2 changed to allow for ± 2 hours window around blood smears.

Blood smears to be conducted every 12 hours (± 2 hours) until negative for 2 consecutive smears. Once confirmed will be obtained once a day up to and including Day 7.

101. Appendix 1: [Time and Events Table], footnote 2 for both Cohort 1 and Cohort 2 changed to allow for ± 2 hours window around body temperature measurements.

Body temperature to be measured every 12 hours (± 2 hours)

102. Appendix 1: [Time and Events Table], footnote 3 for both Cohort 1 and Cohort 2 changed to indicate baseline eye exams should be performed within 36 hours instead of 24.

103. Appendix 1: [Time and Events Table], footnote 4 for both Cohort 1 and Cohort 2 changed to indicate all SAEs the physician becomes aware of will be reported.

Serious Adverse events only.~~, if related to study drug or study participation~~

104. Appendix 3 Country Specific Requirements has been renamed Organization of Research Effort and now lists the roles and contact information for study personnel involved in the conduct of study 058.

105. Appendix 4 Research Facilities and IEC/IRBs has been added with the contact information for the research facilities involved in the conduct of study 058 and the contact information for the relevant IEC/IRBs for this study.

106. Throughout the protocol clarification has been added to indicate when USAMRMC and GSK are involved. The words “GSK” and “Sponsor” were replaced with USAMRMC and GSK at the follow places in the protocol:

Investigator Protocol Agreement Page, the 2nd, 3rd,4th, and 7th bullets, and the 1st and 3rd sub-bullets under the 7th bullet.

Section 9.2 Subject Withdrawal, 2nd to last bullet.

Section 10.7.2 Assessment of Causality, 2nd paragraph, 1st and 2nd sentences.

Section 10.8 Follow-Up of AEs and SAEs, 1st paragraph, 1st sentence, 4th paragraph, 1st and 3rd sentences.

Section 10.9, title changed to Prompt Reporting of SAEs to USAMRMC and GSK

Section 10.9 Prompt Reporting of SAEs to USAMRMC and GSK, 1st paragraph, 1st sentence.

Section 10.9.1, title changed to Timeframes for Submitting SAE Reports to USAMRMC and GSK.

Section 10.9.2.1 1st paragraph, 1st and 2nd sentences. 3rd paragraph, 3rd sentence.

Section 10.10. Regulatory Reporting Requirements for SAEs, 1st paragraph, 1st and 2nd sentences.

Section 10.11 Post-study AEs and SAEs, 2nd paragraph, 2nd sentence.

Section 10.12 SAEs Related to Study Participation, 1st sentence.

Section 12.1.2. Ethical Conduct of the Study and Ethics, 2nd paragraph, 1st sentence.

Section 12.1.4. Investigator Reporting Requirements, 1st paragraph, 1st sentence.

Section 12.5. Study and Site Closure, 2nd paragraph, 1st, 2nd, and 3rd sentences and 3rd paragraph, 1st sentence and 5th paragraph, 1st sentence.

Section 12.6 Records Retention, 2nd paragraph, 1st and 2nd sentences and 4th paragraph, 1st sentence.

Section 12.7. Provision of Study Results and Information to Investigators, 1st paragraph, 1st sentence.

Section 12.8. Information Disclosure and Inventions, sub-section Ownership, 1st paragraph,1st sentence, 2nd paragraph, 2nd sentence, 3rd paragraph, 1st sentence.

Section 12.8. Information Disclosure and Inventions, sub-section Confidentiality, 1st paragraph, 1st sentence.

Section 12.8. Information Disclosure and Inventions, sub-section Publication, 1st paragraph, 1st and 2nd sentences. 2nd paragraph, 1st sentence.

Protocol Amendment 02 Changes

The following change was made to the original protocol, approved 12 March 2003, and amended 16 July 2003 for study SB252263/058: A randomized, active-control, double-blind, double-dummy study to evaluate the efficacy and safety of tafenoquine for the treatment of *Plasmodium vivax* in adults.

1. Section 6.3.1 Primary Efficacy, 2nd paragraph. 2nd sentence. Calculating parasite densities based on a count of parasites per 200 white blood cells (WBC) on a thick film is not practical if the parasite count is high. In these situations, calculation of parasite densities based on a count of parasites per 1000 red blood cells.(RBC) on a thin film is an acceptable and standard method. Therefore the sentence has been

modified to indicate that parasite densities may be calculated by parasites per 200 WBC on a thick film or 1000 RBC on a thin film.

Was: Parasite densities will be calculated based on a count of parasites per 200 WBC.

Is: Parasite densities will be calculated based on a count of parasites per 200 WBC on a thick film or parasites per 1000 red blood cells (RBC) on a thin film.

Protocol Amendment 03 Changes

The following changes have been made to the original protocol, approved 12 March 2003, and amended 16 July 2003, and 18 August 2003 for study SB252263/058: A randomized, active-control, double-blind, double-dummy study to evaluate the efficacy and safety of tafenoquine for the treatment of *Plasmodium vivax* in adults.

1. Section 4.1 General Study Design 3rd paragraph. Text has been added to indicate that subjects transferred to [redacted] from rural malaria clinics from [redacted] or [redacted] provinces for evaluation of fever, may also be recruited for the study. The text added is underlined.

    Study volunteers will be recruited by a study investigator or nurse, from persons presenting either at the [redacted] outpatient clinic or admitted to the inpatient ward following transfer from the [redacted] clinic, or clinics from [redacted] or [redacted] provinces, for evaluation of fever. Subjects deemed eligible for the study will be consented and enrolled if eligible and willing to participate. Subjects found not eligible or unwilling to participate in the study will be treated as clinically warranted. Subjects originally transported from the [redacted] clinic, or clinics from [redacted] or [redacted] provinces, who are found not eligible or unwilling to participate in the study, will receive free treatment as clinically warranted and provided free transportation back to [redacted]-their respective rural malaria clinics when clinically warranted. Subjects enrolled into the study will be hospitalized at the [redacted] for the first 29 days of the study and will be asked to remain in a malaria non-endemic area until 90 days after the start of the study for follow up with scheduled assessments at Days 60 and 90. While hospitalized subjects enrolled in this study will have the same [redacted] visitation policy as are those subjects who are not research participants.

2. Section 5 Study Population after 2nd paragraph. A new paragraph has been added providing information on the rural malaria clinics in [redacted] and [redacted] provinces as subjects presenting to these clinics for the evaluation of fever and requesting further treatment and evaluation at the [redacted] may be recruited for participation in this study.

As a recognized facility specializing in the care of patients suffering from tropical infectious diseases, the [redacted] also serves as a referral tertiary care facility providing care to patients presenting to government operated rural malaria clinics located along the Thai-Myanmar border. Typically, such clinics are located in small villages in [redacted] and [redacted] provinces, approximately 4-7 hours from [redacted] Although malaria clinics are adequate for the routine diagnosis and treatment of malaria, these facilities operate as outpatient facilities only. As is the case with patients originating from the [redacted] facility, some patients presenting to these government malaria clinics may request further treatment and evaluation at the [redacted] In this event, the same [redacted] operated ambulance service will be employed to transport the patient from the malaria clinic to [redacted] Although no recruitment for research trials occurs in the malaria clinics, such patients may elect to participate in research trials while undergoing evaluation and treatment at [redacted]

3. Section 11.3.1 Planned Interim Analysis After Cohort 1 retitled Planned Interim Analysis After Cohort 1 Day 28 Assessment. Changes were made to the first paragraph to indicate that the planned interim analysis, originally to be conducted after all subjects in Cohort 1 had completed the Day 90 assessment will now be conducted after all subjects in Cohort 1 have completed the Day 28 assessment. In addition, the interim analysis, originally planned to be conducted internally by GSK, will now be prepared by an independent Statisical Data Analysis Center with the evaluation of the data to be conducted by an Independent Data Monitoring Committee. This change is being implemented to allow the timely completion of this study by permitting initiation of Cohort 2 during the peak malaria season in Thailand. Changing the interim analysis to Day 28 data, does not compromise the safety of the subjects, as the protocol involves a three day dosing schedule (completed on Day 2 of the study), hence, safety concerns for this dosing regimen of tafenoquine (nausea, vomiting, diarrhea, methemoglobin, renal effects, and ophthalmic effects-the adverse events which have been noted occasionally in over 2000 subjects exposed to tafenoquine in several previous studies) are likely to occur on or before the Day 28 assessment, if at all. Furthermore, Day 28 efficacy data should provide a reasonable measure of the ability of tafenoquine to eradicate acute clinical malaria due to *P. vivax* parasitemia . The later evaluation timepoints are designed to capture any possible recrudescence or relapse due to hypnozoites, which are anticipated to be infrequent based on results from previous studies. Hence, Day 28 data is believed to provide ample basis for initiating the second dose regimen phase of this protocol.

   The second paragraph of this section was deleted as it was not longer relevant since the interim analysis is to be conducted by an independent Statistical Data Analysis Center and reviewed only by the IDMC.

Was: 11.3.1 Planned Interim Analysis After Cohort 1

A planned interim analysis will be performed after completion of Cohort 1. This interim analysis will be based on all efficacy and safety data up to and including the Day 90 assessment. Data will be presented appropriately either as individual subject data listings, or as data summaries by treatment group. The

format of these data presentations will be defined in the RAP. All data analyses conducted for this interim analysis will be prepared internally by GSK. Only if the results from Cohort 1 meet pre-defined efficacy and safety criteria will enrollment begin for Cohort 2. The efficacy criterion for achieving the primary endpoint is that the lower limit of the one-sided 95% confidence interval is no less than 85%, and for safety that a review of trends in all AEs, tolerability, medical observations, methemoglobin and other lab data for all subjects indicates the dose is safe and well tolerated. The data for Cohort 2, together with the Day 120 parasitological assessment for Cohort 1, will be reported separately at the end of the study, as described in Sections 11.7,11.8, and 11.9 and will be conducted by GSK.

It should be noted that there is a possibility that by the time of unblinding and reporting of all Cohort 1 data up to Day 90, subjects from this Cohort will still be undergoing their Day 120 parasitological assessment. However the investigator will remain blinded at this stage.

Is: 11.3.1. Planned Interim Analysis After Cohort 1 Day 28 Assessment

A planned interim analysis will be performed after all subjects in Cohort 1 have completed the Day 28 assessment. This interim analysis will be based on all efficacy and safety data up to and including the Day 28 assessment. Data will be presented appropriately either as individual subject data listings, or as data summaries by treatment group. The format of these data presentations will be defined in the RAP. All data analyses conducted for this interim analysis will be prepared by an independent Statistical Data Analysis Center to ensure that GSK study related personnel remain blinded to treatment codes until the study is complete. An Independent Data Monitoring Committee (IDMC) will convene to evaluate the efficacy and safety of the tafenoquine dosing regimen (400 mg tafenoquine once per day for 3 days) used in Cohort 1. Only if the results from Cohort 1 meet pre-defined efficacy and safety criteria will enrollment begin for Cohort 2. The efficacy criterion for achieving the primary endpoint is that the lower limit of the one-sided 95% confidence interval is no less than 85%, and for safety that a review of trends in all AEs, tolerability, medical observations, methemoglobin and other lab data for all subjects indicates the dose is safe and well tolerated. The IDMC will review all the available relevant study data and advise on the appropriateness of initiating enrollment for Cohort 2. The data for Cohort 2, together with the Day 60, Day 90, and Day 120 safety and parasitological assessments for Cohort 1, will be reported separately at the end of the study, as described in Sections 11.7,11.8, and 11.9 and will be conducted by GSK.

4. Section 12.10 Independent Data Monitoring Committee (IDMC), was modified to indicate that the IDMC would have the additional responsibility of reviewing all relevant efficacy and safety data from the planned interim analysis and advise on the appropriateness of initiating enrollment for Cohort 2.

Was: 12.10 Independent Data Monitoring Committee (IDMC)

An IDMC will be utilized during the conduct of this study. An IDMC is generally assembled when there are significant safety or efficacy issues that warrant external objective medical and/or statistical review in order to protect the ethical and safety interests of subjects and to protect the scientific validity of the study. A copy of the IDMC charter is available from GSK upon request. It details the full responsibilities of the IDMC and a copy will be made available to the investigator.

The IDMC will consist of experts in nephrology, ophthalmology and malaria and a statistician from an independent SDAC, and will assist in the review of ongoing efficacy and safety data from this study if any of the following criteria are fulfilled:

- If ≥4 Early Treatment Failures (ETFs) occur among the first 21 subjects enrolled into the Cohort and an independent Statistical Data Analysis Center (SDAC) confirms that the actual response rate among evaluable tafenoquine subjects is below 70%
- A sustained (≥ 14 days) serum creatinine increase of ≥26.6 μmol/L (0.3 mg/dL) is confirmed in ≥8 subjects
- If at any timepoint, retinal changes from baseline that cause clinical concern (these would include decreased vision, bull's eye retinopathy, distortions observed on the Amsler Grid test, abnormal color vision or development of a scotoma on visual field testing) are seen in ≥2 subjects
- If ≥5 subjects have the following same drug related AE (based on body systems) of severe intensity:
- Methemoglobin concentration of ≥ 20%
- Repeated (> 4 times within one day) or intractable vomiting
- Severe diarrhea resulting in loss of intravascular volume judged by the investigator to be sufficiently severe to warrant the initiation or increase in rate of administration of intravenous replacement fluids after onset of diarrhea
- Nausea resulting in no significant oral intake, requiring intravenous fluids

Enrollment of further subjects will be put on hold and an *ad hoc* meeting of the IDMC will be called. The IDMC will review all the available relevant safety and efficacy study data and advise on the appropriateness of continuing the study. Outputs for an IDMC will be produced by an independent SDAC appointed by GSK. All GSK study related personnel will remain blinded to the treatment codes until the planned analysis for each cohort is complete.

Is: **12.10 Independent Data Monitoring Committee (IDMC)**

An IDMC will be utilized during the conduct of this study. An IDMC is generally assembled when there are significant safety or efficacy issues that warrant external objective medical and/or statistical review in order to protect the ethical and safety interests of subjects and to protect the scientific validity of the study. A copy of the IDMC charter is available from GSK upon request. It details the full responsibilities of the IDMC and a copy will be made available to the investigator.

The IDMC will consist of experts in nephrology, ophthalmology and malaria and a statistician from an independent SDAC, and will assist in the following:

1. Review of all relevant efficacy and safety data from a planned interim analysis after all subjects in Cohort 1 have completed the Day 28 assessment. The IDMC will determine if pre-defined efficacy and safety criteria, as described in Section 11.3.1 have been met and advise on the appropriateness of initiating enrollment for Cohort 2.

2. Review of ongoing efficacy and safety data from this study if any of the following criteria are fulfilled:

- If ≥4 Early Treatment Failures (ETFs) occur among the first 21 subjects enrolled into the Cohort and an independent Statistical Data Analysis Center (SDAC) confirms that the actual response rate among evaluable tafenoquine subjects is below 70%
- A sustained (≥ 14 days) serum creatinine increase of ≥26.6 μmol/L (0.3 mg/dL) is confirmed in ≥8 subjects
- If at any timepoint, retinal changes from baseline that cause clinical concern (these would include decreased vision, bull's eye retinopathy, distortions observed on the Amsler Grid test, abnormal color vision or development of a scotoma on visual field testing) are seen in ≥2 subjects
- If ≥5 subjects have the following same drug related AE (based on body systems) of severe intensity:
- Methemoglobin concentration of ≥ 20%
- Repeated (> 4 times within one day) or intractable vomiting
- Severe diarrhea resulting in loss of intravascular volume judged by the investigator to be sufficiently severe to warrant the initiation or increase in rate of administration of intravenous replacement fluids after onset of diarrhea
- Nausea resulting in no significant oral intake, requiring intravenous fluids

Enrollment of further subjects will be put on hold and an *ad hoc* meeting of the IDMC will be called. The IDMC will review all the available relevant safety and efficacy study data and advise on the appropriateness of continuing the

study. Outputs for an IDMC will be produced by an independent SDAC appointed by GSK. All GSK study related personnel will remain blinded to the treatment codes for both cohorts until the end of the study.
